# Extracellular matrix reprogramming by the YAP/TAZ/TGF-β2 axis drives immune exclusion in cholangiocarcinoma models

**DOI:** 10.1172/JCI201579

**Published:** 2026-07-02

**Authors:** Marco Jessen, KyungMok Kim, Marie Tollot-Wegner, Anita Cindric Vranesic, Cagla Dönmez, Celina Junker, Tina Lehmann, Advitiya Khandelwal, Yuliya Kurlishchuk, Tom Hünniger, Christin Ritter, Evaristo Di Napoli, Shyam Krishnan Murali, Konrad Bücking, Viktoria Haug, Sabine Muth, Tracy T. Tang, Andreas Rosenwald, Markus Radsak, Donato Inverso, Tanja Deckert-Gaudig, Volker Deckert, Orlando Paciello, Björn von Eyss

**Affiliations:** 1 Transcriptional Control of Tissue Homeostasis Lab, Leibniz Institute on Aging, Fritz Lipmann Institute e.V. (FLI), Jena, Germany.; 2 Department of Veterinary Medicine and Animal Production, University of Naples Federico II, Naples, Italy.; 3 Institute of Pathology, University of Würzburg, Würzburg, Germany.; 4 Institute of Immunology and Research Center for Immunotherapy (FZI), University Medical Center of the Johannes Gutenberg – University Mainz, Mainz, Germany.; 5 Vivace Therapeutics Inc., San Mateo, California, USA.; 6 Department of Hematology and Oncology and Research Center for Immunotherapy (FZI), University Medical Center of the Johannes Gutenberg-University, Mainz, Germany.; 7 Department of Medicine VII – Hematology and Oncology, Donau-Isar-Klinikum, Deggendorf, Germany.; 8 Vascular Pathobiology Unit, Vita-Salute San Raffaele University, Milan, Italy.; 9 Leibniz Institute of Photonic Technology, Jena, Germany.; 10 Institute of Physical Chemistry and Abbe Center of Photonics, Friedrich-Schiller University Jena, Jena, Germany.

**Keywords:** Hepatology, Oncology, Cancer immunotherapy, Extracellular matrix, Liver cancer

## Abstract

Yes-associated protein (YAP) and transcriptional coactivator with PDZ-binding motif (TAZ), key effectors of the Hippo pathway, are often hyperactivated in cancer, promoting tumor progression and therapy resistance. Their oncogenic role depends on interaction with TEA domain (TEAD) transcription factors, making the TEAD-YAP/TAZ complex a promising therapeutic target. Using translational mouse models, we show here that sustained systemic depletion of YAP and TAZ (YAP/TAZ) caused severe side effects. These could be avoided through pulsed inhibition, which effectively suppressed tumor growth, even at advanced stages. We identified *Tgfb2* as a critical YAP/TAZ target gene for tumor formation and demonstrated that YAP/TAZ drove T cell exclusion via activation of tissue-remodeling genes. Consequently, YAP/TAZ inhibition enhanced immune cell infiltration. However, infiltrating T cells rapidly underwent exhaustion. Combining YAP/TAZ inhibition with immune checkpoint blockade reversed this exhaustion and sensitized resistant tumors to immunotherapy. This combination reshaped the tumor microenvironment to support immune cell infiltration and activation, representing a therapeutic strategy that maximizes antitumor immunity while minimizing toxicity.

## Abstract 2

Blocking YAP/TAZ proteins combined with immunotherapy could improve cancer treatment, offering a safer, more effective strategy to overcome therapy resistance and boost anti-tumor immunity.

## Introduction

The Yes-associated protein (YAP) and transcriptional coactivator with PDZ-binding motif (TAZ, also known as WWTR1) are central effectors of the Hippo signaling pathway, a key regulator of tissue regeneration (1–3). While transient YAP and TAZ (YAP/TAZ) activation supports regenerative responses (4), their sustained activation has been strongly implicated in malignant transformation: in diverse human cancers, YAP/TAZ become hyperactivated, contributing to tumor initiation, progression, metastasis, and therapeutic resistance (5, 6). Mechanistically, YAP/TAZ promote resistance to apoptosis (7), maintain a stem cell–like transcriptional program (8), and drive aggressive phenotypes associated with poor clinical outcomes (9).

YAP and TAZ exert their oncogenic functions predominantly through their interaction with TEA domain (TEAD) family transcription factors. Acting as transcriptional coactivators, YAP/TAZ partner with TEADs to control gene expression programs, primarily through distal enhancers (10–12). This TEAD/YAP/TAZ axis has thus emerged as a compelling target in cancer therapy (12–15). In recent years, multiple pharmacologic approaches have been explored to disrupt this interaction and inhibit downstream transcriptional output, including small molecules targeting TEAD palmitoylation or YAP/TAZ-TEAD binding interfaces (16–19).

Importantly, the deletion of YAP/TAZ in adult mice under homeostatic conditions frequently results in minimal or no adverse phenotypes, suggesting that systemic inhibition of this pathway could be well tolerated in therapeutic settings (20, 21).

Here, we leveraged translational shRNA-based mouse models to systematically evaluate both the therapeutic efficacy and potential toxicity of YAP/TAZ-targeted interventions in vivo. We find that, while continuous depletion of YAP/TAZ led to detrimental effects, transient or pulsed inhibition induced robust antitumor responses in advanced cancers. Notably, this therapeutic benefit was linked to a YAP/TAZ-dependent tissue remodeling program that indirectly modulated the tumor immune microenvironment, unveiling an unexpected mechanism of action and therapeutic opportunity.

## Results

### Ubiquitous long-term depletion of YAP/TAZ is highly detrimental.

**Figure 1:**
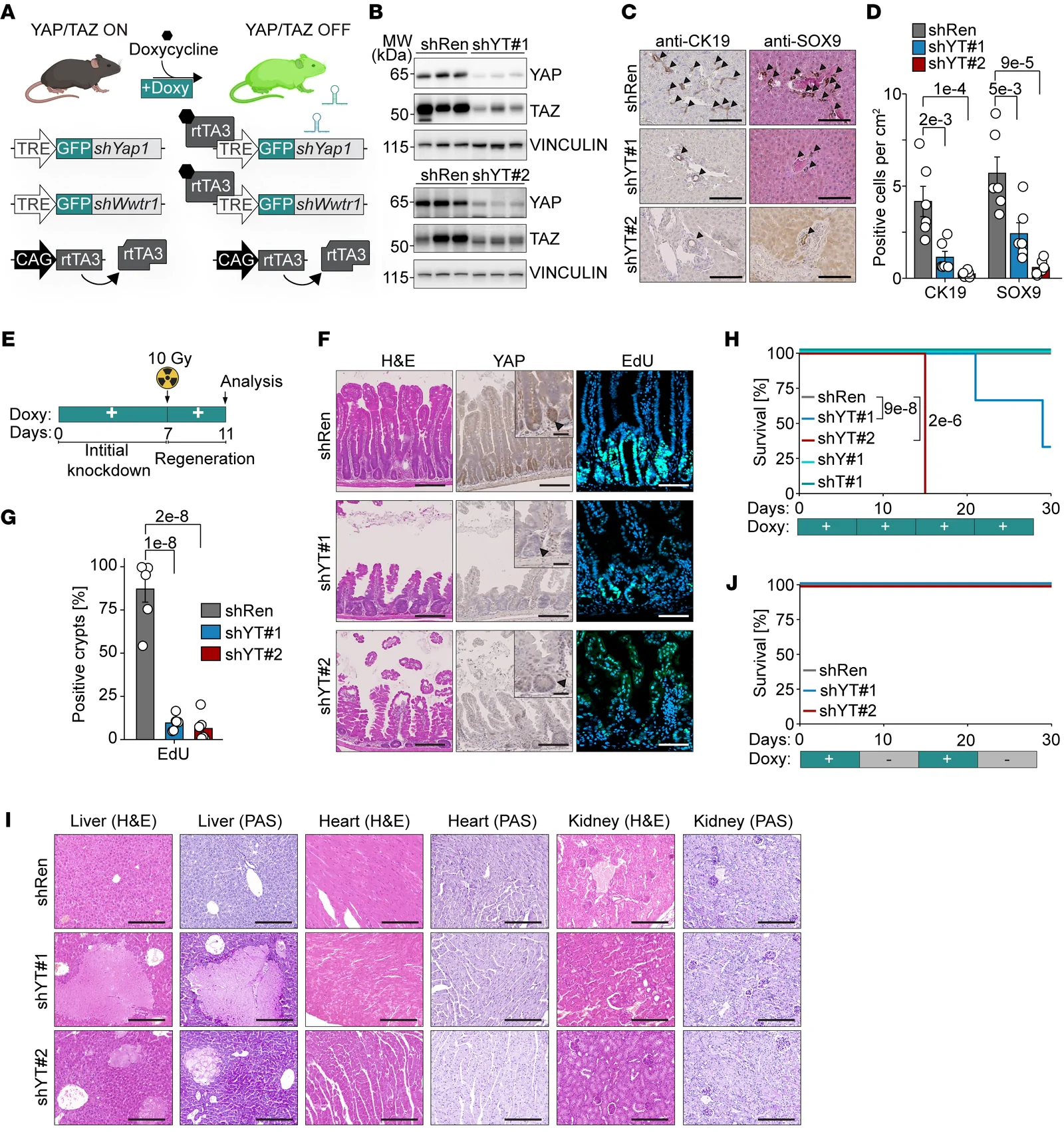
Systemic long-term depletion of YAP/TAZ is highly detrimental. (**A**) Schematic of the inducible shRNA mouse model. Ubiquitous expression of rtTA3 under the *CAG* promoter enables ubiquitous expression of doxycycline-inducible shRNAs targeting *Yap1* and *Wwtr1* (Taz). Two independent mouse lines (shYT1 and shYT2) were compared with control mice expressing an shRNA against *Renilla* (shRen). (**B**) Western blot of liver lysates confirming YAP and TAZ knockdown after 2 weeks (shYT2) or 4 weeks (shYT1) of doxycycline treatment (*n* = 3 per group). (**C** and **D**) Liver sections (**C**) stained for CK19 and Sox9; arrows indicate positive BECs (scale bars: 100 μm) and quantification (**D**) of BEC counts per liver area (*n* = 6 per group). (**E**) Experimental outline: after 7 days of doxycycline-induced (Doxy) knockdown, mice were subjected to 10 Gy irradiation, and intestinal regeneration was assessed 4 days later. (**F** and **G**) Intestinal sections (**F**) analyzed by H&E and YAP IHC, and EdU (green) with DAPI (blue). Enlarged insets highlight Paneth cells (arrows) and quantification (**G**) of EdU^+^ crypts (*n* = 6 per group). Scale bars: 200 μm (overview) and 50 μm (enlarged insets). (**H** and **J**) Kaplan-Meier survival curves under constant (**H**) or intermittent (**J**) doxycycline administration for 4 weeks. Control group: shRen (*n* = 17 [**H**], *n* = 15 [**J**]); shY1 (*n* = 9); shT1, *n* = 11; shYT1 (*n* = 6 [**H**], *n* = 7 [**J**]); shYT2 (*n* = 6 [**H**], *n* = 4 [**J**]). (**I**) Representative H&E and PAS staining of liver, heart, and kidney sections from mice with the indicated genotypes after long-term depletion of YAP/TAZ. Scale bars: 200 μm. The liver H&E image panel for shYT 1 is also shown in Supplemental Figure 4 as a representative example. Data represent the mean ± SEM. Statistical analyses: 1-way ANOVA with Tukey’s HSD post hoc test (**D** and **G**) or ranked test (**H**).

In order to model the efficacy as well as potential adverse side effects of therapies targeting YAP/TAZ, we developed a doxycycline-inducible shRNA mouse model that enables the depletion of YAP/TAZ in the entire body of a mouse (Figure 1A). An sh*Renilla* (shRen) mouse line was used as the nontargeting control as well as 2 shYAP/TAZ (shYT1 and shYT2) mouse lines, each expressing a distinct shRNA targeting *Yap* and *Taz (Wwtr1)*, respectively. Both shYT lines showed a potent knockdown of YAP and TAZ, achieving an approximately 80%–90% reduction across different tissues (Figure 1B and Supplemental Figure 1, A–E; supplemental material available online with this article; https://doi.org/10.1172/JCI201579DS1). To validate the functionality of the shRNA mouse lines, we analyzed known YAP/TAZ-dependent knockout phenotypes in the shRNA lines: loss of biliary epithelial cells (BECs) and impairment of intestinal regeneration (Figure 1, C–G). Depletion of YAP/TAZ led to a significant reduction of BECs (Figure 1, C and D, and Supplemental Figure 2, A and B) and strongly impaired intestinal regeneration after γ irradiation (Figure 1, E–G). In all our experiments shYT2 led to stronger phenotypes. These analyses thus demonstrate that the shRNA mouse model can recapitulate YAP/TAZ knockout phenotypes. Long-term depletion of YAP/TAZ (as compared with depletion of YAP or TAZ alone), was not well tolerated: most mice had to be sacrificed after 1 month because of excessive body weight loss (Figure 1H and Supplemental Figure 3, A–G). Histological analysis at the humane endpoint revealed that shYT animals had multifocal lesions across multiple organs, including hepatic inflammation, necrosis, and parenchymal degeneration; cardiomyocyte degeneration; and tubular epithelial cell damage in the kidneys (Figure 1I and Supplemental Figure 4, A–C). Thus, ubiquitous long-term YAP/TAZ depletion can lead to multiorgan defects. However, intermittent doxycycline administration could mitigate these adverse effects, enabling long-term experiments and suggesting the existence of a potential therapeutic window for YAP/TAZ inhibition (Figure 1J and Supplemental Figure 3B).

### Transient YAP/TAZ inhibition is beneficial in advanced cholangiocarcinoma.

**Figure 2:**
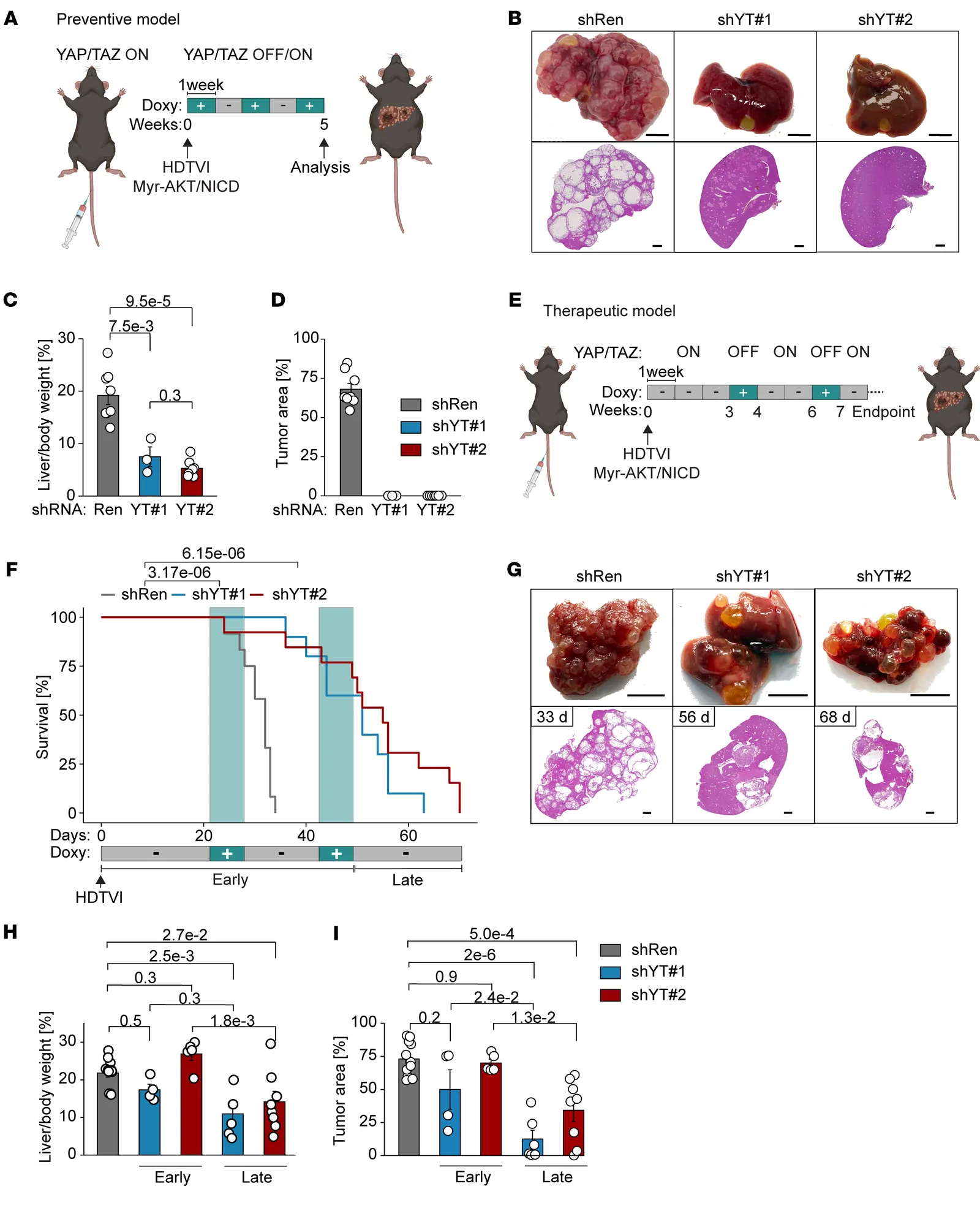
YAP/TAZ inhibition is beneficial in advanced CCA. (**A**) Preventive model: YAP/TAZ depletion was initiated in the HDTVI N-AKT CCA model via weekly intermittent doxycycline administration. Tumors were analyzed 5 weeks after HDTVI. N, notch intracellular domain. (**B**) Representative gross liver images (top) and H&E-stained liver sections (bottom) from mice of the indicated genotypes are shown. Scale bars: 1 cm (livers), 2 mm (H&E). (**C** and **D**) Quantification (**C**) of liver-to-body weight ratios and relative tumor area (**D**) (shRen, *n* = 8; shYT1, *n* = 3; shYT2, *n* = 7). (**E**) Therapeutic model: YAP/TAZ depletion was transiently performed in advanced N-AKT CCA 4 and 7 weeks after HDTVI. (**F**) Kaplan-Meier survival curves depict early (weeks 1–7) and late (weeks 8–10) survival of control mice (shRen, *n* = 12) versus YAP/TAZ-depleted mice (shYT1, *n* = 10; shYT2, *n* = 13). (**G**) Representative livers (top) and H&E-stained liver sections are shown. The respective survival rates (in days) are indicated. Scale bars: 1 cm (livers) and 2 mm (H&E). (**H** and **I**) Quantification (**I**) of liver-to-body weight ratios and relative tumor area (**J**) (shRen, *n* = 12; shYT1, *n* = 10; shYT2, *n* = 13). Data represent the mean ± SEM. Statistical analyses: 1-way ANOVA with Tukey’s HSD post hoc test (**C**, **D**, **H**, and **I**) or ranked test (**F**).

Cholangiocarcinomas (CCAs) are highly aggressive tumors with a poor prognosis for affected patients, and they show a critical dependency on high YAP/TAZ activity (22). We thus tested the applicability of ubiquitous YAP/TAZ inhibition in tumor therapy as a proof of concept in a CCA mouse model. Intermittent YAP/TAZ inhibition was sufficient to render mice tumor free in a hydrodynamic tail vein injection–induced (HDTVI-induced) CCA model utilizing a transposase-based system to express activated versions of the notch receptor (NICD) and myristoylated AKT (myr-AKT) (Figure 2, A–D). Next, we addressed how ubiquitous YAP/TAZ inhibition affects CCA growth in a setting that more closely resembles a clinical setting, in which only 2 weekly pulses of doxycycline were administered in highly advanced tumors (Figure 2E). This regimen was sufficient to nearly double the median survival of shYT animals (Figure 2F). The analysis of tumors from shYT animals that reached the endpoint criteria after the second doxycycline pulse (“late” time point) showed a significant reduction of the liver–to–body weight ratio and tumor load, demonstrating that transient YAP/TAZ inhibition was sufficient to severely reduce the tumor burden in highly advanced CCAs (Figure 2, F–I).

### Tumor cell–specific YAP/TAZ depletion recapitulates ubiquitous YAP/TAZ depletion.

**Figure 3:**
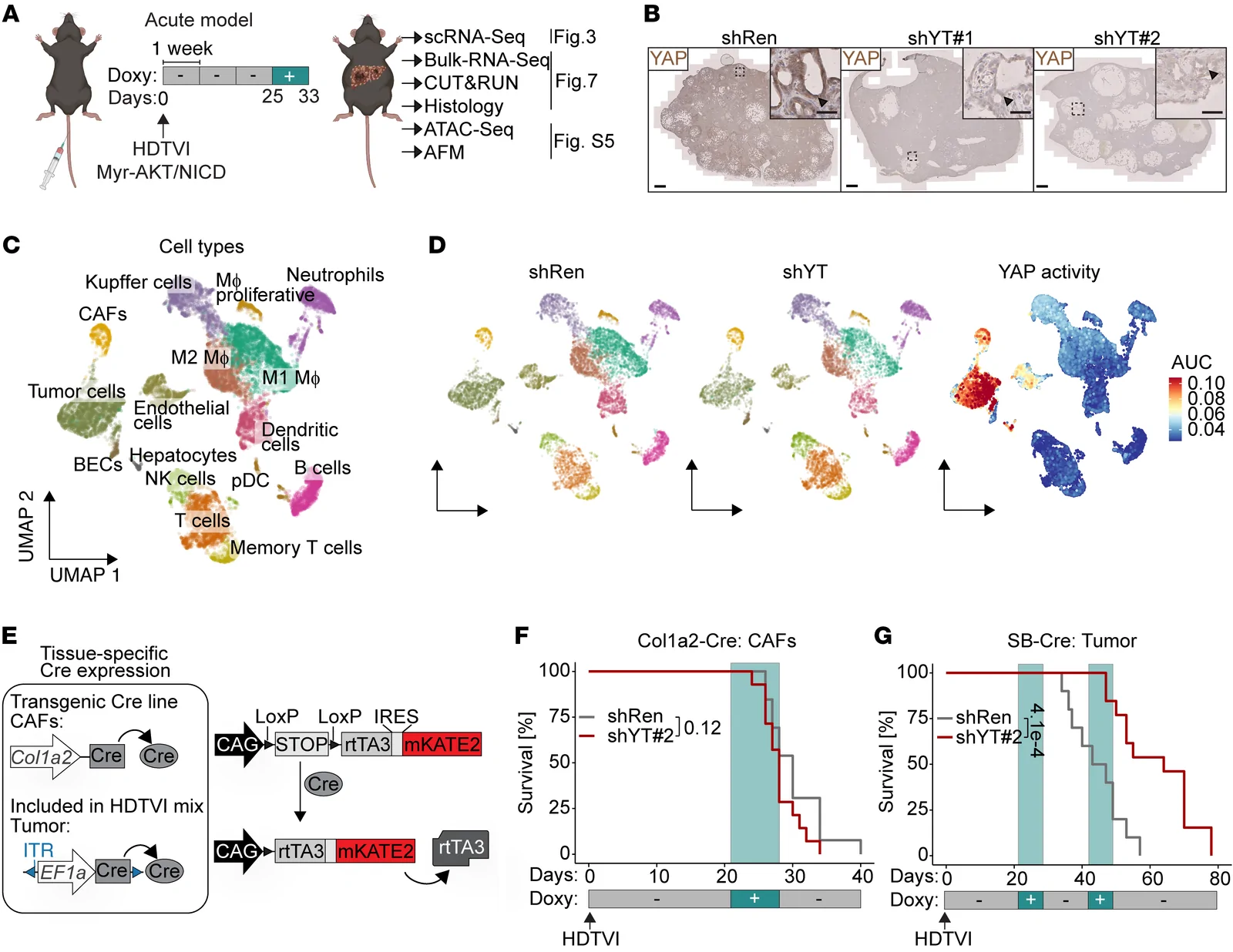
Tumor cell–specific YAP/TAZ depletion recapitulates ubiquitous YAP/TAZ depletion. (**A**) Acute depletion model: YAP/TAZ depletion was initiated at day 25 after HDTVI and analyzed at day 33 in the N-AKT CCA model. (**B**) IHC images of YAP staining (brown) in tumor-bearing livers. Arrows in the magnified view mark YAP^+^ tumor cells. Scale bars: 1 mm (overviews) and 50 μm (enlarged insets). (**C** and **D**) UMAP plots from scRNA-seq following acute YAP/TAZ depletion: (**C**) overview of cell types in the N-AKT CCA model; (**D**, left) cell composition in shRen and shYT tumors; (**D**, right) YAP activation scores indicated across all cell types, based on a conserved YAP signature (24) (shRen, *n* = 4; shYT 1, *n* = 2; shYT 2, *n* = 2). pDC, plasmacytoid DC. (**E**) Schematic of tissue-specific rtTA3 expression (Rosa26-CAG-lsl-RIK). Cre-mediated deletion of the STOP cassette enables tissue-specific shRNA expression in CAFs (Col1a2-Cre) or tumor cells (Cre via plasmid in HDTVI). (**F** and **G**) Kaplan-Meier survival curve (log-rank test) for mice with (**F**) CAF-specific (shRen, *n* = 13; shYT2, *n* = 14) or (**G**) tumor cell–specific (shRen, *n* = 10; shYT2, *n* = 13) YAP/TAZ knockdown.

Next, we aimed to gain mechanistic insight into the early events triggered by YAP/TAZ inhibition in advanced tumors. To this end, we knocked down YAP/TAZ in advanced CCAs and analyzed the acute downstream effects (Figure 3A and Supplemental Figure 5). The mice were fed doxycycline starting from day 25 after HDTVI, and their tumors were analyzed 8 days later.

Since it takes approximately 5 days for a knockdown to fully establish in vivo (23), the tumors were depleted of YAP/TAZ for approximately 3 days. Consistently, we found that YAP was barely detectable in shYT CCAs and that *Yap1* and *Taz* mRNAs were strongly downregulated in our RNA-Seq data, coinciding with downregulation of the YAP/TAZ targets (Figure 3B and Supplemental Figure 5B, and Supplemental Table 1). In the scRNA-Seq analyses (shRen: *n* = 4, shYT1: *n* = 2, shYT2: *n* = 2), we could identify the tumor cells on the basis of expression of AKT/NICD and annotate other cell types (Figure 3, C and D, and Supplemental Figure 6A). Using a highly conserved YAP/TAZ signature (24) to assess overall activity, we observed that tumor cells and cancer-associated fibroblasts (CAFs) exhibited the highest YAP/TAZ activity (Figure 3D). These cell types also showed the most pronounced changes in YAP/TAZ target gene expression following YAP/TAZ depletion (Supplemental Figure 6, B and C). Selective YAP/TAZ depletion in either CAFs or tumor cells (Figure 3E), achieved via a Cre-inducible rtTA3 allele, revealed that targeting YAP/TAZ in tumor cells conferred a substantial survival advantage, whereas depletion in CAFs did not have a beneficial effect (Figure 3, F and G).

### Acute YAP/TAZ depletion in advanced CCAs leads to T cell infiltration and activation.

**Figure 4:**
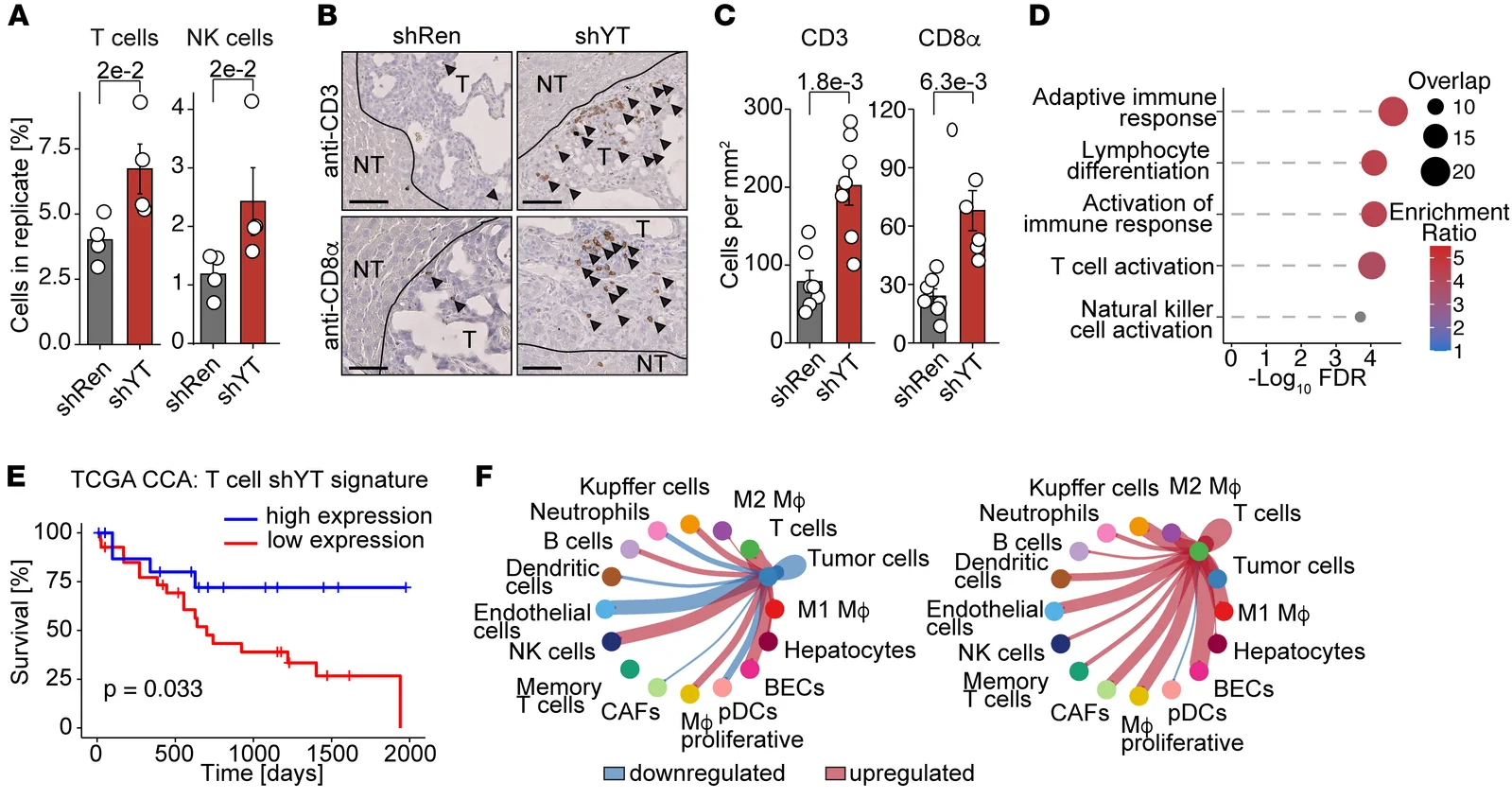
Acute YAP/TAZ depletion in advanced CCA promotes T cell infiltration and activation. (**A**) Proportion of T cells (left) and NK cells (right), shown as the percentage of total cells detected by scRNA-seq, following acute YAP/TAZ depletion (shRen, *n* = 4; shYT1, *n* = 2; shYT2, *n* = 2). (**B** and **C**) CD3 and CD8a immunostaining (**B**) in CCAs after acute YAP/TAZ depletion, with quantification (**C**) (shRen, *n* = 7; shYT1, *n* = 2; shYT2, *n* = 5). Arrowheads indicate positive cells. Scale bars: 100 μm. (**D**) Dot plot showing enriched pathways in YAP/TAZ-depleted T cells. (**E**) Kaplan-Meier survival curve for patients with CCA (TCGA dataset) stratified by high versus low expression of a YAP/TAZ-dependent T cell activation signature. (**F**) Circle plots showing the predicted ligand-receptor interactions of tumor cells (left) and T cells (right) with other cell types, inferred by CellChat. Line thickness indicates interaction strength; red and blue lines represent upregulated and downregulated interactions, respectively. MΦ, macrophages. Data represent the mean ± SEM. Statistical analyses: Welch’s *t* test with Benjamini-Hochberg correction (**A** and **C**) and log-rank test (**E**).

T and NK cells, which increased approximately 2-fold in shYT tumors, showed the most drastic relative increase of all cells within tumors in shYT animals (Figure 4A and Supplemental Figure 7A). In addition, clusters of intratumoral T cells and CD8^+^ cytotoxic T cells could only be observed in shYT tumors (Figure 4, B and C). T cells in shRen tumors could only be found sparsely at the tumor margin, indicating that high YAP/TAZ played an active role in T cell exclusion. Differential gene expression analyses of the scRNA-Seq data set within the T cell cluster revealed potent transcriptional responses indicative of T cell activation (Figure 4D), arguing that YAP/TAZ depletion allows (a) T cells to enter the tumor and (b) to become activated within in the tumor. An shYT T cell signature derived from the scRNA-Seq data set was highly predictive in a CCA dataset from TCGA, demonstrating that YAP/TAZ depletion can have a clinically important effect on the activities of intratumoral immune cells (Figure 4E and Supplemental Table 2). An intercellular communication network analysis using CellChat (25) revealed that (a) tumor cells showed enhanced interactions with T and NK cells, and (b) T cells were predicted to increase their engagement with virtually all cell types (Figure 4F and Supplemental Figure 7B). Importantly, and in contrast to findings in other cancer types (26), YAP/TAZ knockdown did not alter Cd274 (programmed cell death ligand 1 [PD-L1]) expression in CCA tumor cells (Supplemental Figure 7C).

### The therapeutic effect of YAP/TAZ depletion depends on CD8^+^ cytotoxic T cells.

**Figure 5:**
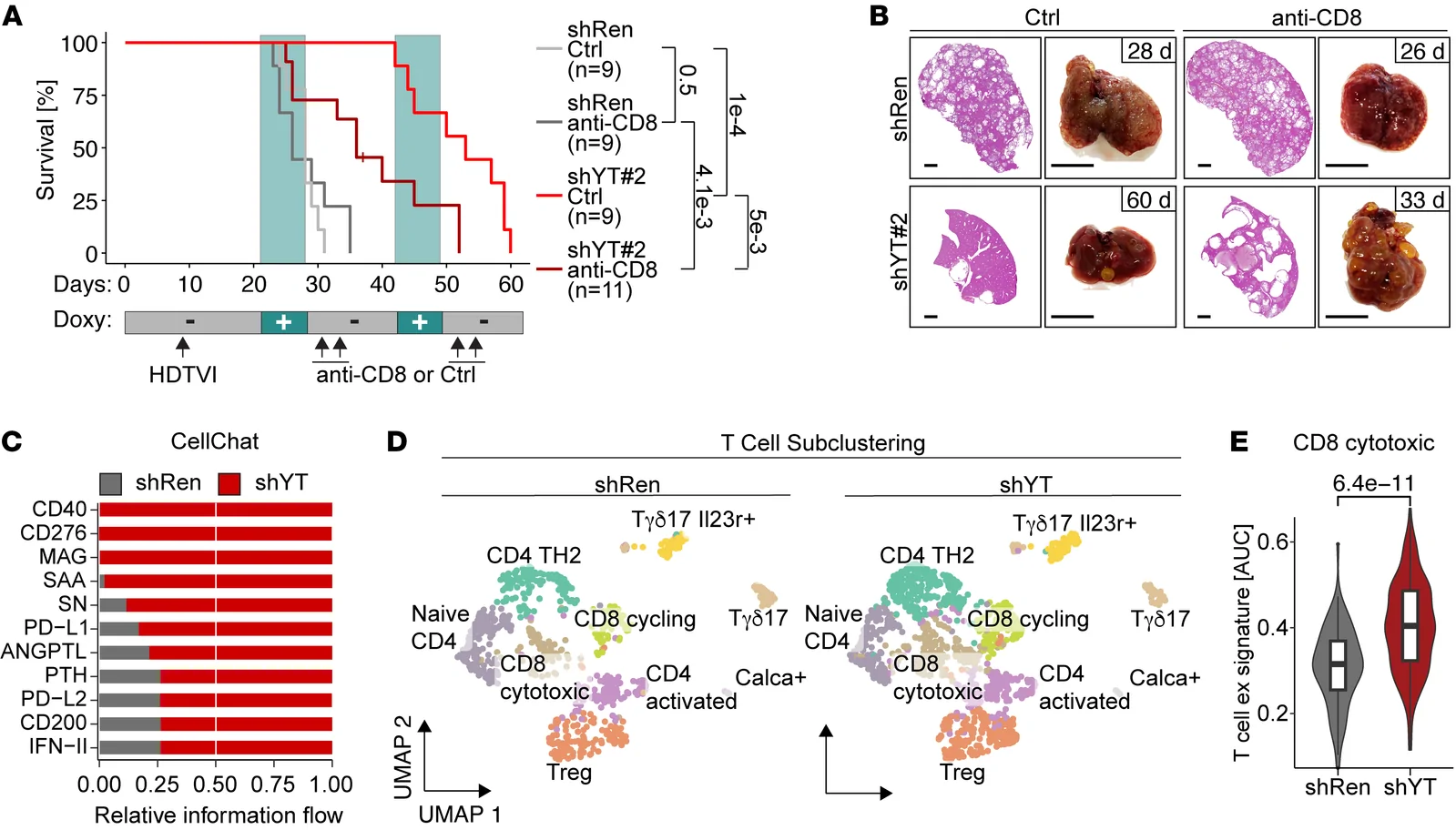
The therapeutic effect of YAP/TAZ depletion depends on CD8^+^ cytotoxic T cells. (**A** and **B**) Kaplan-Meier survival curves for shRen and shYT2 CCA-bearing mice with or without CD8^+^ T cell depletion (shRen Ctrl, *n* = 9; shRen anti-CD8, *n* = 9; shYT2 Ctrl, *n* = 9; shYT2 anti-CD8, *n* = 11). Representative livers and H&E-stained liver sections are shown with the respective survival rates (in days). Scale bars: 1 cm (gross images) and 1 mm (H&E). (**C**) CellChat analysis showing significantly upregulated ligand-receptor interactions upon YAP/TAZ depletion. The *x*-axis indicates relative information flow, a quantitative measure of overall signaling activity. (**D**) UMAP plots illustrating reclustering of the T cell compartment from scRNA-seq data (Figure 3). (**E**) Expression of a T cell exhaustion signature in CD8^+^ cytotoxic T cells from shRen and shYT2 tumors. Statistical analyses: log-rank test (**A**) and Wilcoxon-rank test (**E**). Box plot: center line reflects the median; box limits: Q1 and Q3; whiskers: data points within 1.5 times the IQR.

To test the contribution of CD8^+^ cytotoxic T cells to the survival benefit in the therapeutic setting of shYT animals, we immunodepleted CD8^+^ cytotoxic T cells during the 2 weekly doxycycline pulses (Figure 5A and Supplemental Figure 8). The depletion of CD8^+^ cytotoxic T cells had no noticeable effect on the shRen control group, indicative of blunted immunosurveillance in the CCA model. In shYT2 animals, however, the depletion of CD8^+^ T cells significantly decreased the survival advantage, indicating that YAP/TAZ depletion specifically activated CD8^+^ cytotoxic T cells, contributing to the observed survival benefit (Figure 5, A and B).

Further CellChat analyses revealed that the most significantly altered pathways in shYT animals were predominantly immune checkpoint related — such as pathways for PD-L1, PD-L2, and CD276, which became almost exclusively activated in this context (Figure 5C). Subclustering of T cells corroborated the observation that shYT tumors had a strong increase in exhaustion signatures (Figure 5, D and E, and Supplemental Table 3). YAP/TAZ can induce PD-L1 expression on tumors (27, 28). However, the observed survival benefit in YAP/TAZ-depleted mice was most likely not attributable to this mechanism, as PD-L1 expression remained unchanged in both tumor cells and the tumor microenvironment (TME) following YAP/TAZ depletion (Supplemental Figure 9A). A plausible explanation for the increased T cell exhaustion signatures detected by CellChat is that YAP/TAZ depletion enables CD8^+^ T cells to more efficiently infiltrate the tumor, where they encounter a highly immunosuppressive microenvironment characterized by elevated immunosuppressive cytokines, such as IL-10, and widespread expression of immunosuppressive molecules, including PD-L1, on tumor-associated macrophages and other cell types (Supplemental Figure 9, B and C).

### YAP/TAZ depletion sensitizes CCAs toward immune checkpoint inhibitors.

**Figure 6:**
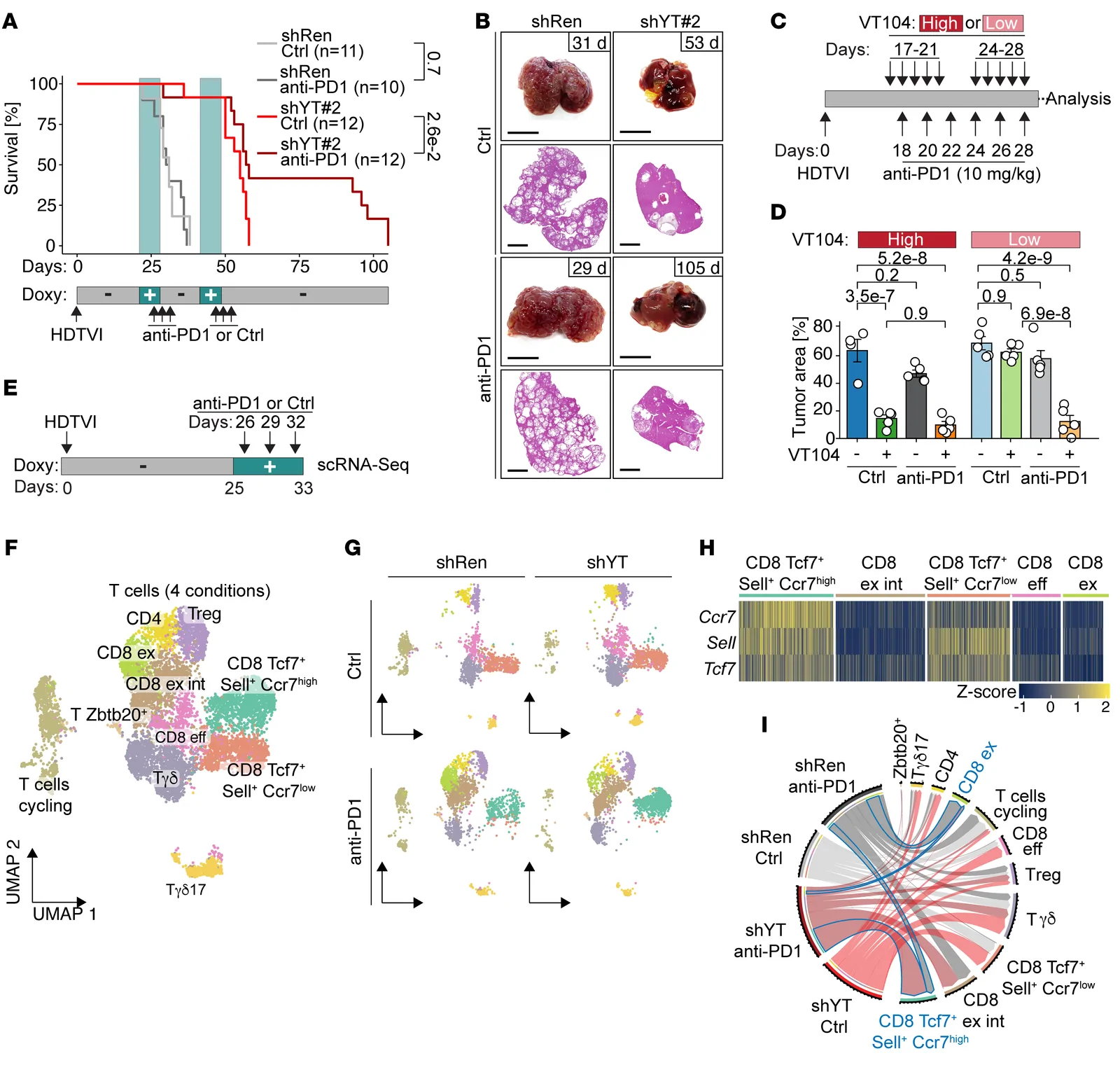
YAP/TAZ depletion sensitizes CCAs toward ICB. (**A**) Kaplan-Meier survival curves for shRen and shYT2 animals treated with or without anti–PD-1 (shRen control [shRen Ctrl], *n* = 11; shRen anti–PD-1, *n* = 10; shYT2 Ctrl, *n* = 12; shYT2 anti–PD-1, *n* = 12). (**B**) Representative livers and H&E-stained liver sections are shown with the respective survival rates (in days). Scale bars: 1 cm (gross images) and 2 mm (H&E). (**C**) Schematic of combination therapy with high (10 mg/kg) or low (1 mg/kg) doses of VT104 and anti–PD-1 in N-AKT–driven CCA. (**D**) Tumor burden quantified in each group (*n* = 5; Ctrl [high-dose VT104], *n* = 4). (**E**) Schematic of acute YAP/TAZ depletion combined with anti–PD-1, followed by scRNA-seq analysis. (**F** and **G**) UMAP plots showing T cell clusters across all conditions (**F**) or stratified by treatment groups (**G**) (*n* = 4 per group). (**H**) Heatmap of marker genes used for T cell subtype annotation of Tcf7^+^ Sell^+^ T cells. (**I**) Chord diagram showing the relative abundance of T cell subtypes across treatment groups. The most prominent shifts in T cell populations are highlighted in blue. Data represent the mean ± SEM. Statistical analyses: 1-way ANOVA with Tukey’s HSD post hoc test (**D**) and log-rank test (**A**).

These findings suggest that, although YAP/TAZ depletion triggers some level of T cell activation, immune checkpoints continue to restrain their full response, presenting an additional barrier to immune engagement. In the clinic, CCAs respond very poorly to immune checkpoint blockade (ICB) with agents such as anti–programmed death 1 (anti–PD-1) antibodies, which is largely attributed to the desmoplastic nature of CCAs as well as their immunosuppressive TME (29). From our previous analyses, we hypothesized that CD8^+^ cytotoxic T cells infiltrating the tumor following YAP/TAZ depletion are rapidly suppressed and exhausted due to immune checkpoint activation. On the basis of this hypothesis, we posited that ICB drugs should show a synergistic efficacy with YAP/TAZ depletion. To investigate this, we analyzed the survival of shRen and shYT2 animals that were administered an anti–PD-1 antibody targeting murine PD-1. shRen animals did not show any measurable response to anti–PD-1 treatment, whereas shYT2 animals demonstrated a strong survival benefit with a high percentage of animals having a doubled survival rate and a reduced tumor load (Figure 6, A and B).

These findings could be recapitulated with the TEAD inhibitor VT104 (16): at a high concentration (10 mg/kg), VT104 alone markedly reduced tumor burden, potentially reaching a therapeutic ceiling beyond which additional benefit from anti–PD-1 treatment was not readily detectable. In contrast, at a lower VT104 concentration (1 mg/kg), anti–PD-1 treatment strongly synergized with VT104 to reduce tumor burden. (Figure 6, C and D, and Supplemental Figure 10). To understand how YAP/TAZ depletion sensitizes tumors to ICB, we performed scRNA-Seq after YAP/TAZ depletion in combination with anti–PD-1 (Figure 6E). As expected, anti–PD-1 led to a major shift in the T cell populations (Figure 6, F and G). Within the T cell population, we identified distinct subfractions of CD8^+^ cytotoxic T cells exhibiting a progressive increase in exhaustion signatures (Supplemental Figure 11 and Supplemental Tables 3 and 4), transitioning from CD8 effector (eff) to CD8 exhausted intermediate (ex int), and ultimately to fully exhausted (ex) CD8^+^ T cells. Tumors in shYT animals treated with anti–PD-1 antibodies exhibited a marked increase in CD8 memory-like CD8^+^Tcf7^+^Sell^+^Ccr7^hi^ cells (Tcf7, T cell factor 7; Sell, selectin L/CD62L; Ccr7, C-C motif chemokine receptor 7) and a lower fraction of CD8 ex cells compared with shRen animals that also received anti–PD-1 therapy (Figure 6, G–I). The fraction of intratumoral CD8 memory-like cells is a strong predictor for an efficient response to ICB (30), arguing that YAP/TAZ facilitates the ICB response via this critical fraction of immune cells.

### YAP/TAZ drive remodeling of the extracellular matrix.

**Figure 7:**
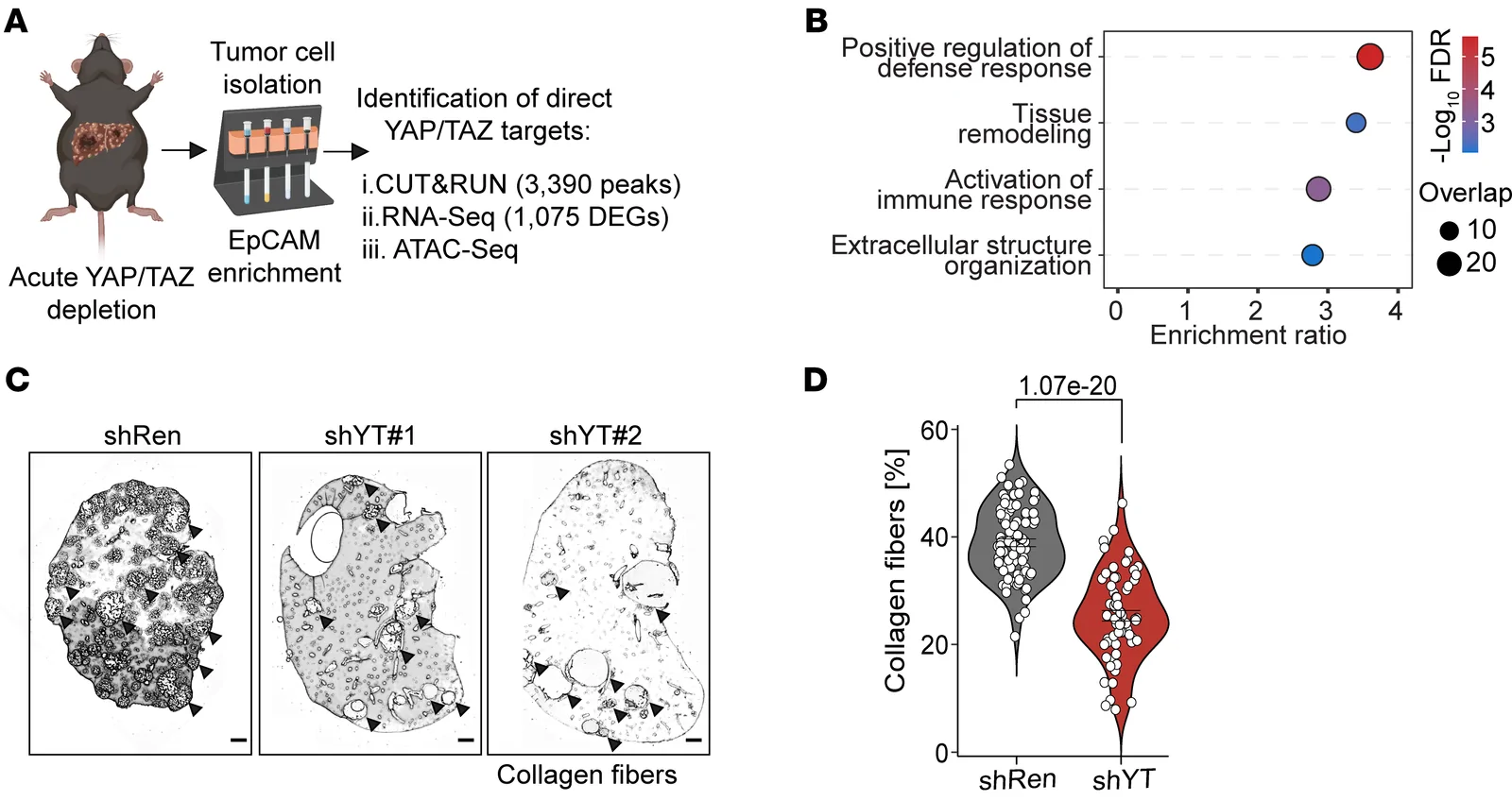
YAP/TAZ drive an extracellular matrix remodeling program. (**A**) Schematic outlining the identification of direct YAP/TAZ target genes in CCA cells. (**B**) Dot plot for pathway enrichment analysis, highlighting immune activation and extracellular matrix organization pathways upregulated upon acute YAP/TAZ depletion. (**C** and **D**) Representative Weka-segmented images (**C**) of Picrosirius red–stained liver sections, highlighting collagen fibers (black) and background tissue (white) in tumors with acute YAP/TAZ depletion (shYT1, *n* = 2; shYT2, *n* = 5) or control tissue (shRen, *n* = 6) and quantification (**D**) of collagen content. Statistical analyses: Welch’s *t* test with Benjamini-Hochberg correction (**D**). Scale bars: 1 mm.

Given our findings, it is evident that TEAD-YAP/TAZ drive a transcriptional program that helps CCAs to grow aggressively and to escape immunosurveillance, e.g., by driving T cell exclusion. By comparing transcriptional profiles of tumor cells with their nonmalignant counterparts — BECs and hepatocytes — we found that YAP/TAZ activated a tumor-specific transcriptional program that was absent in nonmalignant cells (Supplemental Figure 12A and Supplemental Table 5). To identify the primary drivers of this oncogenic TEAD-YAP/TAZ program, we performed CUT&RUN analysis for TEAD1 and YAP in EpCAM-enriched tumor cells (Supplemental Figure 12, B and C). These data were integrated with bulk RNA-seq and ATAC-seq datasets generated from the same purified cells (Figure 7A). TEAD1 CUT&RUN experiments revealed 3,390 high-confidence peaks (*q* < 1 × 10^–4^) that were strongly enriched for TEAD motifs (Supplemental Figure 12B). Since TEAD proteins preferentially bind to enhancer regions (31), directly linking peaks to gene regulation is challenging. To address this, we used the ATAC-Seq data from acutely depleted CCA tumors to assess chromatin accessibility and validate the functional relevance of TEAD1 binding sites (Supplemental Figure 5, C–E).

Notably, TEAD motifs were highly enriched in regions that became less accessible following YAP/TAZ depletion (Supplemental Figure 5E). On the basis of the cumulative distribution frequency (CDF) of TEAD1 peaks, we inferred that genes located within 10^7^ bp of a TEAD1 peak likely represent the majority of direct targets (Supplemental Figure 12C). In the bulk RNA-Seq dataset of purified tumor cells, gene ontology (GO) terms associated with tissue remodeling were significantly enriched among the downregulated genes, which was accompanied by a marked reduction in intratumoral collagen and loss of the desmoplastic nature (Figure 7, B–D, and Supplemental Figure 5, F and G). Since YAP/TAZ activity is closely linked to mechanotransduction (32), we assessed tissue elasticity (Young’s modulus) using an atomic force microscope (AFM) by measuring force-distance curves on the sectioned tissues. The differences in the Young’s modulus between normal, shRen, and shYT tissues indicated a decrease in tissue elasticity. Unexpectedly, YAP/TAZ depletion resulted in a mild increase in tissue stiffness (Supplemental Figure 5H).

### Tgfb2 is a critical target of the oncogenic program driven by YAP/TAZ.

**Figure 8:**
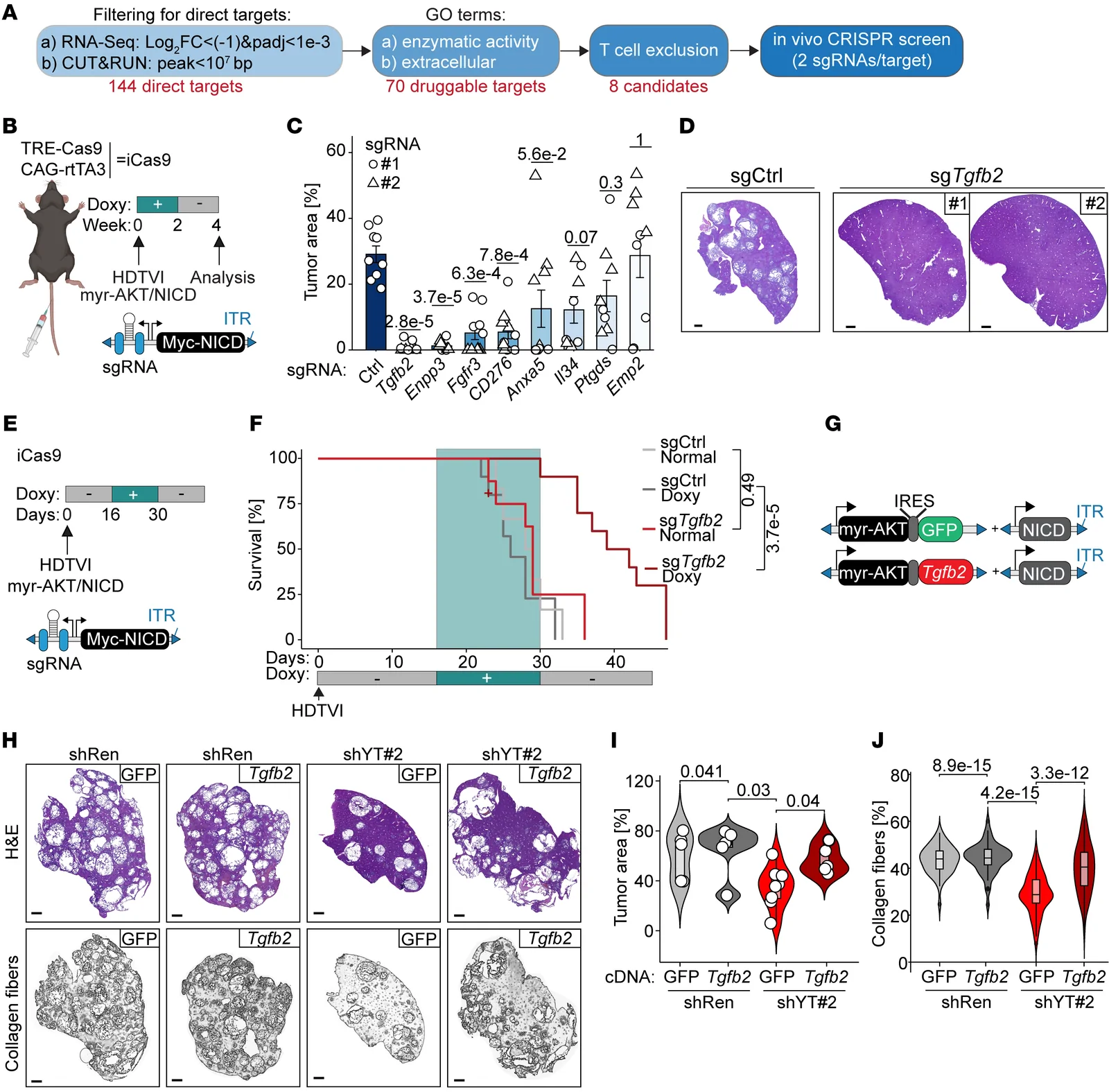
*Tgfb2* is a critical target of the oncogenic program driven by YAP/TAZ. (**A**) Schematic overview of the filtering pipeline used to define YAP/TAZ targets for the in vivo CRISPR screen. (**B**) Schematic of an inducible Cas9 mouse model combined with HDTVI of sgRNA-expressing plasmids to evaluate the functional role of candidate YAP/TAZ targets in N-AKT–driven CCA growth. (**C** and **D**) Representative H&E-stained sections (**D**) of the indicated gene knockouts with quantification (**C**) of the tumor area. Scale bars: 1 mm. (*n* = 5, per group; *n* = 4, sgIl34 no. 1, no. 2; *n* = 9, sgCtrl). (**E**) Schematic of the *Tgfb2* knockout in advanced CCA tumors. (**F**) Kaplan-Meier survival curves for sgCtrl and sg*Tgfb2* animals treated with or without doxycycline-supplemented food (Normal) (sgCtrl Normal, *n* = 6; sgCtrl Doxy, *n* = 10; sg*Tgfb2* Normal, *n* = 8; sg *Tgfb2* Doxy, *n* = 10). (**G**) Schematic of the constructs used in **H**–**J**. (**H**–**J**) Representative images of H&E-stained (top) and Picrosirius red–stained (bottom) liver sections. Weka segmentation was used to highlight collagen fibers (black). Tumors were subjected to acute YAP/TAZ depletion for 8 days doxycycline administration. TGFB2 was reexpressed in these tumors, while GFP expression served as a control (shRen GFP, *n* = 5; shRen TGFB2, *n* = 5; shYT2 GFP, *n* = 8; shYT2 TGFB2, *n* = 5). Tumor area (**I**) and collagen content (**J**) were quantified. Scale bars: 1 mm. Data represent the mean ± SEM. Box plots show the median (center line), IQR (box), and whiskers extending to 1.5 times the IQR. Data points beyond this range are shown as outliers. Statistical analyses: 1-way ANOVA with Tukey’s HSD post hoc test (**C** and **J**); log-rank test (**F**); and Kruskal-Wallis test followed by Dunn’s post hoc test with Benjamini-Hochberg correction (**I**).

These findings suggested that YAP/TAZ regulate a critical set of direct targets involved in tissue remodeling, ultimately contributing to T cell exclusion. Therefore, we compiled a list of 144 direct TEAD1 targets that are responsive to YAP/TAZ depletion (Supplemental Figure 12D and Supplemental Table 6). Further filtering using GO terms for “enzymatic activity” and “extracellular” identified 70 potentially druggable candidates from which we selected 8 known to be involved in T cell exclusion (Figure 8A). These genes were knocked out after HDTVI in vivo, using a doxycycline-inducible Cas9 mouse line (iCas9) (Figure 8B). The most prominent hit was *Tgfb2*, as its knockout was as effective as targeting the primary oncogenic driver myr-AKT (Figure 8, C and D, and Supplemental Figure 13, B–E). To analyze which cell types contribute to *Tgfb2* expression in CCA, we analyzed *Tgfb2* expression in our scRNA-seq dataset. We found that *Tgfb2* was almost exclusively expressed in tumor cells, indicating that these were the only source of *Tgfb2* in CCA (Supplemental Figure 14A). Upon acute YAP/TAZ depletion, *Tgfb2* expression was markedly reduced within the tumor cell cluster, supporting the notion that *Tgfb2* is a bona fide downstream target of YAP/TAZ (Supplemental Figure 14B). Of note, *TGFB2* expression was significantly elevated in samples from patients with CCA compared with expression in normal liver tissue, indicating tumor-associated upregulation (Supplemental Figure 14C), and *TGFB2* expression shows a strong positive correlation with a conserved YAP transcriptional signature (24) across 32 cancer types within the Pan-Cancer Atlas, highlighting *TGFB2* as a conserved YAP target gene across diverse malignancies (Supplemental Figure 14, D and E).

Since *Tgfb2* emerged as the top hit in our in vivo CRISPR screen using a preventive model, we next investigated whether acute *Tgfb2* knockout could also confer therapeutic benefit in the context of established tumors, analogous to YAP/TAZ depletion. To this end, Cas9 expression was induced at day 18 after HDTVI, and mouse survival was monitored (Figure 8, E and F). Here, we observed that acute *Tgfb2* deletion could substantially extend the survival of the animals (Figure 8F), which resembled the tumor-specific depletion of YAP/TAZ (Figure 4G). Next, we sought to determine whether exogenous *Tgfb2* expression could restore oncogenicity in YAP/TAZ-depleted animals (Figure 8G). In support of the notion that *Tgfb2* is a critical downstream target of the YAP/TAZ oncogenic program, exogenous TGF-β2 expression in acutely YAP/TAZ-depleted tumors was sufficient to rescue tumor growth to levels comparable to those in shRen control tumors expressing GFP as well as the desmoplastic nature of these tumors (Figure 8, H–J).

### YAP/TAZ drive Tgfb2 expression from a distant enhancer.

**Figure 9:**
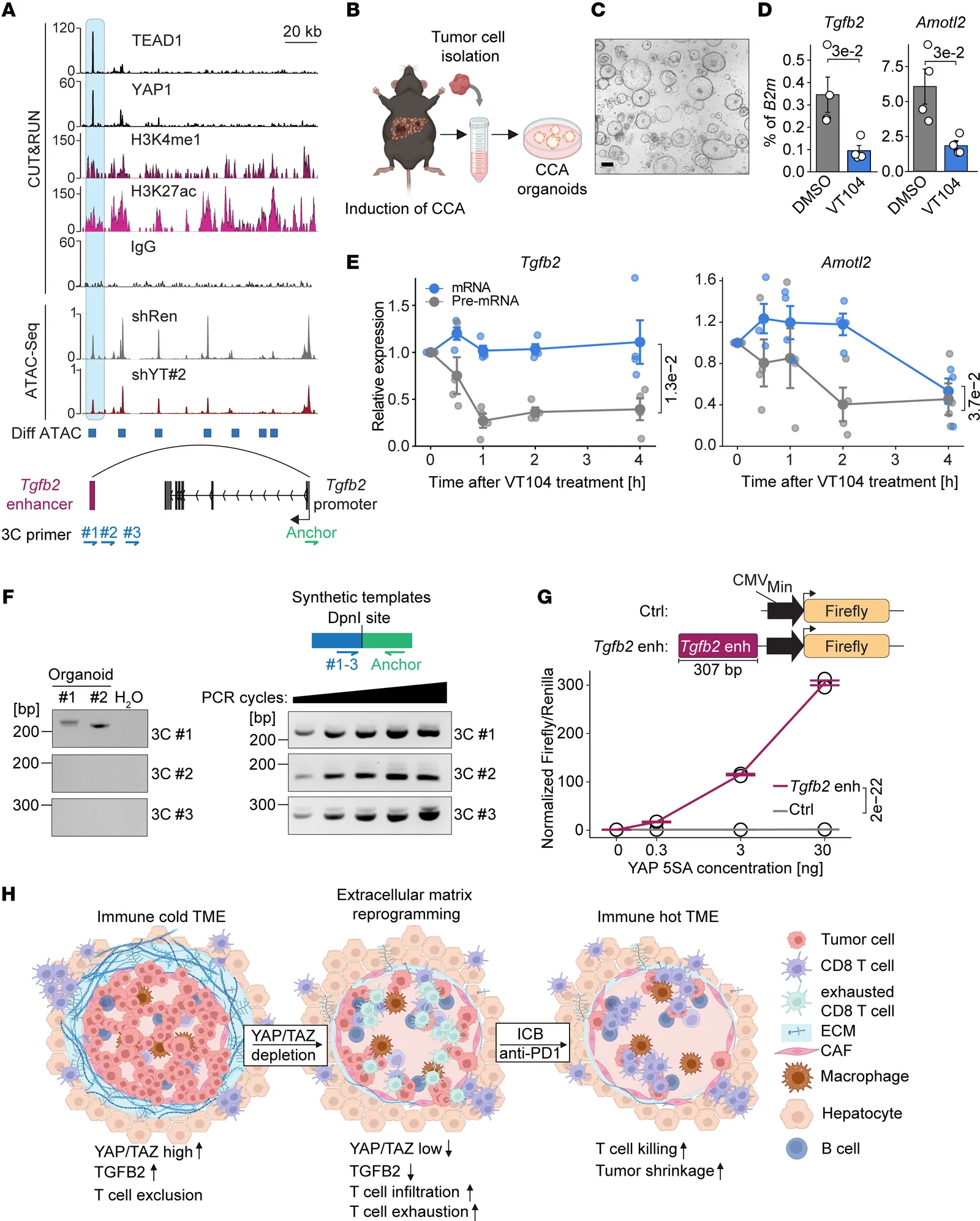
YAP/TAZ drive *Tgfb2* expression from a distant enhancer. (**A**) Genome browser tracks at the *Tgfb2* locus showing CUT&RUN profiles for TEAD1, YAP1, H3K4me1, H3K27ac, and IgG, together with ATAC-seq data, in control (shRen) and YAP/TAZ-depleted (shYT2) tumor cells. “Diff ATAC” indicates differential ATAC-seq peaks that are reduced in shYT2 tumor cells. To assess enhancer-promoter interactions at the *Tgfb2* locus, 3C primers (nos. 1–3) were used in combination with an anchor primer located at the promoter. (**B**) Schematic illustrating the isolation of organoids from CCA-bearing livers. (**C**) Representative bright-field image of CCA organoids. Scale bar: 200 μm. (**D**) Bar plot showing relative expression of *Tgfb2* and *Amotl2* in CCA organoids following 24 hours of treatment with the TEAD inhibitor VT104 (DMSO, *n* = 4; VT104, *n* = 4). (**E**) Relative expression of Tgfb2 and Amotl2 mRNA and pre-mRNA at 0, 0.5, 1, 2, and 4 hours after VT104 treatment (DMSO, *n* = 4; VT104, *n* = 4). (**F**) Chromosome conformation capture (3C) assay demonstrating interaction between the promoter anchor site and the 3C #1 region (left). Synthetic DNA templates were used as positive controls to validate primer specificity (right). (**G**) Luciferase reporter assay showing YAP-dependent activity of the *Tgfb2* enhancer (*Tgfb2* enh) compared to a control construct (Ctrl) containing only a minimal CMV promoter. *Renilla* luciferase signal was used for normalization (*n* = 3). (**H**) Proposed 2-hit model for the synergy between YAP/TAZ depletion and immune checkpoint blockade (ICB): YAP/TAZ depletion remodels the extracellular matrix, facilitating T cell infiltration and promoting T cell exhaustion. ICB reverses this exhausted state, thereby rendering the CCA TME responsive to immunotherapy. Data represent the mean ± SEM. Statistical analyses: paired Welch’s *t* test with Benjamini-Hochberg correction (**D**), 2-way ANOVA (**E** and **G**).

On the basis of our ATAC-Seq and CUT&RUN data (Figure 9A), we could identify 1 putative enhancer region that was located approximately 140 kb downstream of the *Tgfb2* transcriptional start site (TSS). In order to have a system that is compatible with more acute YAP/TAZ depletion, we generated tumor organoids from mouse CCAs (Figure 9, B and C). VT104 treatment for 24 hours substantially reduced the mRNA expression of *Tgfb2* and the well-established YAP/TAZ target gene *Amotl2* (Figure 9D). To assess the acute transcriptional effect of VT104, we additionally measured effects on the pre-mRNA of *Tgfb2* and *Amotl2* (Figure 9E), using primer pairs spanning exon-intron boundaries, while mature mRNA levels were simultaneously assessed using exon-spanning primers. *Tgfb2* pre-mRNA was already potently reduced after 1 hour of VT104 treatment, a regulation that was even faster than that of *Amotl2* (Figure 9E). To test whether the putative *Tgfb2* enhancer physically contacts the *Tgfb2* promoter, we performed chromosome conformation capture (3C) analyses, probing 3 downstream sites using specific primers (3C1, 3C2, 3C3), together with an anchor oligonucleotide positioned at the *Tgfb2* TSS (Figure 9A). Primer 3C1 — located directly within the putative enhancer region — yielded a positive PCR signal, whereas 3C2 and 3C3 produced no detectable band (Figure 9F). The absence of signal with 3C2 and 3C3 was not attributable to poor primer performance, as these primers could amplify synthetic oligonucleotide templates with efficiency comparable to that of 3C1. Finally, we tested whether a 307 bp fragment of the putative *Tgfb2* enhancer could transactivate a reporter driven by a minimal CMV promoter (Figure 9G). Expression of a constitutively active YAP5SA mutant induced reporter activity up to approximately 300-fold, while a control reporter lacking the enhancer fragment was completely unresponsive (Figure 9G). Collectively, these findings establish *Tgfb2* as a direct transcriptional target of YAP/TAZ, regulated through a distal enhancer element located approximately 140 kb downstream of the *Tgfb2* TSS. Functionally, acute *Tgfb2* deletion phenocopied YAP/TAZ depletion in vivo, and restoring TGFB2 expression in YAP/TAZ-depleted tumors was sufficient to rescue tumor growth, firmly positioning TGF-β2 as a critical effector of the YAP/TAZ oncogenic program in CCA.

## Discussion

Here, we present a study utilizing a translational mouse model that mimics therapies targeting YAP/TAZ in cancer. Our findings demonstrate that ubiquitous long-term YAP/TAZ inhibition caused severe side effects, e.g., by affecting vital organs like the kidney, heart, and liver. Strikingly, the first phase I/II clinical trial of a TEAD inhibitor (VT3989) similarly reported that continuous dosing was associated with adverse effects such as albuminuria, necessitating a switch to an intermittent dosing regimen (33). The exact origin of these lesions in our shRNA mice is currently unclear, but YAP/TAZ expression is required in cardiomyocytes of the postnatal heart (34) or in bile duct cells of adult mice (35). While YAP/TAZ depletion in this specific cell type can potentially explain at least some of the phenotypes, we cannot rule out that other cell types are also affected by decreased expression of YAP/TAZ. Previous scRNA-Seq studies demonstrated that YAP/TAZ show the highest activity in fibroblasts and endothelial cells (36), raising the possibility that YAP/TAZ depletion can potentially exacerbate the phenotypes we observed here.

These findings demonstrate that YAP/TAZ play a role in tissue homeostasis and are not exclusively required for regeneration, as shown for several tissues such as the intestine (20, 37).

Consequently, the side effects caused by therapeutic strategies targeting the oncogenic TEAD-YAP/TAZ complex over a prolonged time period are probably more severe than previously anticipated. However, pulsed inhibition preserved antitumor efficacy while minimizing toxicity. This suggests that — although not without consequence — YAP/TAZ inhibition has a broader therapeutic window. Of note, transient YAP/TAZ inhibition is effective in advanced tumors, suggesting that this approach could potentially be effective in the clinic when targeting tumors in later stages of growth. Alternatively, tumor types such as liver cancer may benefit from more targeted delivery approaches — for example, via nanoparticle-based systems — although this technology remains technically challenging.

Although in our model we depleted YAP/TAZ in all cell types of the tumor — including the TME — our scRNA-Seq data suggest that the tumor cells were the cells that had the strongest response to YAP/TAZ depletion, potentially because these cells were addicted to high YAP/TAZ expression. Consistently, tumor-specific YAP/TAZ depletion also conferred a strong survival benefit, similar to the ubiquitous model. The tumor cell–specific gene expression program that is driven by YAP/TAZ is strongly enriched for genes involved in tissue remodeling. The inhibition of this transcriptional program leads to a potent remodeling of the TME. CCAs have a highly desmoplastic stroma (38). This property serves as a physical barrier for T cells in several tumor entities (39, 40) and is predictive of a weak response to ICB. Our findings suggest that YAP/TAZ-driven tissue remodeling indirectly promoted T cell exclusion, thereby impairing effective tumor cell elimination by CD8^+^ cytotoxic T cells. Supporting this notion, our in vivo CRISPR screen revealed critical tumor cell–specific YAP/TAZ target genes that regulate tissue remodeling and are indispensable for CCA growth. Of note, the most prominent hit in this analysis was *Tgfb2* and high expression of a TGF-β signature has previously been linked to T cell exclusion and attenuated the ICB response in urothelial cancer (39). Unexpectedly, we found that CD8^+^ cytotoxic T cells showed signs of exhaustion shortly after infiltrating YAP/TAZ-depleted tumors, a finding that is supported by recent reports indicating that exhaustion can occur rapidly following immune activation (41). We show that YAP/TAZ depletion enabled CD8^+^ cytotoxic T cells to efficiently infiltrate the tumor for the first time, where they encountered the highly immunosuppressive TME that T cells in control animals were never exposed to. We therefore propose that YAP/TAZ depletion indirectly drives exhaustion of infiltrating CD8^+^ cytotoxic T cells by permitting their entry into an immunosuppressive microenvironment they would otherwise not encounter. Consequently, the immunosuppressive nature of the TME likely drives T cell exhaustion, curtailing effective tumor eradication upon tumor infiltration of immune cells. This dynamic explains the increased ICB sensitivity in YAP/TAZ-deficient tumors and the resistance seen in YAP/TAZ-proficient tumors, where T cell exclusion persisted.

In conclusion, our study provides proof of concept for an effective therapeutic strategy targeting YAP/TAZ in advanced cancers, particularly when combined with ICB. The synergy between YAP/TAZ depletion and ICB can be explained by a 2-hit model: YAP/TAZ inhibition/depletion enabled T cell infiltration, but these T cells were rapidly exhausted — a state reversed by ICB (Figure 9H). These findings suggest that YAP/TAZ inhibition has the potential to convert ICB-resistant tumors into ICB-responsive ones, thereby contributing to the development of more effective immune checkpoint therapies and new combinatorial treatments for targeting the oncogenic TEAD-YAP/TAZ complex.

## Methods

### Sex as a biological variable

Both male and female mice were included in these studies. Because no sex-dependent differences in response to the intervention were detected, data from both sexes were combined for analysis. The only exception was the experiments shown in Figure 6, C and D, which were conducted exclusively in male mice.

Additional details on methods are provided in the Supplemental Methods.

### Study design

This study was designed to evaluate the safety, side effects, and efficacy of YAP/TAZ inhibition in preclinical mouse models. Mice undergoing long-term YAP/TAZ depletion were weighed and scored weekly, unless clinical scoring was applied. Animals with a clinical score of 1 or 2 were monitored daily; a score of 3 defined the humane endpoint.

The body weight–based clinical scoring system was defined as follows: score of 0: no weight loss; score 1: 0%–10% weight loss; score of 2: 10%–20% weight loss; score of 3: greater than 20% weight loss (humane endpoint).

For CCA tumor models, animals were scored weekly from days 0–25, and then every 2–3 days. Monitoring intensified upon reaching a score of 1 or higher. The tumor burden–based scoring system was as follows: score of 0: normal body condition; score of 1: mild abdominal swelling over liver region; score of 3: marked abdominal distension with palpable liver tumors (humane endpoint).

All animals were also monitored according to the animal welfare guidelines of the Leibniz Institute on Aging – FLI, assessing general condition, grooming, posture, pain indicators, behavior, eyes, body openings, fur, and skin.

In all experiments, mice were randomized into treatment and control groups. The study was designed to evaluate the potential of YAP/TAZ inhibition in a preclinical mouse models in terms of safety, side effects, and efficacy.

### Mouse strains

The TRE-tGFP-shYAP1.351 (shY1), TRE-tGFP-shYAP1.322 (shY2), TRE-tGFP-shWwtr1.2056 (shT1), TRE-tGFP-shWwtr1.4587 (shT2), and TRE-tGFP-shRen mouse lines were generated and purchased from Mirimus. In these lines, tGFP served as a fluorescent marker, and a specific shRNA was expressed from a promoter containing a tetracycline response element (TRE). The knockin allele iss located downstream of the *Col1a1* locus (17). The ubiquitous doxycycline-inducible expression of the shRNAs is facilitated by the reverse tetracycline-controlled transactivator 3 (rtTA3), which is driven by a CAG promoter. The animals used in the experiments carried knockin alleles that expressed shRNAs targeting *Yap1* and *Taz*, while the control animals carried knockin alleles expressing an shRNA targeting *Renilla* luciferase. To ensure tissue-specific expression of the shRNAs, a Rosa26-lox-Stop-lox (LSL) cassette upstream of rtTA3 was used (23). LSL (RRID:IMSR_JAX:029633) and CAG-rtTA3 (RRID:IMSR_JAX:016532) mouse lines were purchased from Mirimus. CAF-specific expression was achieved by crossing the shRNA lines with Col1a2-Cre transgenic mice (42). Col1a2-Cre mice were obtained from Peter Angel (DKFZ, Heidelberg, Germany). For tumor cell–specific shRNA expression, Cre recombinase was delivered through HDTVI plasmid mix. iCas9 animals were purchased from The Jackson Laboratory (JAX no. 029476). These animals harbor an inducible TRE-Cas9 allele that is downstream of the *Col1a1* locus. When combined with the CAG-rtTA3 transgene, doxycycline administration enables ubiquitous expression of Cas9. Control animals were sex and age matched. Mice were housed, fed, and treated according to the guidelines of the Leibniz Institute on Aging – FLI.

### Plasmids

The pCMV(CAT)T7-SB100 plasmid that expresses hyperactivate sleeping beauty transposase was purchased from Addgene (Addgene, 34879). Myristolated, HA-tagged AKT, Myc-tagged NICD were amplified by PCR from pT3-myr-AKT-HA (Addgene, 31789) and pT3-EF1aH NICD (Addgene, 86500) and subcloned into the SfiI restriction site of the pSBbi-Pur vector (Addgene, 60523). Puromycin resistance was removed from the sleeping beauty vectors by EcoRI/BsrGI restriction digest followed by blunting and ligation.

To generate a bispecific Sleeping Beauty vector that expresses Myc-tagged NICD under the control of the EF1α promoter and a U6-driven sgRNA cassette, the following fragments were subcloned by Gibson assembly into the SfiI/EcoRI restriction sites of the pSBbi-Pur vector (Addgene, 60523): the EF1α promoter was amplified by PCR from the pSBbi-Pur vector (Addgene, 60523), Myc-tagged NICD was amplified from pT3-EF1aH NICD (Addgene, 86500), and the U6-SapI-sgRNA cassette was amplified from PX552 (Addgene, 60958). To restore *TGFB2* expression following depletion of YAP/TAZ in tumor cells, murine *Tgfb2* was cloned from cDNA, together with an IRES element, into the pSBbi-myr-AKT-HA vector to generate tumors overexpressing *TGFB2*. To assess the enhancer activity of the putative *Tgfb2* enhancer (gDNA: mm10: chr1:186,568,124–186,568,430), the fragment was amplified from genomic DNA and cloned upstream of the minimal promoter in the pGL4-23 vector (Promega, 9PIE841) using HindIII and XhoI restriction sites. Plasmids used within this study are listed in Supplemental Table 13.

### sgRNA design

The ChopChop tool was used for sgRNA design (25). sgRNAs were subcloned into the pSBbi-Myc-NICD-sgRNA vector (cut with SapI) and Gibson assembly of a 70 mer oligonucleotide according to the following design: ATCTTGTGGAAAGGACGAAACACCG, 20 nt sgRNA, GTTTTAGAGCTAGAAATAGCAAGTT. Oligonucleotides used for sgRNA cloning are listed in Supplemental Table 8.

### Hydrodynamic tail vein injection

For the delivery of Sleeping Beauty–based plasmids to the murine liver, hydrodynamic tail vein injection was performed. Mice were anesthetized using isoflurane. Specifically, 25 μg pSBbi-Myr-AKT-HA, 25 μg pSBbi-Myc-NICD, and 10 μg pCMV-SB100 plasmids were diluted in sterile Ringer’s lactate solution. The total volume was adjusted to 10% of the animal’s body weight and injected within 6–8 seconds. Mice were kept on a heating plate at 37°C until they recovered from anesthesia. Mice were monitored for 3 hours after HDTVI and then daily until they reached appropriate time points.

### Doxycycline administration

To induce expression of shRNAs in shYT animals or Cas9 in iCas9 animals, mice were fed doxycycline-containing chow (625 mg/kg doxycycline hyclate; Sniff, 326652) for the indicated duration.

### Anti-CD8 and anti–PD-1 administration

To deplete CD8^+^ T cells, animals were injected i.p. with 500 μg neutralizing monoclonal anti-CD8 antibody (clone YTS169) at the indicated time points. Successful CD8^+^ T cell depletion was confirmed by flow cytometric analysis of peripheral blood. An anti–mouse PD-1 antibody (clone RMP1-14, Bio X Cell, BE0146) was administered i.p. at a dose of 10 mg/kg body weight at the indicated time points.

### VT104 administration

VT104 (Vivace Therapeutics) was freshly formulated daily as a suspension in a vehicle composed of 5% DMSO, 10% Solutol (MilliporeSigma), and 85% glucose (5%) in water (D5W). To prepare the formulation, 3.35 mg VT104 powder was weighed into a vial and mixed with 50 μL DMSO by vortexing for 5 minutes. Subsequently, 100 μL Solutol was added and vortexed for an additional 2 minutes. The suspension was sonicated for 10 minutes, followed by the addition of 850 μL D5W, and vortexed for 2 minutes. A final 10-minute sonication ensured a homogeneous suspension of approximately 1 mL (~3.3 mg/mL). To achieve the target concentration of 2 mg/mL, 667 μL vehicle was added, resulting in a total volume of approximately 1.67 mL, which was sufficient to dose 5 mice at 10 mg/kg with a dosing volume of 5 μL/g body weight. The formulation was prepared immediately prior to administration and used within 1–2 hours at room temperature. For lower dose administrations (1 mg/kg), the 10 mg/kg stock solution was diluted 1:10 with vehicle and administered accordingly. VT104 was administered by oral gavage within 2 hours of formulation.

### Whole-body irradiation and EdU administration

To assess the effect of YAP/TAZ depletion on small intestine regeneration, animals were exposed to a sublethal dose of γ irradiation (10 Gy) using a GC40 irradiation chamber (cesium-137 source). After 4 days of regeneration, animals were sacrificed, and intestinal regeneration was evaluated. To quantify proliferating cells, animals were injected i.p. with EdU (10 mM stock solution; 100 μL per 10 g body weight) 2 hours prior to sacrifice.

### Histology, IHC, and image analysis

#### Fixation, paraffin embedment, and H&E staining.

Mouse livers were fixed in 10% formalin (3.7% formaldehyde in 1× PBS) for at least 48 hours at 4°C. Following fixation, livers were dehydrated and embedded in paraffin. Paraffin sections (4 μm thickness) were deparaffinized and processed for either H&E staining or IHC using specific primary antibodies.

#### IHC.

For IHC, antigen retrieval was performed using either citrate buffer (pH 6) or Tris-EDTA (TE) buffer (pH 9), depending on the antibody. Sections were boiled in a Coplin jar using a microwave for 12 minutes, followed by a 30-minute incubation at room temperature. After cooling down, sections were blocked in 3% BSA in PBS with 0.1% Tween 20 (PBST) for 2 hours at room temperature. Primary antibodies were diluted in blocking buffer to the appropriate concentrations and incubated on the sections overnight at 4°C. The following primary antibodies were used: YAP1 (Cell Signaling Technology, 14074; citrate pH 6; 1:200), Sox9 (MilliporeSigma, 5535; citrate pH 6; 1:200); CK19 (DSHB TROMA-III; TE pH 9; 1:15); CD3 (Abcam, ab16669; citrate pH 6; 1:150); and CD8 (Cell Signaling Technology, 98941S; citrate pH 6; 1:200). The following day, sections were washed 3 times for 5 minutes each in PBST and incubated with a secondary antibody conjugated to HRP (1:1,000 in PBST) for 1 hour at room temperature. After washing, visualization was performed using DAB chromogen (Vector Laboratories) according to the manufacturer’s protocol, followed by counterstaining with hematoxylin. Sections were then dehydrated and mounted using a xylene-based mounting medium.

#### EdU staining.

EdU staining was performed using the Click-iT EdU Cell Proliferation Kit (Thermo Fisher Scientific) according to the manufacturer’s instructions. Slides were imaged using an automated Axio Scan.Z1 microscope (Zeiss).

#### Picrosirius red staining for collagen visualization.

To visualize collagen fibers in CCA tumor sections, Picrosirius red staining was performed. Tissue sections were first deparaffinized as previously described. Nuclear counterstaining was carried out using Weigert’s hematoxylin for 8 minutes, followed by a 10-minute rinse under running tap water. Collagen fibers were stained with a saturated Picrosirius red solution (0.5 g Picrosirius red dissolved in 500 mL saturated aqueous picric acid) for 1 hour at room temperature. Sections were then rinsed twice with acidified water (prepared by adding 5 mL glacial acetic acid to 1 L tap water) to remove excess dye. Finally, sections were dehydrated through graded alcohols, cleared in xylene, and mounted with a xylene-based mounting medium.

#### Periodic acid–Schiff staining.

The periodic acid–Schiff (PAS) reaction was used to detect polysaccharides (e.g., glycogen). Tissue sections were deparaffinized, rehydrated through a graded ethanol series, and incubated in 0.5% periodic acid solution for 5–10 minutes to oxidize glycols to aldehydes. Sections were then rinsed in distilled water and treated with Schiff’s reagent for 15–20 minutes, followed by thorough washing in running tap water to develop the magenta color. Nuclei were counterstained with hematoxylin. Then, sections were evaluated, scanned, and photographed using the Panoramic II scanner (3DHISTECH, The Digital Pathology Company).

#### Histological scoring of liver, kidney and heart.

A morphological evaluation was performed using hematoxylin and eosin staining to identify tissue alterations across different organs. In the kidneys, the focus was on detecting signs of tubular cell degeneration. Liver sections were examined for the presence of inflammation, hepatocyte degeneration, and necrosis. In the heart, attention was given to identifying degenerative changes and necrosis in cardiomyocytes. The lesions (tubular cell degeneration, hepatic inflammation, hepatocyte degeneration, hepatic necrosis, degenerative changes, and necrosis in cardiomyocytes) were evaluated by examining at least 10 microscopic fields at ×20 magnification, using histological scoring systems. In particular, score 0 (absent lesions or no inflammatory foci); score 1 (mild lesions or <2 foci per ×20 field); score 2 (moderate lesions or 2–4 foci per ×20 field); and score 3 (severe lesions or >4 foci per ×20 field).

### 3C assays

Chromosome conformation capture (3C) assays were performed as previously described (43). In brief, 1 × 10^6^ cells derived from CCA organoids were used to generate 3C libraries (43). Cells were fixed with 2% formaldehyde for 10 minutes at room temperature, and crosslinking was quenched by adding 1/8 volume of 1 M ice-cold glycine. Cells were washed once with ice-cold PBS and resuspended in 500 μL cold lysis buffer (10 mM Tris-HCl pH 8.0, 10 mM NaCl, 0.2% NP-40, 1× protease inhibitor cocktail), followed by incubation on ice for 20 minutes. Lysed cells were pelleted at 15,000*g* for 10 minutes at 4 °C, and the supernatant was discarded. The pellet was resuspended in 175 μL digestion mix (152.5 μL ddH_2_O, 20 μL 10× DpnII buffer, 2.5 μL 20% SDS) and incubated at 37°C with shaking (1,400 rpm) for 1 hour. Triton X-100 was then added to a final concentration of 1.7%, followed by an additional 1-hour incubation under the same conditions. Chromatin digestion was performed by adding 3 μL DpnII enzyme (50,000 U/mL) and incubating for 2 hours at 37°C with shaking. An additional 3 μL fresh enzyme was added after 2 hours, and digestion was continued overnight. The following day, another 3 μL DpnII was added, and incubation continued for 3 hours. The enzyme was subsequently heat inactivated at 65°C for 20 minutes. For ligation, 161 μL ligation mix (125 μL ddH_2_O, 36 μL 10× ligation buffer) was added to each sample, followed by 4 μL T4 DNA ligase. Samples were incubated overnight at 16°C with gentle shaking (300 rpm). Crosslinks were reversed by adding 20 μL Proteinase K and incubating overnight at 65°C with shaking (1,400 rpm). The following day, 4 μL RNase A (10 mg/mL) was added, and samples were incubated for 30 minutes at 37°C. DNA was purified by phenol-chloroform extraction with ethanol precipitation. The resulting DNA pellet was resuspended in 100 μL 0.1× TE buffer. Digestion efficiency was assessed using 1 μL of the sample on a Fragment Analyzer (Agilent Technologies). 3C primers were validated using gBlocks as positive controls, and specific amplification products are shown in Figure 8F (right panel). For interaction analysis, 1 μL of the 3C library was used as input for PCR amplification using Phusion Hot Start II DNA Polymerase with HF buffer to detect enhancer-promoter interactions.

### Bulk RNA-seq analysis

For bulk RNA-seq, TRIzol-purified total RNA was additionally purified using the RNeasy Mini Kit (Qiagen) including an on-column DNase I digestion for 15 minutes at room temperature to remove residual genomic DNA. RNA quality and concentration were assessed using the Fragment Analyzer (Agilent Technologies). For library preparation, 1 μg high-quality RNA was used as input for mRNA enrichment with the NEBNext Poly(A) mRNA Magnetic Isolation Module (New England Biolabs). mRNA was then processed using the NEBNext Ultra II RNA Library Prep Kit in combination with NEBNext Multiplex Oligos for Illumina (E6440, New England Biolabs) following the manufacturer’s protocol. Final libraries were quantified using the Agilent 2100 Bioanalyzer (Agilent Technologies) and sequenced as 75 bp single-end reads on an Illumina NextSeq 500 platform. Sequencing reads were extracted in FASTQ format using bcl2fastq (version 1.8.4, Illumina). Adapter trimming, size selection (retaining reads >25 nucleotides), and quality filtering (Phred score >30) of FASTQ files were performed using Cutadapt. Reads were subsequently aligned to the mouse genome (mm10) with Bowtie2 (version 2.2.9) using default settings. Read counts were extracted in R using the countOverlaps function from the GenomicRanges package. Differential gene expression analysis was carried out with DESeq2 (version 3.26.8) using default parameters.

### scRNA-seq analysis

#### Effect of acute YAP/TAZ-depletion on the TME (10x Genomics).

For single-cell RNA-seq, tumor-bearing livers were digested and processed as described in the CCA isolation protocol. Following dead cell removal, viable cells were incubated with Fc receptor blocking reagent (Miltenyi Biotec) for 10 minutes on ice to prevent nonspecific antibody binding. Subsequently, cells were labeled with 1 μg anti–mouse hashtag antibodies (TotalSeq-B, BioLegend) for 30 minutes on ice. After labeling, cells were washed twice with PBS and resuspended in 1× PBS. The final cell concentration was adjusted to 1,000 cells/μL for downstream single-cell library preparation. Single-cell encapsulation and barcoding were performed using the 10x Genomics Chromium Controller in combination with the Chromium Next GEM Single Cell 3′ Reagent Kits version 3.1 (10x Genomics) according to the manufacturer’s protocol. Libraries were sequenced on an Illumina platform with a targeted depth of approximately 50,000 reads per cell. Raw sequencing data were processed using Cell Ranger (version 6.1.2, 10x Genomics) with the mm10 mouse reference genome for demultiplexing, alignment, and generation of gene–cell count matrices. Downstream analysis was performed using the Seurat R package (version 4.3). Cells with fewer than 500 detected genes, more than 5,000 detected genes, or with greater than 10% mitochondrial gene expression were excluded from further analysis. Hashtag oligonucleotide–based (HTO-based) sample demultiplexing was performed using Seurat’s HTODemux function. After quality control, datasets were normalized using SCTransform, and samples were integrated using Seurat’s integration workflow based on canonical correlation analysis (CCA). Dimensionality reduction was performed using principal component analysis (PCA), followed by uniform manifold approximation and projection (UMAP) for visualization. Clustering was conducted using a shared nearest neighbor (SNN) graph and the Louvain algorithm.

#### Effect of acute YAP/TAZ depletion and anti–PD-1 treatment on the TME (Singleron).

Cell isolation and processing were performed as previously described in the 10x Genomics section, with the exception that hashtag antibody labeling was omitted. Following cell isolation, the final cell concentration was adjusted to 750 cells/μL for downstream single-cell library preparation. Single-cell encapsulation, barcoding, and cDNA synthesis were performed using the Singleron GEXSCOPE Single Cell RNA Library Kit V2, according to the manufacturer’s instructions. Prepared libraries were sequenced on an Illumina platform, targeting a read depth of approximately 25,000 reads per cell. Raw sequencing data were processed using Cell Scope (Singleron) with the mm10 (mouse) reference genome, which included steps for sample demultiplexing, read alignment, and generation of gene cell count matrices. Downstream analysis (e.g., quality control, clustering, and differential expression analysis) was conducted as described in the 10x Genomics section above.

#### Using the shYT T cell signature to stratify TCGA CCA patients.

The analysis was performed as described previously (44). Briefly, genes with differential expression between shYT and shRen T cells were identified using the FindMarkers function in Seurat. The Cancer Genome Atlas (TCGA) RNA-seq data were centered to the median per gene. The distribution of all genes in the gene set was determined per patient to generate a ranked gene set. The positive or negative enrichment score (ES) of this gene set was subsequently determined using the Kolmogorov-Smirnov test for each patient. A permutation-based algorithm was then used to generate 10^5^ random gene sets of equal size. This was used to determine an empirical *P* value: a *P* value of 10^–5^ was assigned if the ES of the tested gene set was greater than that of every permuted gene set. The association score per patient was then calculated using the following formula: –log_10_ (*P* value) × the direction of association, where –1 indicates a negative and +1 indicates a positive association, respectively. On the basis of the association score, TCGA patients were stratified into high-expression or low-expression groups, and survival was calculated using the R “survival” package.

### CUT&RUN

CCA cells were isolated as previously described (see Cholangiocarcinoma Cell Isolation, Supplemental Methods). Following EpCAM enrichment, tumor cells were counted and resuspended at a concentration of 2 × 10^6^ cells in wash buffer (20 mM HEPES, pH 7.5; 150 mM NaCl; 0.5 mM spermidine; protease inhibitor cocktail). For each CUT&RUN reaction, 2 × 10^5^ cells were used. Cells were incubated with 10 μL activated concanavalin A–coated magnetic beads (Polysciences) for 10 minutes at room temperature on a hula mixer with constant agitation. Following bead binding, samples were placed on a magnetic stand to remove the supernatant. Bead-bound cells were resuspended in 100 μL antibody buffer (20 mM HEPES, pH 7.5; 150 mM NaCl; 0.5 mM spermidine; 0.01% [w/v] digitonin; 2 mM EDTA; protease inhibitor), and primary antibodies were added as follows: anti-TEAD1 (Cell Signaling Technology, 12292); anti-YAP1 (Cell Signaling Technology, 14074); anti-H3K27Ac (Abcam, ab4729); and anti-H3K4me1 (Cell Signaling Technology, 5326). A species-matched IgG was used as a negative control. Samples were incubated overnight at 4°C on a HulaMixer (Thermo Fisher Scientific) set at a 50° tilt and 1 rpm. The next day, samples were washed twice with digitonin wash buffer (20 mM HEPES, pH 7.5; 150 mM NaCl; 0.5 mM spermidine; 0.01% digitonin; protease inhibitor). Protein A/G-MNase (pAG-MNase; 1 μg/mL) was added in digitonin wash buffer, and samples were incubated for 1 hour at 4°C with gentle agitation. After incubation, cells were washed 3 times with digitonin wash buffer and twice with low-salt buffer (20 mM HEPES, pH 7.5; 0.5 mM spermidine; 0.01% digitonin). Nuclease activation was performed by transferring the samples to calcium-containing buffer (3.5 mM HEPES, pH 7.5; 10 mM CaCl_2_; 0.01% digitonin; protease inhibitor) and incubating on ice for 1 hour with continuous mixing. Cleaved DNA fragments were released by incubation at 37°C for 30 minutes in STOP buffer (170 mM NaCl; 20 mM EGTA; 0.01% digitonin; 50 μg/mL RNase A; 25 μg/mL Glycogen Blue). Supernatants were collected, and DNA was purified by phenol-chloroform extraction. Library preparation was performed using the NEBNext Ultra II DNA Library Prep Kit for Illumina (New England Biolabs, E7645) following the manufacturer’s protocol. Library quality was assessed using the Fragment Analyzer (Agilent Technologies), and equimolar pooling of libraries was performed. Pooled libraries were gel purified, quantified on an Agilent 2100 Bioanalyzer, and sequenced as 75 bp paired-end reads on an Illumina NextSeq 500 platform.

CUT&RUN analysis was conducted as previously described (45). Briefly, adapter and quality trimming were performed using Cutadapt. Reads were aligned to the mm10 genome and a repeat-masked *E*. *coli* genome (used as spike-in) using Bowtie2. Reads with inserts of less than 120  bp mapped to mm10 were extracted (deepTools), and a scaling factor based on the *E*. *coli* spike-in was used to generate normalized bigWig files. Peak calling was performed using Genrich using the following parameters: q 1 × 10^–4^ – a 700. For CDF analysis, TEAD1 signals at categorized peaks (based on RNA-seq regulation, downregulation, upregulation, or no regulation) were associated with the nearest TSS using BEDtools and plotted with ggecdf (ggpubr).

### OMNI ATAC-Seq

CCA cells were isolated as previously described (see Cholangiocarcinoma Cell Isolation, Supplemental Methods). A total of 5 × 10^4^ cells were pelleted in a prewetted 1.5 mL low-binding tube by centrifugation at 500*g* for 5 minutes at 4°C using a fixed-angle rotor. Cells were resuspended in 50 μL ice-cold ATAC-Resuspension Buffer (RSB: 10 mM Tris-HCl, pH 7.4; 10 mM NaCl; 3 mM MgCl_2_) supplemented with 0.1% (v/v) NP-40, 0.1% (v/v) Tween 20 and 0.01% (w/v) digitonin. The suspension was pipetted up and down 3 times and incubated on ice for 3 minutes to lyse the cells. To remove the lysis reagents, 1 mL ice-cold RSB containing 0.1% Tween 20 (without NP-40 or digitonin) was added, and the tube was gently inverted 3 times. Nuclei were pelleted by centrifugation at 500*g* for 10 minutes at 4°C, and the supernatant was carefully removed. Nuclei were resuspended in 50 μL transposition reaction mix containing 25 μL 2× TD buffer (Illumina), 2.5 μL Tn5 transposase (to reach 100 nM final concentration), 16.5 μL 1× PBS, 0.5 μL 1% digitonin, 0.5 μL 10% Tween 20, and 5 μL nuclease-free water. The mix was pipetted up and down 6 times to ensure homogeneous suspension. Samples were incubated at 37°C for 30 minutes at 1,000 rpm using a thermomixer. Following transposition, DNA was purified using the Zymo DNA Clean & Concentrator-5 Kit (Zymo Research) according to the manufacturer’s instructions. Libraries were amplified using the NEBNext 2× Master Mix (New England Biolabs) and assessed for quality using the Fragment Analyzer (Agilent Technologies). Oligonucleotides used for amplification of ATAC-seq libraries are listed in Supplemental Table 9. Final libraries were pooled in equimolar ratios and sequenced as single-end reads on the Illumina NextSeq 500 platform. For the ATAC-Seq analysis the nextflow pipeline (https://nf-co.re/atacseq/2.1.2/) was used. The differential peak analysis was performed using DiffChIPL (https://github.com/yancychy/DiffChIPL), and TF motif accessibility was analyzed with TOBIAS (46).

### Gene set enrichment analysis

Gene set enrichment was performed using the MSigDB GSEA tool (version 4.3.1) with a gene set enrichment analysis (GSEA) Preranked analysis.

### TCGA analysis

RNA-Seq data and the associated phenotype data were downloaded from https://xena.ucsc.edu/ For correlation of *TGFB2* expression with YAP/TAZ activity, the YAP/TAZ target signature of 22 genes was used that was derived from a meta-analysis of 33 cancer types (24). As this signature contained *TGFB2*, this gene was removed from the gene set for further correlation analysis. To perform the correlation analysis, we computed YAP/TAZ activity scores per tumor type using the GSVA package (version 3.21) from Bioconductor in R. The resulting YAP/TAZ activity scores were then correlated (Pearson correlation) with *TGFB2* mRNA expression per tumor type.

### Quantifications and statistics

Statistical analyses were conducted using R (version 4.1.0). Unless otherwise specified, graphs depict the mean along with the SEM. The following statistical tests were used in this study: 2-tailed Welch’s t test with Benjamini-Hochberg correction, paired 2-tailed Welch’s t test with Benjamini-Hochberg correction, 2-way ANOVA followed by Tukey’s HSD post hoc test, 1-way ANOVA followed by Tukey’s HSD post hoc test, Kruskal-Wallis test followed by Dunn’s post hoc test with Benjamini-Hochberg correction, log-rank test, and Wilcoxon rank-sum test. The specific statistical test applied is indicated in the corresponding figure legend. A *P* value of less than 0.05 was considered significant. Each experiment was performed with a minimum of 3 independent biological replicates, unless stated otherwise in the figure legend.

Image quantifications were performed in a blinded manner. Tumor area, collagen content, and the desmoplastic index were assessed using H&E-stained sections (for tumor area and desmoplastic index) or Picrosirius red–stained sections (for collagen fibers). Images were analyzed using ImageJ (NIH) in combination with the Trainable Weka Segmentation plugin. The area of all tumor nodules and collagen fibers was measured in pixels and normalized to the total liver area. To calculate the desmoplastic index, the percentage of tumor stroma was measured in pixels and normalized by the total tumor area.

### Study approval

All mouse studies were performed at the Leibniz Institute on Aging – FLI, approved by the Institutional Animal Welfare Committee and the federal state of Thuringia, and conducted in accordance with national regulations and institutional guidelines. Animal experiments presented in this manuscript were performed under the following approved licenses: FLI-17-017, FLI-21-015, FLI-22-012, FLI-23-017, and FLI-24-002.

### Data and materials availability

All data underlying this manuscript are provided in the Supporting Data Values file. The Next-generation sequencing data generated in this study have been deposited in the Gene Expression Omnibus (GEO) database (GSE301501). The histology images have been deposited in the BioImage Archive (accession code: S-BIAD2127).

## Author contributions

MJ and BVE conceptualized the study. MJ, BVE, SM, MR, and TT developed the methodology. MJ, VH, DI, OP, AR, KMK, MTW, ACV, KB, VD, TDG, CD, CJ, TL, AK, YK, TH, CR, EDN, and SKM performed the investigation. MJ and BVE carried out the visualization and data presentation. Funding was acquired by BVE. BVE administered and supervised the project. MJ and BVE wrote the original draft of the manuscript and reviewed and edited the final version.

## Conflict of interest

TTT reports employment with Vivace Therapeutics and has equity interest in Vivace Therapeutics.

## Funding support

- Wilhelm Sander-Stiftung grant 2022.084.1 (to BVE).
- Federal Ministry of Education and Research (BMBF), Germany grant 16GW0271K (to BVE).
- German Research Foundation grant EY 120/4-1 (to BVE).
- German Research Foundation grant EY 120/9-1 (to BVE).
- German Research Foundation grant EY 120/10-1 (to BVE).
- German Cancer Aid 70116078 (to BVE).
- Leibniz Collaborative Excellence K398/2021 (to BVE).
- German Research Foundation grant 318346496 (SFB1292 subproject TP21N, to MR and SM).
- BMFTR, funding program Photonics Research Germany “LPI-BT1-FSU” 13N15466 (to VD).

## Supplementary Material

Supplemental data

Unedited blot and gel images

Supporting data values

## Version 1 07/02/2026

In-Press Preview

## Version 2 09/01/2026

Electronic publication
