# Supplementary material for: Extracellular matrix reprogramming by the YAP/TAZ/TGF-β2 axis drives immune exclusion in cholangiocarcinoma models
Source: J Clin Invest. 2026 Jul 2;136(17):e201579. doi: 10.1172/JCI201579 (PMC13528937; doi:10.1172/JCI201579)
Supplement: Supplementary file 1 [file jci-136-201579-s272.pdf]

Extracellular matrix reprogramming by the YAP/TAZ/TGF-ß2 axis drives immune exclusion in cholangiocarcinoma models

Authors: Marco Jessen, KyungMok Kim, Marie Tollot-Wegner, Anita Cindric Vranesic, Cagla Dönmez, Celina Junker, Tina Lehmann, Advitiya Khandelwal, Yuliya Kurlishchuk, Tom Hünniger, Christin Ritter, Evaristo Di Napoli, Shyam Krishnan Murali, Konrad Bücking, Viktoria Haug, Sabine Muth, Tracy T. Tang, Andreas Rosenwald, Markus Radsak, Donato Inverso, Tanja Deckert-Gaudig, Volker Deckert, Orlando Paciello, Björn von Eyss*

*Corresponding author. Email: bjoern.voneyss@leibniz-fli.de

The PDF file includes:

Supplemental Figures 1 to 14
Supplemental Methods
Supplemental Tables 7 to 13

Other Supplemental Materials for this manuscript include the following:

Supplemental Tables 1 to 6

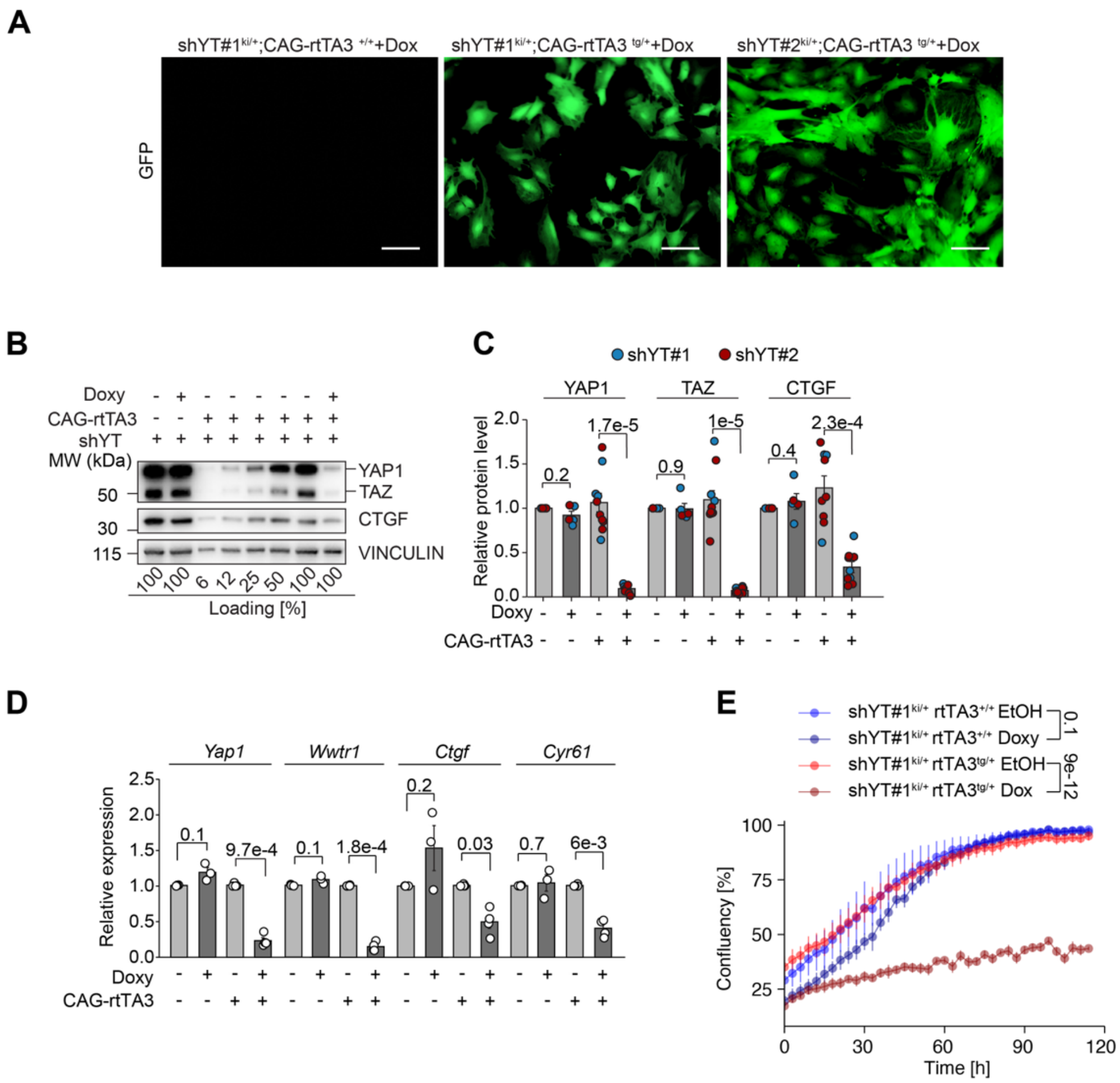

Supplemental Figure 1. Validation of YAP/TAZ knockdown in mouse embryonic fibroblasts (MEFs). (**A**) Representative images of MEFs shYT#1, shYT#2 mouse lines or control MEFs of the same lines lacking the reverse tetracycline transactivator (rtTA3), showing GFP expression. Scale bars, 50 µm.
(**B**, **C**) Representative immunoblots of MEF lysates harvested after 5 days of doxycycline-induced shRNA expression, showing protein levels of YAP1, TAZ, and CTGF. Control lysates were serially diluted to better quantify knockdown efficiency. Quantification across replicates (n = 3–6 per condition).
(**D**) RT–qPCR analysis of *Yap1*, *Wwtr1*, *Ctgf*, and *Cyr61* expression in shYT#1 MEFs (n = 3–4 per condition).
(**E**) Proliferation of shYT#1 MEF lines assessed by confluency measurements using an Incucyte imaging system (n = 3 per group).
Data represent mean ± SEM. Statistical analysis: Welch's t test with Benjamini-Hochberg correction (C, D); two-way ANOVA with Tukey HSD post hoc test (E).

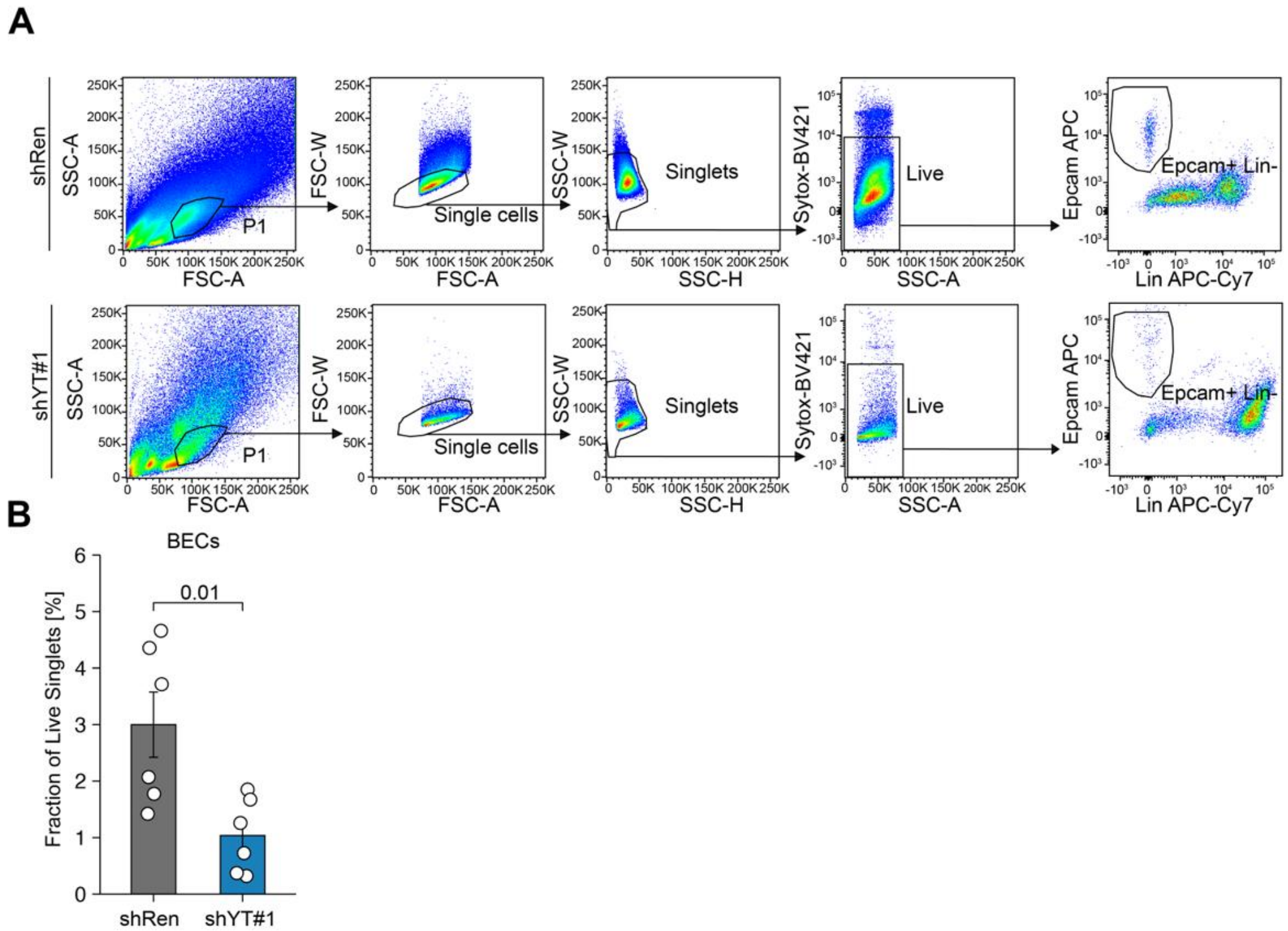

Supplemental Figure 2. YAP/TAZ depletion reduces biliary epithelial cells (BECs) in the liver, as confirmed by flow cytometry.

(**A**, **B**) Representative FACS profiles and gating strategy from livers of shRen (top) and shYT#1 (bottom) mice. Quantification of BECs from live singlet populations (n = 6 per group).

Data represent mean ± SEM. Statistical analysis: Welch's t test with Benjamini-Hochberg correction.

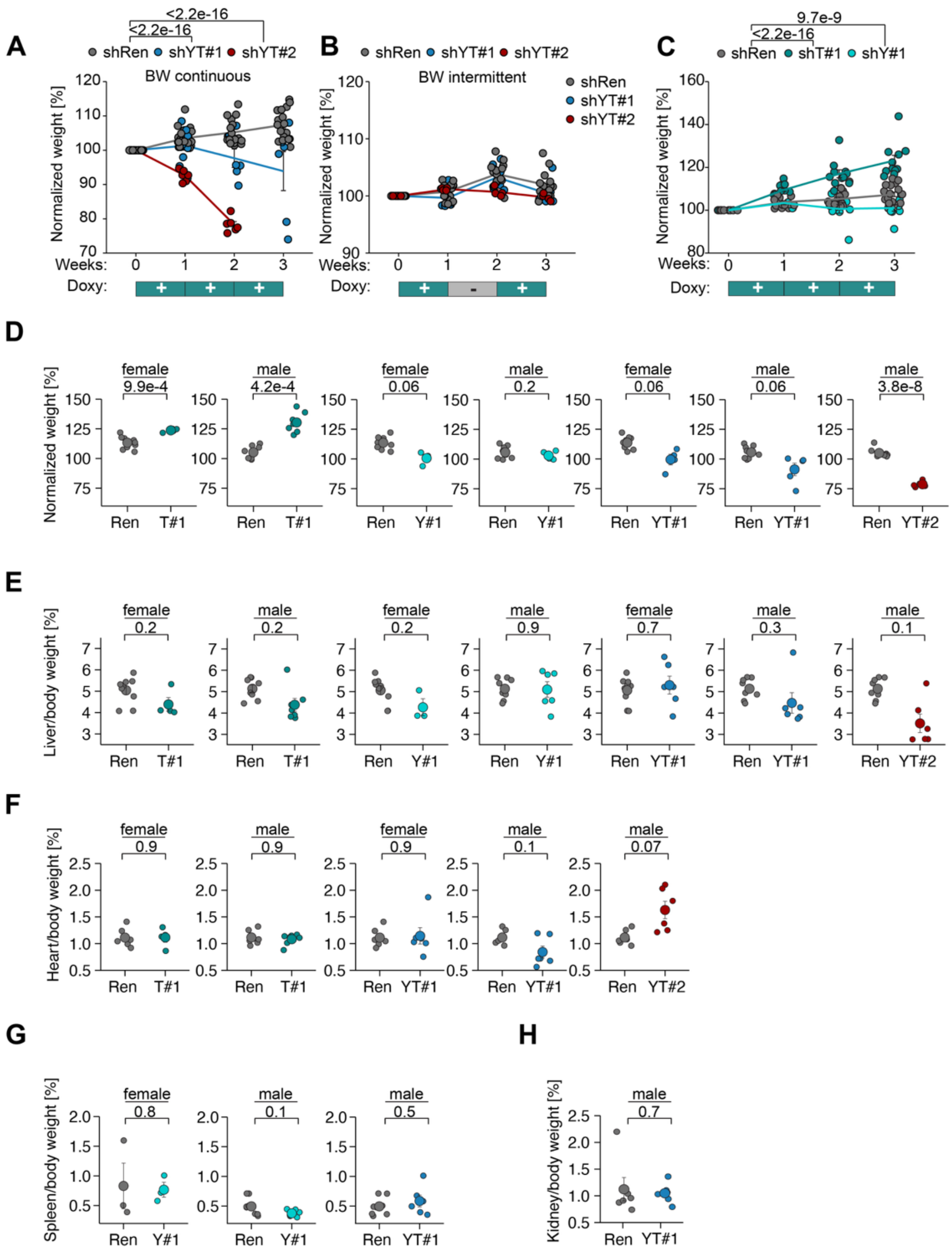

Supplemental Figure 3. Body weight loss following constitutive YAP/TAZ depletion.

(**A**) Change in body weight following continuous doxycycline-induced YAP/TAZ knockdown over 3 weeks (shRen, n = 18; shYT#1, n = 6; shYT#2, n = 6).

(**B**) Change in body weight following intermittent doxycycline administration (shRen, n = 15; shYT#1, n = 7; shYT#2, n = 4).

(**C**) Change in body weight over 3 weeks in mice with selective knockdown of YAP (shY#1), TAZ (shT#1) (shRen, n = 11; shY#1, n = 9; shT#1, n = 11).

(**D**) Body weight normalized to baseline after 4 weeks of doxycycline treatment for shYT#1, shY#1, and shT#1, or 2 weeks for shYT#2, stratified by sex. Each dot represents one animal.

(**E**) Liver weight normalized to body weight after 4 weeks (shYT#1, shY#1, shT#1) or 2 weeks (shYT#2) of knockdown, stratified by sex.

(**F**) Heart weight normalized to body weight after 4 weeks (shYT#1, shT#1) or 2 weeks (shYT#2), stratified by sex.

(**G**) Spleen weight normalized to body weight after 4 weeks (shYT#1, shY#1) or 2 weeks (shYT#2), stratified by sex.
(**H**) Kidney weight normalized to body weight after 4 weeks of YAP/TAZ depletion (shYT#1), stratified by sex.
Data represent mean ± SEM. Statistical analysis: two-way ANOVA (A–C); Welch's t test with Benjamini-Hochberg correction (D–I).

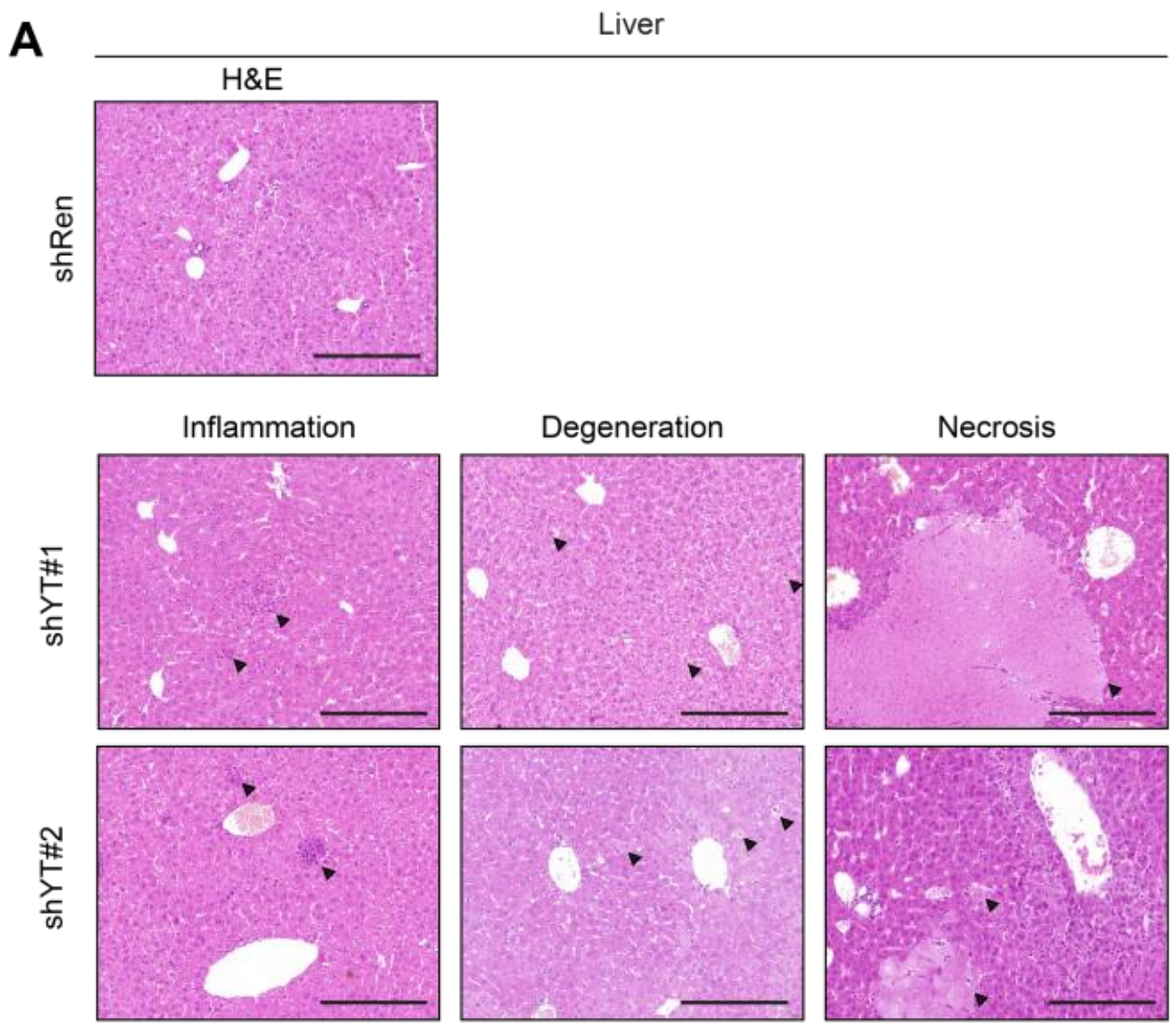

	Inflammation	Degeneration	Necrosis
shRen			
shYT#1	4 out of 5	5 out of 5	2 out of 5
shYT#2	2 out of 6	6 out of 6	6 out of 6

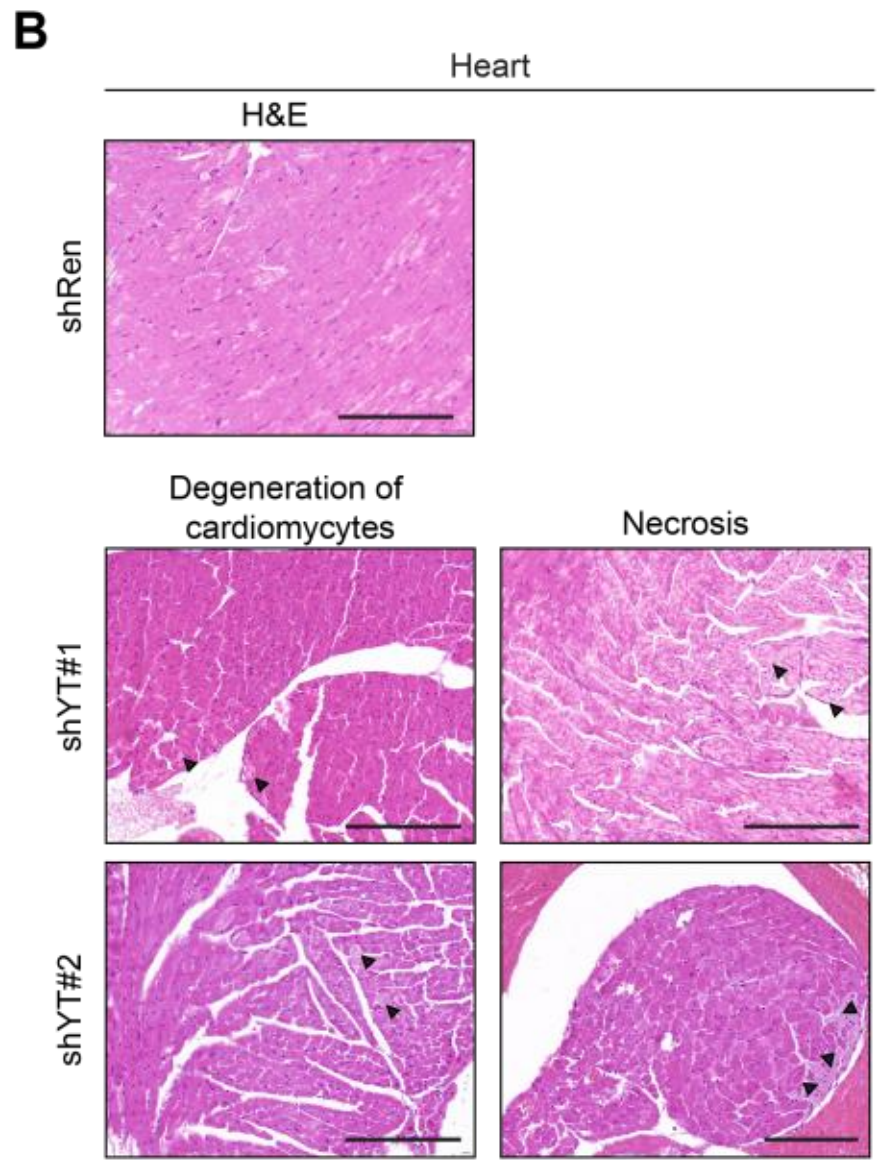

	Degeneration	Necrosis
shRen		
shYT#1	5 out of 5	5 out of 5
shYT#2	5 out of 6	5 out of 6

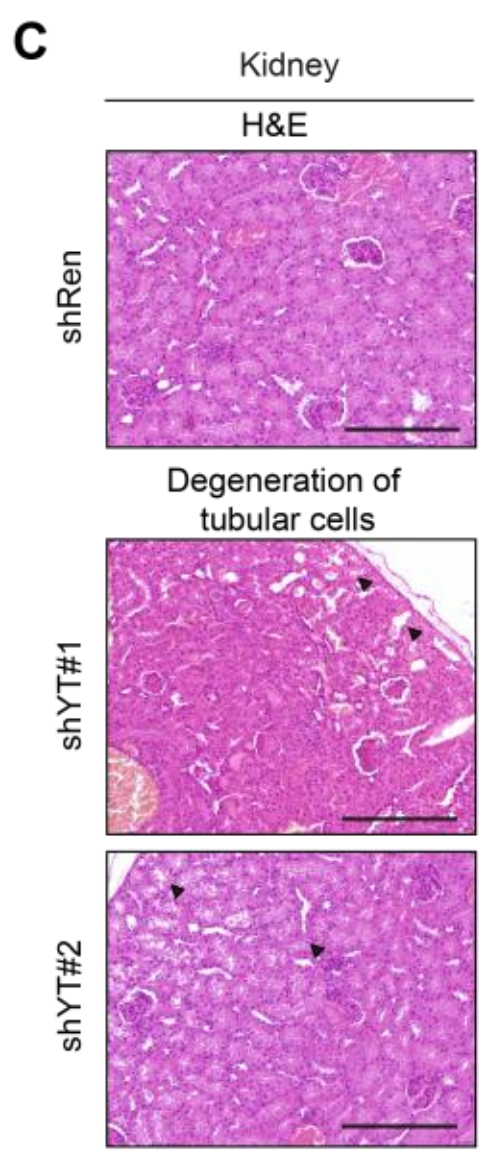

	Degeneration
shRen	
shYT#1	5 out of 5
shYT#2	6 out of 6

Supplemental Figure 4. Histopathological manifestations in liver, heart, and kidney following long-term YAP/TAZ depletion.
(**A**–**C**) Representative hematoxylin and eosin (H&E) staining of liver (A), heart (B), and kidney (C) sections from control (shRen) and YAP/TAZ-depleted (shYT#1, shYT#2) mice analyzed at their respective humane endpoints.
Liver sections show immune cell infiltration, parenchymal degeneration, and necrosis (arrows).
Heart tissue displays cardiomyocyte degeneration and necrotic areas (arrows).
Kidney sections reveal degeneration of tubular epithelial cells (arrows).
These findings reflect multi-organ pathology associated with sustained YAP/TAZ loss.
Scale bars, 200 μm.

Histological analysis revealed that shYT-deprived animals showed multifocal lesions in multiple organs.
<u>Cases shYT1#:</u>

- Liver showed severe multifocal to coalescent necrosis in 2 out of 5 cases; mild to moderate inflammation in 4 out of 5, composed of a multifocal lymphoplasmacytic infiltrate; mild to moderate hepatocyte degeneration with optically empty cytoplasm containing small, rounded vacuoles or granular material, which sometimes delocalizes the nucleus in the periphery in all cases.
- Heart showed mild to moderate compensatory hypertrophy of cardiomyocytes (degeneration) with variability in myofiber diameter, reduction in myofiber size (atrophy) with angular profile, and severe multifocal to coalescent necrosis in all cases.
- Kidney showed in all cases mild to moderate degeneration of tubular epithelial cells.

<u>Cases shYT2#:</u>

- Liver showed mild to moderate, multifocal to coalescent, necrosis in all cases; mild to moderate inflammation in 2 out of 6, composed of a multifocal lymphoplasmacytic infiltrate; mild to moderate hepatocyte degeneration with optically empty cytoplasm containing small, rounded vacuoles or granular material, which sometimes delocalizes the nucleus in the periphery in all cases.
- Heart showed mild to moderate compensatory hypertrophy of cardiomyocytes (degeneration) with variability in myofiber diameter, reduction in myofiber size (atrophy) with angular profile, and mild to moderate, multifocal to coalescent, necrosis in 5 out of 6 cases.
- Kidney showed in all cases mild to moderate degeneration of tubular epithelial cells.

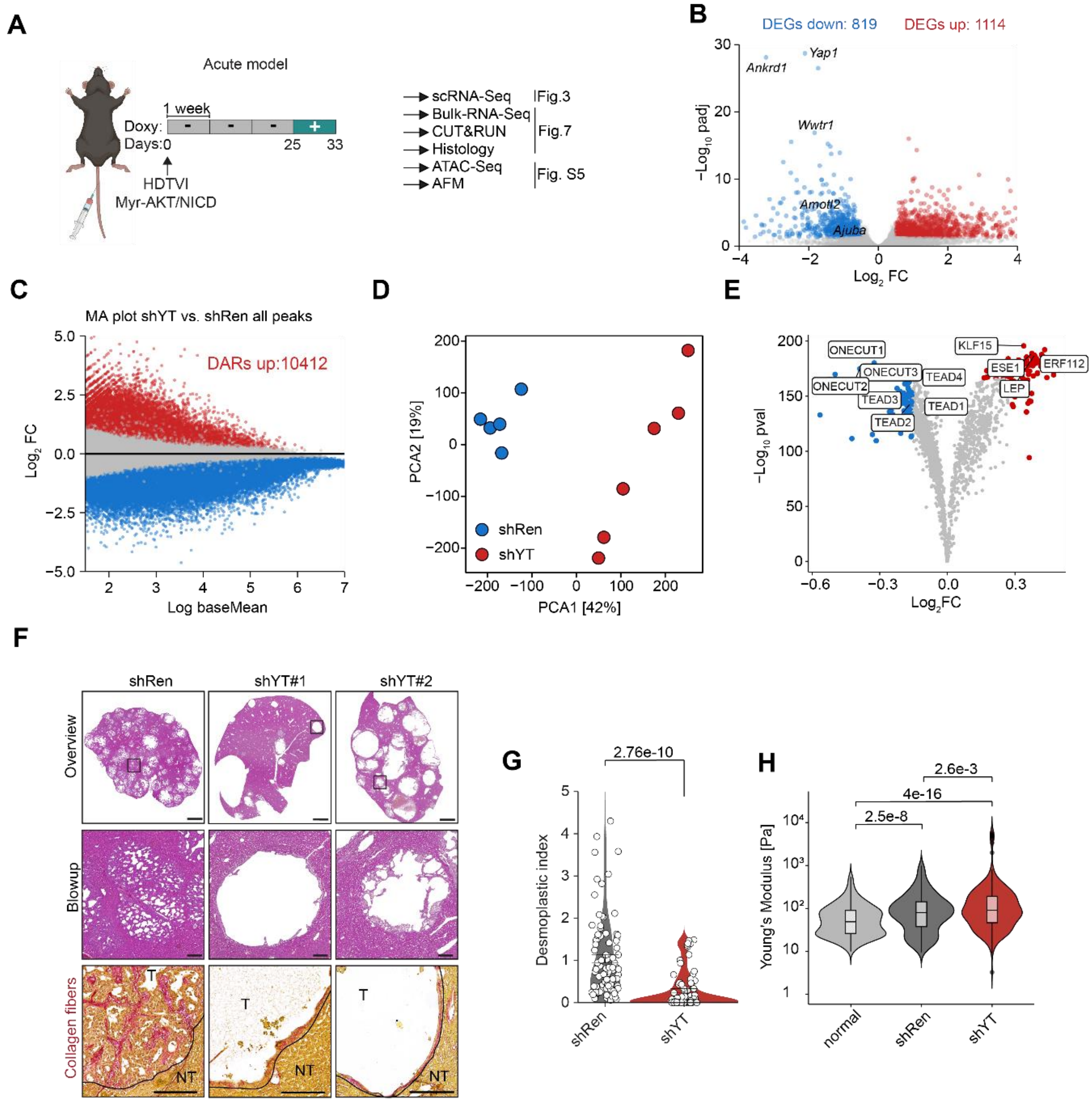

Supplemental Figure 5. Acute YAP/TAZ depletion in CCA tumor cells leads to profound changes in transcription, tissue architecture, stiffness and chromatin accessibility.

(**A**) Acute depletion model: YAP/TAZ depletion was initiated at day 25 after HDTVI and analysed at day 33 in the N-AKT CCA model. Atomic force microscopy (AFM).

(**B**) Acutely YAP/TAZ depleted tumor cells were isolated from tumor-bearing livers by EpCAM-based enrichment and subjected to RNA-Seq. Volcano plot shows downregulation of *Yap1*, *Wwtr1* (Taz), and canonical YAP/TAZ target genes (*Ankrd1*, *Amotl2*, *Ajuba*). (shRen, n = 4; shYT#1, n = 2 shYT#2, n = 2).

(**C**) MA plot showing differential chromatin accessibility in tumor cells after acute YAP/TAZ depletion, with 10,412 opening regions and 51,478 closing regions genome wide. Differentially accessible regions, DARs.

(**D**) Principal component analysis (PCA) plot demonstrating separation between control (shRen) and YAP/TAZ-depleted (shYT#2) tumor cells based on ATAC-Seq signals.

(**E**) TOBIAS footprinting analysis of differentially accessible regions. Volcano plot displays significantly enriched transcription factor motifs in closing (blue) and opening (red) regions. (shRen, n = 5; shYT#2, n = 6).

(**F**) Representative liver sections stained with H&E from tumor-bearing mice with acute YAP/TAZ depletion, showing low-magnification overviews (upper panel) and higher-magnification blowups (middle panel). Scale bars, 2 mm (overview), 200 μm (zoom-in).
Picro Sirius Red (lower panel) was used to stain collagen (red) in tumors. Scale bars, 100 μm. NT, non-tumor tissue; T, tumor tissue.
(**G**) Quantification of desmoplastic score in livers from control (shRen, n = 6) and YAP/TAZ-depleted animals (shYT#1, n = 2; shYT#2, n = 5). Statistical analysis: Welch's t test with Benjamini-Hochberg correction.
(**H**) Violin plots showing the distribution of Young's modulus values measured in normal liver tissue (normal, n = 5) and tumor tissue from control (shRen, n = 5) and YAP/TAZ-depleted animals (shYT#1, n = 2; shYT#2, n = 3). For atomic force microscopy (AFM) measurements, four independent areas (20 μm × 20 μm; 5 × 5 pixels) were analyzed per biological sample. Violin plots illustrate the distribution and density of the data. Boxplots show the median (center line), interquartile range (box), and whiskers extending to 1.5× the interquartile range. Data points beyond this range are shown as outliers. The y-axis is displayed on a log10 scale. Statistical analysis was performed using the Kruskal–Wallis test followed by Dunn's post hoc test.

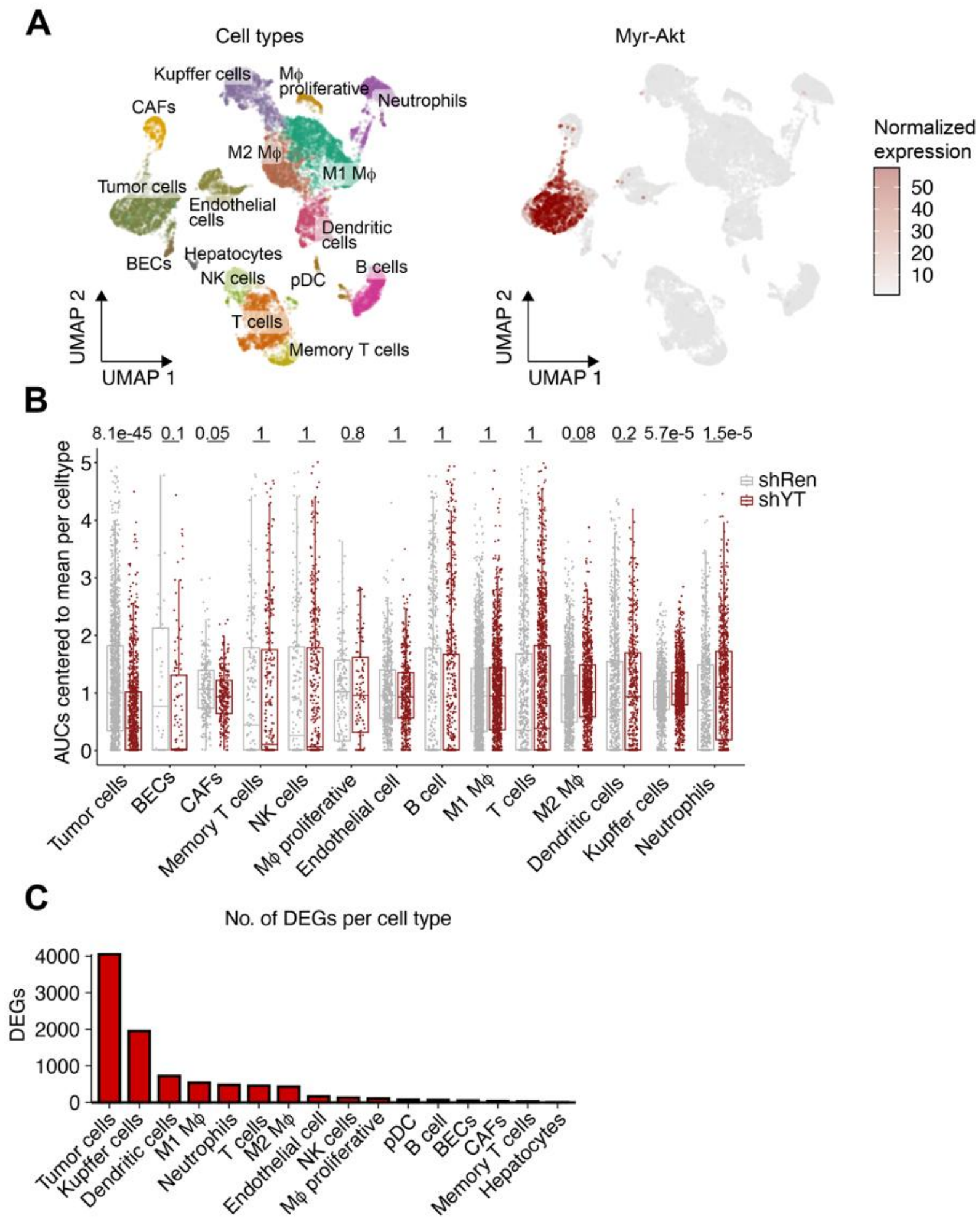

Supplemental Figure 6. Tumor cells exhibit the strongest transcriptomic changes after YAP/TAZ depletion.

(**A**) Acute YAP/TAZ depletion was induced in advanced CCA by doxycycline treatment from day 25 to 33. Single-cell RNA-seq was performed to assess transcriptomic changes within the TME. UMAP plots show an overview of identified cell types (left) and expression of Myr-AKT (right) to define the tumor cell cluster (shRen, n = 4; shYT#1, n = 2; shYT#2, n = 2).

(**B**) Box plots showing AUC scores for a conserved YAP transcriptional signature in individual cell types from shRen and shYT samples. AUC values are centered to the mean of every cell type. Boxplots show the median (center line), interquartile range (box), and whiskers extending to 1.5× the interquartile range. Data points beyond this range are shown as outliers. Statistical analysis: Wilcoxon rank test

(**C**) Bar plot displaying the number of differentially expressed genes across cell types following YAP/TAZ depletion.

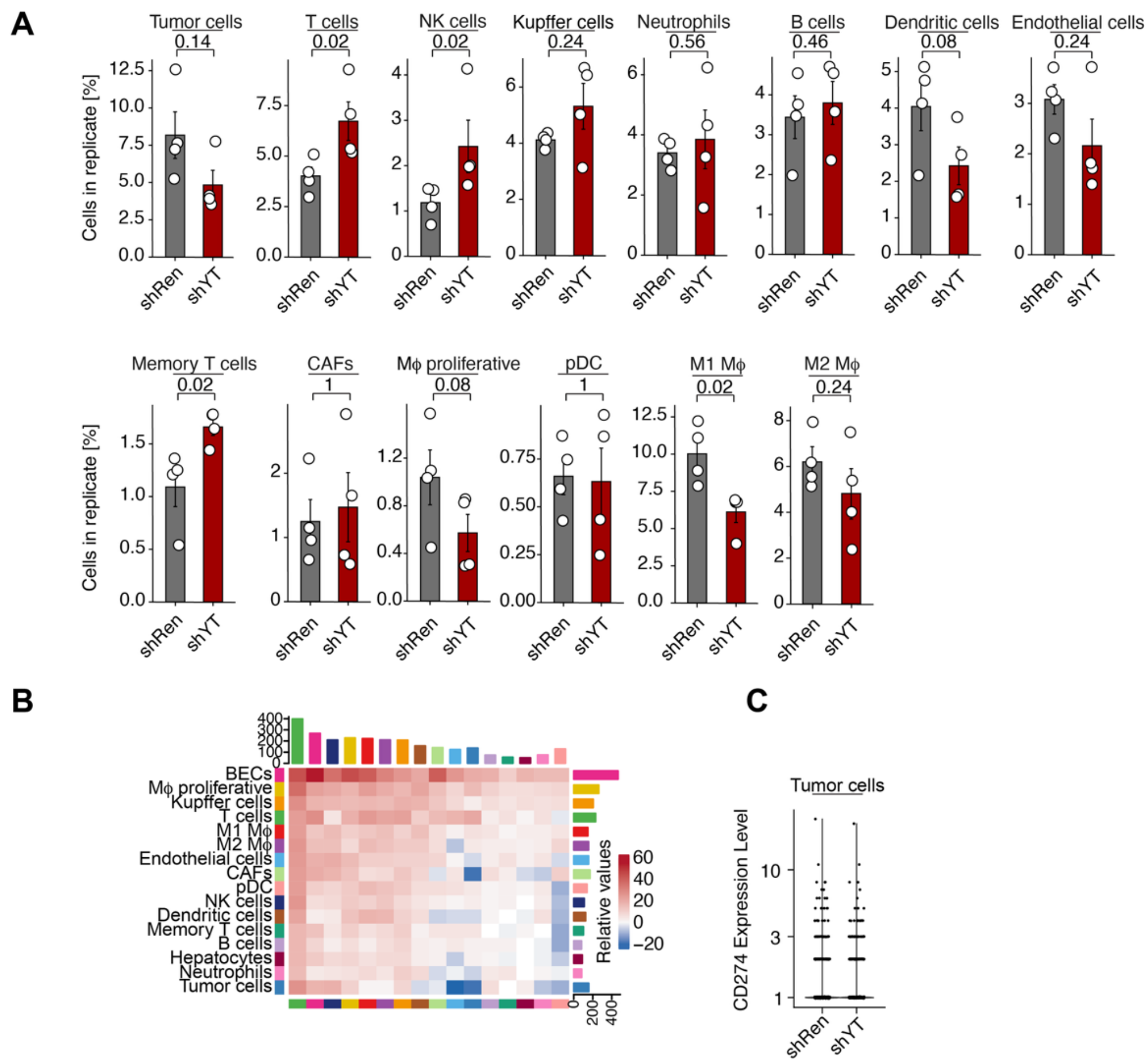

Supplemental Figure 7. Quantitative changes in the cellular composition of CCAs following acute YAP/TAZ depletion.

(**A**) Bar plots showing the proportion of each cell type across individual replicates from the scRNA-seq dataset comparing shRen (n = 4) with shYT (shYT#1, n = 2; shYT#2, n = 2). Statistical analysis: Welch's t test with Benjamini-Hochberg correction.

(**B**) Heatmap displaying the number of cell–cell interactions identified by CellChat analysis, stratified by cell type. Bar plots on the top and right indicate the total number of interactions sent and received, respectively, for each cell type.

(**C**) Violin plot illustrating the expression levels of *Cd274* (PD-L1) within the tumor cell cluster.

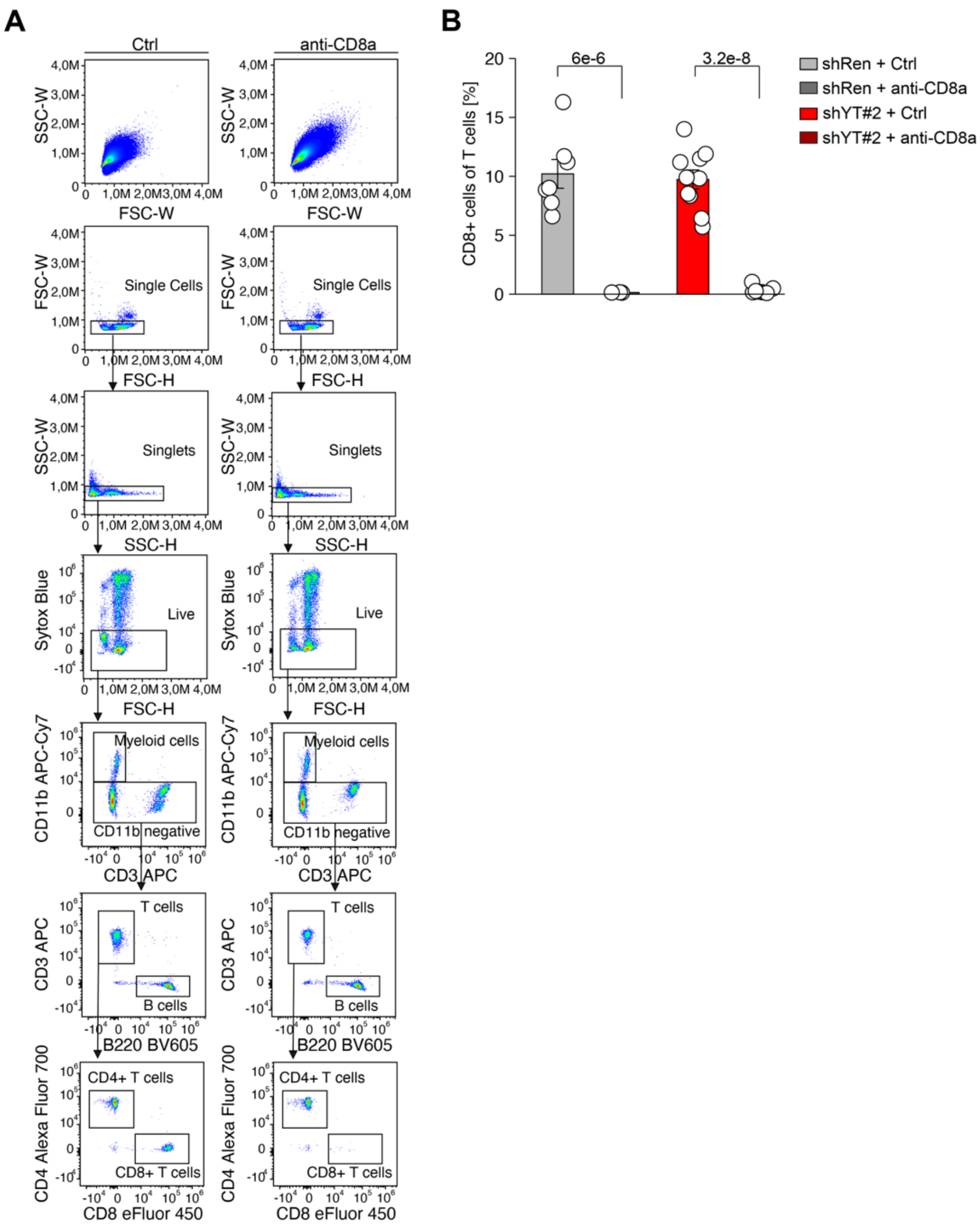

Supplemental Figure 8. Validation of CD8 T cell depletion.
(**A**, **B**) N-AKT–driven cholangiocarcinoma was induced via hydrodynamic tail vein injection (HDTVI), and CD8 T cells were depleted by intraperitoneal injection of monoclonal anti-CD8 antibodies on days 22 and 26 post-induction. Depletion efficiency was assessed on day 27. Representative FACS profiles and gating strategy are shown for peripheral blood from control (left) and anti-CD8–treated (right) mice. Quantification of $CD8^+$ T cells among live T cell populations (shRen Ctrl, n = 7; shRen anti-CD8, n = 3; shYT#2 Ctrl, n = 10; shYT#2 anti-CD8, n = 8).
Data represent mean ± SEM. Statistical analysis: one-way ANOVA with Tukey HSD post hoc test.

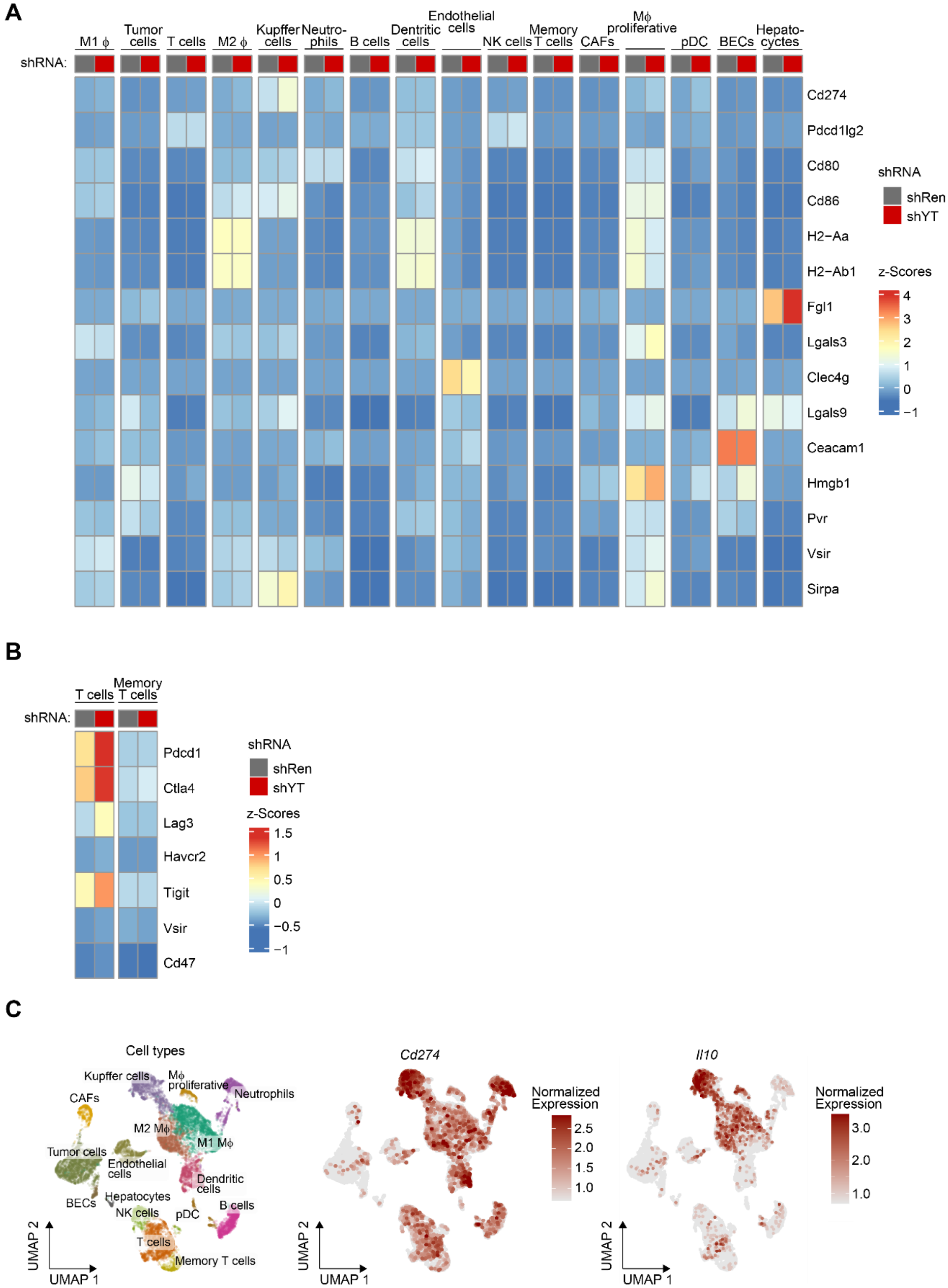
A
M1 ϕ
Tumor cells
T cells
M2 ϕ
Kupffer cells
Neutro-phils
B cells
Dentritic cells
Endothelial cells
NK cells
Memory T cells
CAFs
Mϕ proliferative
pDC
BECs
Hepato-cyctes
shRNA:
Cd274
Pdcd1lg2
Cd80
Cd86
H2−Aa
H2−Ab1
Fgl1
Lgals3
Clec4g
Lgals9
Ceacam1
Hmgb1
Pvr
Vsir
Sirpa
shRNA
shRen
shYT
z-Scores
4
3
2
1
0
−1
B
T cells
Memory T cells
shRNA:
Pdcd1
Ctla4
Lag3
Havcr2
Tigit
Vsir
Cd47
shRNA
shRen
shYT
z-Scores
1.5
1
0.5
0
−0.5
−1
C
Cell types
Kupffer cells
Mϕ proliferative
Neutrophils
CAFs
M2 Mϕ
M1 Mϕ
Tumor cells
Endothelial cells
Dendritic cells
Hepatocytes
BECs
NK cells
pDC
B cells
T cells
Memory T cells
UMAP 2
UMAP 1
Cd274
Normalized Expression
2.5
2.0
1.5
1.0
Il10
Normalized Expression
3.0
2.0
1.0

Supplemental Figure 9. YAP/TAZ depletion induces T cell exhaustion via immunosuppressive macrophages in the tumor microenvironment (TME).
(**A**) Heatmap showing the expression of immune checkpoint ligands following acute YAP/TAZ depletion across the indicated cell types identified by scRNA-seq.
(**B**) Heatmap showing the expression of exhaustion markers in T cells and Memory T cells after acute YAP/TAZ depletion.
(**C**) UMAP plot illustrating the expression of *Cd274* (PD-L1) and *Il10* within the TME.

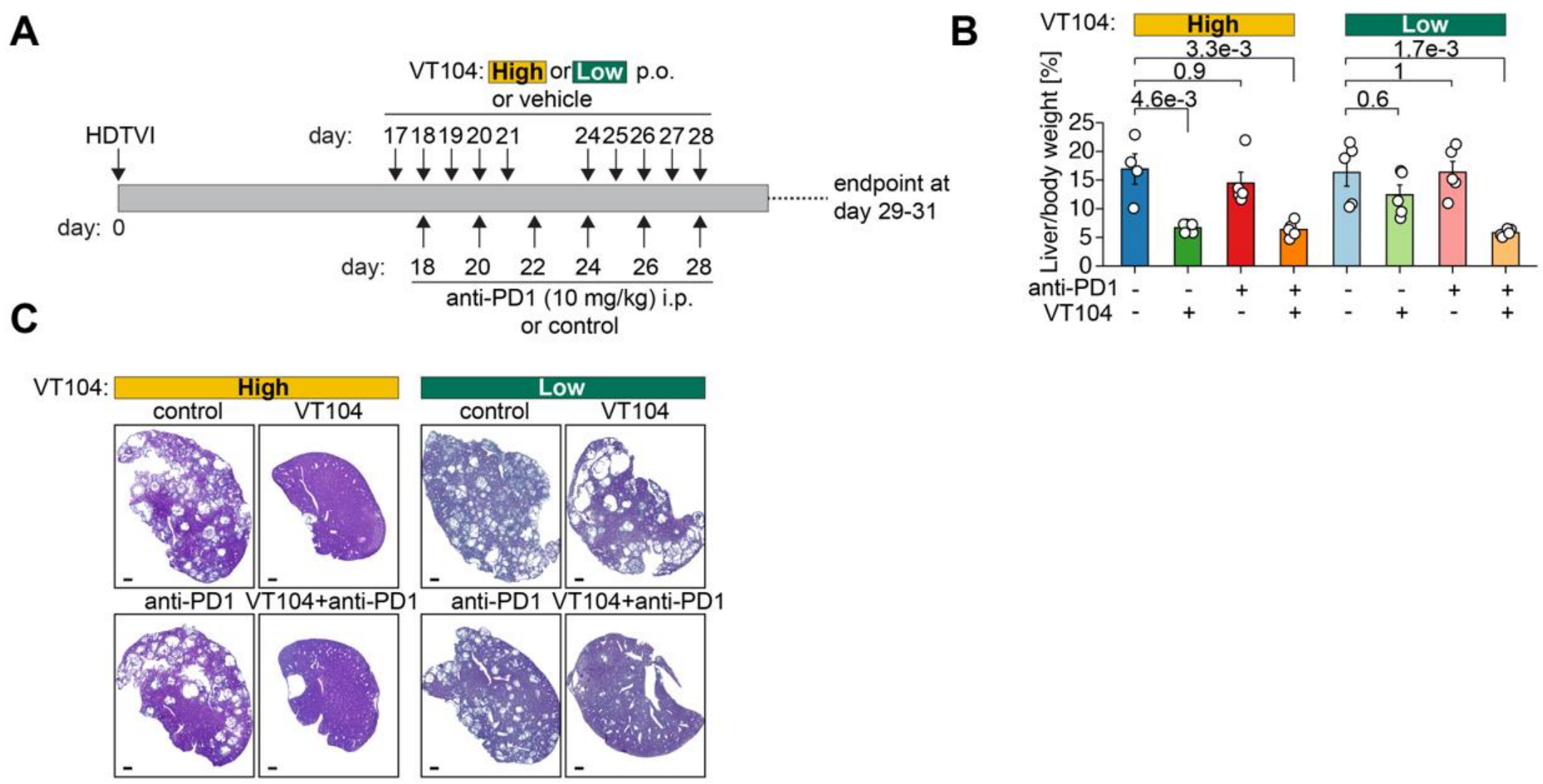

Supplemental Figure 10. Low-dose TEAD inhibitor treatment synergizes with anti-PD-1 therapy to reduce tumor burden in CCA.

(**A**) Experimental schematic: N-AKT–driven cholangiocarcinoma was treated with two cycles of VT104 (administered per os from days 17–21 and 24–28 post-induction) at high (10 mg/kg) or low (1 mg/kg) doses, in combination with anti–PD-1 antibody (10 mg/kg, intraperitoneal). Tumor burden was assessed on days 29–31.

(**B**) Liver-to-body weight ratios for each treatment group (n = 5 per group; Ctrl of high-dose VT104, n = 4).

(**C**) Representative H&E-stained liver sections from the indicated treatment conditions. Scale bars, 1 mm.

Data represent mean ± SEM. Statistical analysis: one-way ANOVA with Tukey HSD post hoc test.

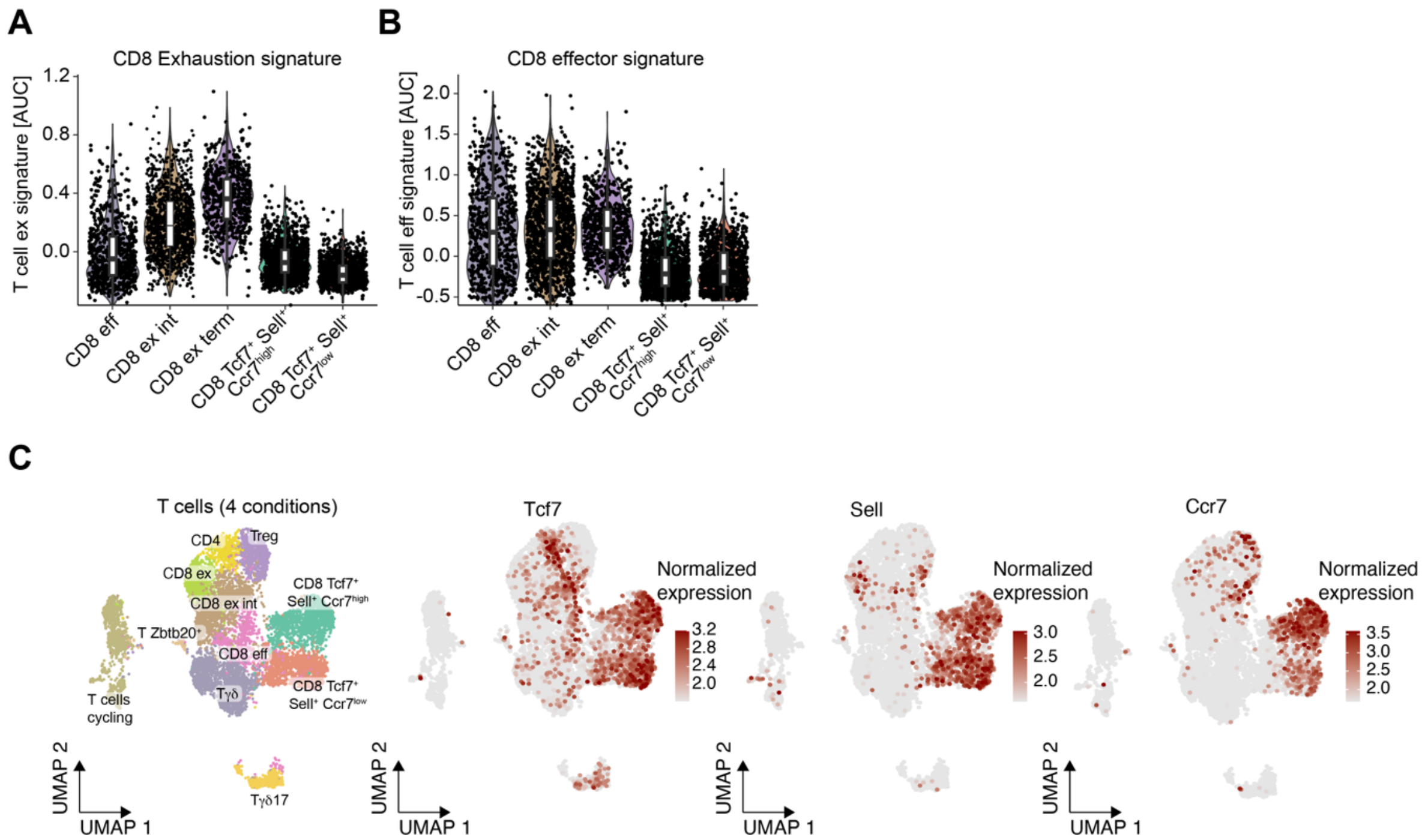

Supplemental Figure 11. T cell states after combining YAP/TAZ depletion and anti-PD-1 therapy. (**A**–**C**) Single-cell RNA-seq was performed following acute YAP/TAZ depletion in combination with anti–PD-1 therapy (see Fig. 6). T cell clusters were annotated based on expression of gene signatures associated with exhaustion (A), effector function (B), or naïve/memory-like markers (*Tcf7, Sell, Ccr7*) (C). Violin plots illustrate the distribution and density of the data. Boxplots show the median (center line), interquartile range (box), and whiskers extending to 1.5× the interquartile range. Data points beyond this range are shown as outliers.

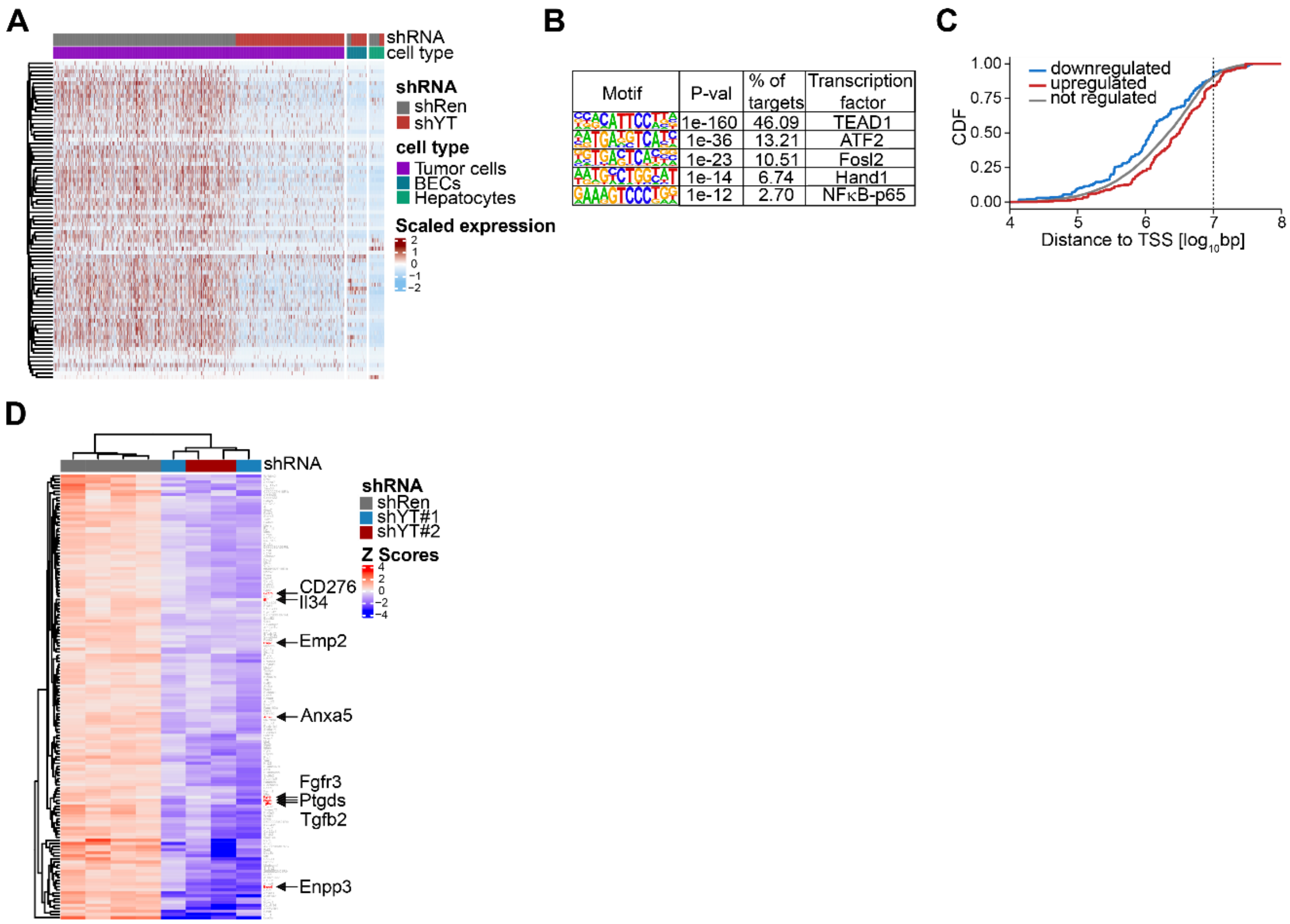

Supplemental Figure 12. YAP/TAZ drive an oncogenic transcriptional program in CCA tumor cells. (**A**) Heatmap of YAP/TAZ-dependent transcriptional changes in tumor cells, showing no effects on gene expression in normal biliary epithelial cells (BECs) and hepatocytes. Genes upregulated in control tumor cells and downregulated in shYAP/TAZ-depleted cells are not differentially expressed in BECs or hepatocytes.
(**B**) HOMER motif analysis of TEAD1 CUT&RUN peaks, indicating enriched motifs, associated p-values, percentage of total peaks, and predicted transcription factors.
(**C**) Cumulative distribution function of TEAD1 CUT&RUN peaks plotted by distance to the nearest transcription start site (TSS), stratified by gene regulation status in RNA-seq (see Supplementary Figure 5).
(**D**) Heatmap showing the expression of 144 YAP/TAZ-regulated candidate genes—identified by combined CUT&RUN and RNA-seq analyses—in purified cholangiocarcinoma cells following YAP/TAZ knockdown (shRen, n = 4; shYT#1, n = 2; shYT#2, n = 2).

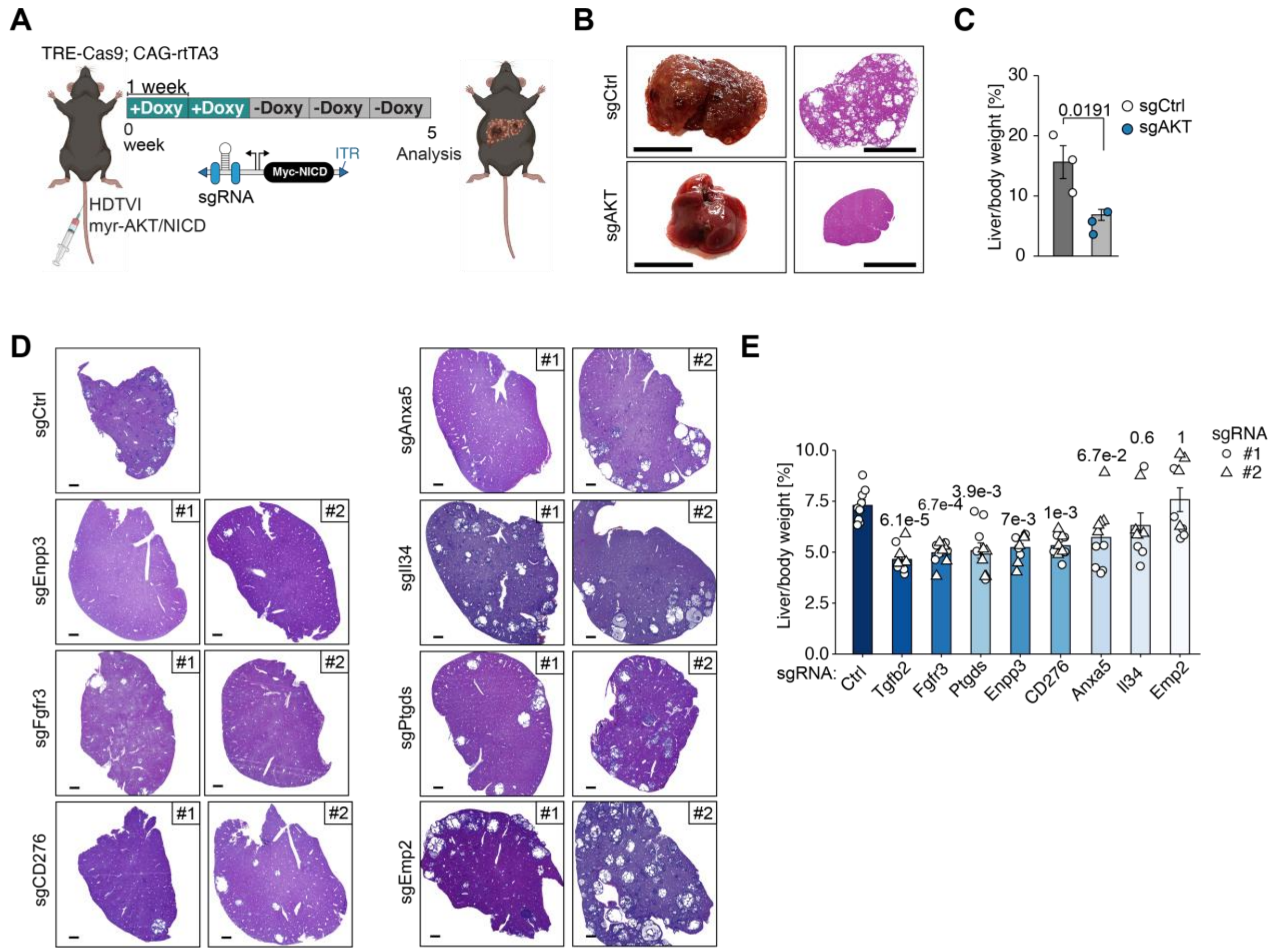

Supplemental Figure 13. Functional validation of *bona fide* YAP/TAZ target genes in CCA growth.
(**A**) Schematic depicting the use of an inducible Cas9 (iCas9) mouse model in conjunction with hydrodynamic tail vein injection (HDTVI) and a Myc-NICD-sgRNA plasmid to probe the relevance of YAP/TAZ targets in N-AKT–induced cholangiocarcinoma.
(**B**, **C**) Representative liver images (left) and H&E-stained liver sections (right) from mice expressing the indicated sgRNAs. Quantification of tumor area is shown. Scale bars, 1 cm (images), 500 μm (H&E). (n = 3 per group). Statistical analysis: Welch's t test with Benjamini-Hochberg correction.
(**D**) Representative H&E-stained liver sections from tumor-bearing mice expressing the indicated sgRNAs and treated following the regimen described in Figure 7F. Scale bars, 1 mm.
(**E**) Quantification of liver-to-body weight ratios in mice expressing the indicated sgRNAs (n = 5 per group; n = 4 for sgIl34#1 and #2; n = 9 for sgCtrl).
Statistical analysis: one-way ANOVA with Tukey HSD post hoc test.

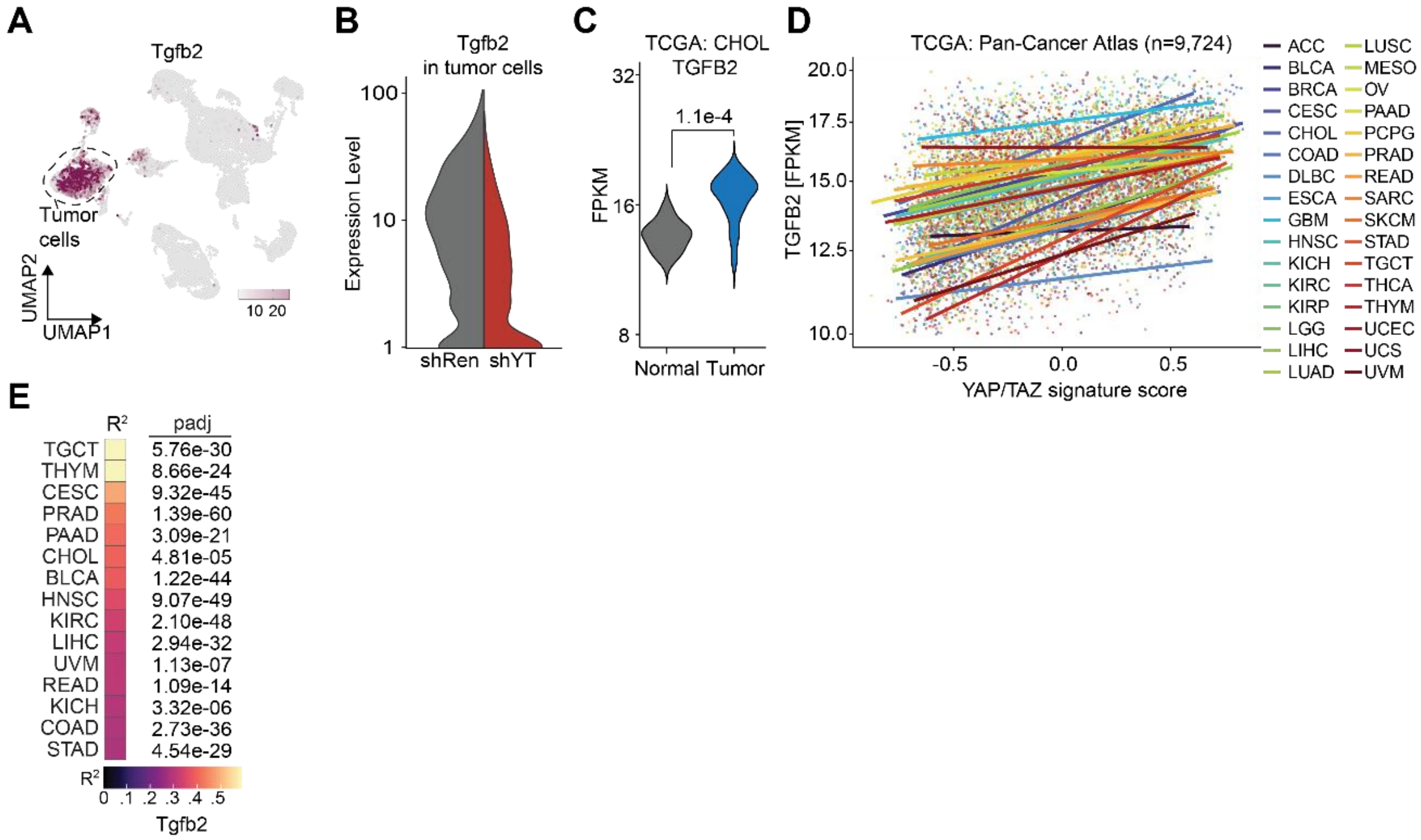

Supplemental Figure 14. *Tgfb2* is highly expressed in CCA tumor cells.
(**A**) UMAP plot showing *Tgfb2* expression across all cell types in the murine CCA scRNA-Seq data set. The tumor cell cluster is highlighted (shRen, n = 4; shYT#1, n = 2; shYT#2, n = 2).
(**B**) Violin plot illustrating differential expression of *Tgfb2* within the tumor cell cluster in control (shRen) and YAP/TAZ (shYT) depleted tumors following acute depletion of YAP/TAZ.
(**C**) Expression levels of *TGFB2* (measured in FPKM) in normal liver tissue (n = 9) and human cholangiocarcinoma patient samples (n = 36), based on data from the TCGA dataset.
(**D**) Correlation analysis between *TGFB2* expression and a conserved YAP transcriptional signature across different tumor types from the TCGA Pan-Cancer Atlas (n = 9,724).
(**E**) R^2 values and adjusted p-values for the top 15 cancer types showing the strongest correlations.

Supplemental Methods

Chemicals, enzymes and commercial assays

All chemicals, enzymes, and commercial assays used in this study are listed in Supplemental Tables 11 and 12.

RNA isolation and Quantitative RT-PCR

For RNA isolation from cultured cells, cells were lysed in TRIzol reagent (VWR). For tissue samples, the corresponding tissue was minced, resuspended in TRIzol, and mechanically homogenized using the Precellys Tissue Homogenizer (Bertin Technologies). Following lysis, samples were processed according to the manufacturer's protocol. Briefly, RNA was separated from DNA and proteins by phenol-chloroform extraction and subsequently precipitated using isopropanol. The concentration and purity of RNA were assessed using a NanoDrop 1000 spectrophotometer (Thermo Fisher Scientific), and samples were then processed for downstream applications.

For quantitative real-time PCR (RT-qPCR), 2 µg of total RNA was reverse transcribed into cDNA using M-MLV Reverse Transcriptase (Promega) following the manufacturer's instructions. The resulting cDNA was diluted 1:15, and RT-qPCR was performed using the InnuMIX qPCR MasterMix (Analytik Jena) in accordance with the manufacturer's specifications. Oligonucleotides used for RT-qPCR are listed in Supplemental Table 10.

Whole protein lysate and immunoblots

For whole-cell lysates, cells or tissues were lysed directly in ice-cold RIPA buffer (50 mM Tris-HCl pH 7.4, 150 mM NaCl, 1% NP-40, 0.5% sodium deoxycholate, 0.1% SDS)

supplemented with a protease inhibitor cocktail (Sigma-Aldrich) for 10 minutes on ice. For tissue samples, minced tissue was homogenized using the Precellys Tissue Homogenizer (Bertin Technologies) in RIPA buffer. Lysates were centrifuged at 16,000 × g for 10 minutes at 4°C, and the supernatant containing soluble proteins was collected.

Protein concentrations were determined using the BCA Protein Assay Kit. Samples were mixed with 5× SDS loading buffer and denatured for 5 minutes at 95°C. Equal amounts of protein (10–25 µg) were separated on 8% Bis-Tris gels for 2 hours at 120 V and transferred onto 0.45 µm PVDF membranes (Millipore) for 90 minutes at 250 mA.

Membranes were blocked in 5% non-fat dry milk prepared in TBS containing 0.25% Tween-20 (TBS-T) for at least 30 minutes at room temperature with constant agitation. Primary antibody incubation was performed overnight at 4°C in 5% BSA in TBS-T using 50 mL conical tubes under continuous rotation. Antibodies are listed in Supplemental Table 7. The following day, membranes were washed three times with TBS-T and incubated with horseradish peroxidase (HRP)-conjugated secondary antibodies for 1 hour at room temperature. After three additional washes with TBS-T, chemiluminescent signals were developed using Clarity ECL substrate (Bio-Rad) and imaged.

Generation of mouse embryonic fibroblasts (MEFs)

Mouse embryonic fibroblasts (MEFs) were isolated from pregnant mice at embryonic day E13.5. After removing the liver and head, embryos were minced and digested with 0.25% trypsin. Following 15 minutes of incubation, the embryos were pipetted up and down vigorously, and fresh trypsin was added. After additional 15 minutes, the digested embryos were plated in DMEM medium containing 10% fetal bovine serum and 1%

Penicillin/Streptomycin. For immortalization, MEFs were infected with a retrovirus containing an shRNA targeting p19 ARF.

Peripheral blood analysis

To confirm efficient depletion of $CD8^+$ T cells, 25 µL of peripheral whole blood was incubated for 30 minutes with fluorochrome-conjugated antibodies (1:100 dilution in FACS buffer) targeting CD11b (myeloid marker), CD3 (T cell marker), B220 (B cell marker), CD8α (CD8 T cells), and CD4 (CD4 T cells) (Supplemental Table 7). Following antibody incubation, red blood cells were lysed, and cells were washed twice with FACS buffer. Dead cells were excluded by staining with Sytox Blue. Samples were analyzed on a Cytek Spectral Flow Cytometer. The gating strategy is shown in Supplemental Fig. 8.

Cholangiocarcinoma cell (CCA) isolation

Tumor-bearing livers were minced and digested in 15 mL of 1× PBS containing 7.5 mg Pronase, 7.5 mg Collagenase P, and 1.5 mg DNase I using a C Tube (Miltenyi Biotec) and the gentleMACS Octo Dissociator (Miltenyi) for 45 minutes at 100 rpm and 37 °C. Following digestion, the cell suspension was filtered through a 100 µm cell strainer and washed with an appropriate volume of Advanced DMEM/F12 (AdDMEM/F12) medium. Red blood cell lysis was performed using the Red Blood Cell Lysis Solution (Miltenyi, #130-094-183), followed by debris removal (Miltenyi, #130-109-398) and dead cell removal (Miltenyi, #130-090-101) according to the manufacturer's protocols. Cells were then incubated with Fc receptor blocking reagent (Miltenyi) for 10 minutes on ice. Tumor cells were labeled with anti-EpCAM-APC antibody (eBioscience, #17-5791-82; 1:100) for 30 minutes on ice. After washing, cells were incubated with anti-APC microbeads (Miltenyi) for an additional 30 minutes. The cell suspension was then loaded onto LS

columns (Miltenyi) and processed according to the manufacturer's instructions. $EpCAM^+$ cells were eluted and further processed for downstream applications.

Biliary epithelial cell (BEC) isolation

Liver tissues were finely minced and processed following the procedure described under the Cholangiocarcinoma Cell Isolation section. Red blood cells were removed using red blood cell lysis buffer (Miltenyi). To enrich for non-parenchymal cells, hepatocytes were depleted via low-speed centrifugation (5 minutes at 30 ×g). The remaining cell suspension was incubated with Fc receptor blocking reagent (Miltenyi Biotec) for 10 minutes on ice to prevent nonspecific antibody binding. BECs were identified and labeled using an anti-EpCAM-APC antibody (eBioscience, #17-5791-82; dilution 1:100). To exclude hematopoietic and erythroid lineage cells, samples were simultaneously stained with the following lineage markers (LIN) conjugated to APC-Cy7: TER-119 (erythroid cells; BD Biosciences, #560509, 1:100), CD11b (myeloid cells; BD Biosciences, #557657, 1:100), CD45 (pan-leukocyte marker; BD Biosciences, #557659, 1:100). Staining was performed for 30 minutes on ice in the dark. After two washes with FACS buffer (PBS + 2% FBS), samples were filtered through a 40 μm mesh and analyzed on a BD cell sorter. BECs were defined as $EpCAM^+/LIN^-$ events. The gating strategy is shown in Supplementary Figure 2.

Generation of mouse cholangiocarcinoma organoids

Cholangiocarcinoma (CCA) organoids were established from N-AKT tumor–bearing livers 25–35 days after induction via hydrodynamic tail vein injection (HDTVI). Following

isolation, tumor-containing liver tissue was mechanically minced and enzymatically digested in 15 mL of 1× PBS supplemented with 7.5 mg pronase, 7.5 mg collagenase P, 1.5 mg DNase I, and 2 mg/mL gentamicin. Digestion was performed using C Tubes and the gentleMACS Octo Dissociator (Miltenyi Biotec) for 25 minutes at 37 °C and 100 rpm. The resulting cell suspension was filtered through a 100 µm cell strainer and washed with Advanced DMEM/F12 (AdDMEM/F12). Red blood cells were lysed using Red Blood Cell Lysis Solution. Organoids were enriched from single cells by repeated brief centrifugation steps. Organoids were resuspended on ice in Cultrex Reduced Growth Factor Basement Membrane Extract Type 2 and seeded as 10 µL domes in 48-well plates. After polymerization as hanging drops for at least 30 minutes at 37 °C, domes were overlaid with complete growth medium consisting of Advanced DMEM/F12 supplemented with 1% penicillin/streptomycin, 1× B27, 1× N2, 1.25 µM N-acetylcysteine, 50 ng/mL human EGF, 1 µg/mL R-spondin, 100 ng/mL Noggin, 10 µM Y-27632 dihydrochloride, and 1 µM SB203580. For initial organoid establishment, the medium was additionally supplemented with 10 ng/mL Wnt3a for the first two weeks of culture. Cultures were passaged weekly at a ratio of 1:2 to 1:3. For passaging, Cultrex domes were washed once with ice-cold PBS and incubated with Cultrex Organoid Harvesting Solution for 90 minutes on ice with gentle agitation. Organoids were collected using pre-wetted pipette tips (0.1% BSA in PBS) into pre-coated 15 mL tubes and washed once with ice-cold PBS. Subsequently, organoids were dissociated with TrypLE Express for 2 minutes at 37 °C, washed, and pelleted. The cell pellet was then resuspended in Cultrex matrix and replated as 10 µL domes in 48-well plates.

Atomic force microscopy

For atomic force microscopy, liver samples were embedded in optimal cutting temperature (OCT) compound and stored at −80°C until sectioning. Tissue sections (10 µm thickness) were prepared using a Leica cryostat, mounted onto 18 × 18 mm poly-L-lysine–coated coverslips, and stored at −80°C until analysis. The elasticity (Young's Modulus) of the sectioned tissue samples was determined in PBS by force-distance spectroscopy using a NanoWizard 3 atomic force microscope (AFM; JPK Bruker). Colloidal AFM probes equipped with a 10 µm silicon dioxide sphere at the end of the cantilever (CP-sq-SCONT-SiO; sQube) with a spring constant of 0.01 N/m and a resonance frequency of 11 kHz were used. The cantilevers were calibrated before the measurements. During the experiments, the approach speed was set to 0.5 µm/s, and a maximum force of 0.5 nN was applied. For each sample, four 20x20 µm regions were probed using a 5 x 5 grid. Between consecutive force curves, the cantilever was retracted by 3 µm at the same speed.

The force-distance curves were analyzed using the JPK Data Processing software (version 6.4) and fitted with the Hertz model. An indentation depth of 1500nm was considered for all fits. Analysis of lower indentations consistently yielded values for the Young's (elasticity) modulus below 100 Pa, suggesting that the sample surfaces were highly hydrated and that the relevant tissue layers had not yet been reached.

Incomplete approach curves or curves showing an unexpected sudden cantilever deflection were excluded from data evaluation. The Poisson ratio was set to 0.5. To illustrate the Young's modulus distribution, a violin plot was generated followed by a statistical analysis using the Kruskal-Wallis test and a subsequent Dunn's post hoc test.

Luciferase Assay

For luciferase assays, 1×10^5 Lenti-X cells (Takara, #632180) were seeded into 24-well plates. The following day, cells were transfected using PEI Max with 100 ng of the respective pGL4.23 reporter construct, 5 ng of pCMV-Renilla plasmid for normalization, and increasing amounts of pLEGO-FLAG-YAP5SA (0, 5, 50, and 500 ng). Six hours after transfection, the medium was replaced. At 30 hours post-transfection, cells were washed twice with 1×PBS and lysed directly in 100 µL passive lysis buffer. For measurement, 10 µL of cell lysate was mixed with 100 µL of either Firefly substrate (25 mM Gly-Gly, 15 mM K_2HPO_4, 4 mM EGTA pH 8, 15 mM $MgSO_4$, 75 µM Luciferin, 2 mM ATP, 1 mM DTT) or Renilla substrate (25mM Na_4PPi, 10 mM NaAc, 15 mM EDTA, 500 mM Na_2SO_4, 500 mM NaCl, 50 µM phenyl-benzothiazole, 4 µM (benzyl-)coelenterazine) and luminescence 560nm (firefly) and 480nm (renilla) was quantified using a Tecan plate reader.

Supplemental Table 1. Transcriptional changes following acute depletion of YAP/TAZ in purified cholangiocarcinoma (CCAs) cells.

This table lists differentially expressed genes after acute YAP/TAZ depletion in EpCAM enriched tumor cells. For each gene, the table provides the Entrez ID, Ensembl ID, gene symbol, base mean expression, log2 fold change, p-value, and adjusted p-value (padj).

Supplemental Table 2. Upregulated genes following acute depletion of YAP/TAZ in T cells.

This table presents genes that are significantly upregulated in T cells upon acute YAP/TAZ depletion, as identified by single-cell RNA sequencing (scRNA-seq) analysis shown in Figure 4D. These genes were subsequently used to stratify TCGA cholangiocarcinoma (CCA) patients into high- and low-expression groups, as shown in Figure 4E. For each gene, the table includes the gene symbol, p-value, average log2 fold change, adjusted p-value (padj), and the corresponding Seurat cluster.

Supplemental Table 3. T cell exhaustion gene signature.

This table lists the genes used to define the T cell exhaustion signature analyzed in Figure 5E.

Supplemental Table 4. T cell effector gene signature.

This table lists the genes used to define the T cell effector signature applied in the classification of T cells shown in Figure 6F and 6H.

Supplemental Table 5. YAP/TAZ-driven transcriptional program sustaining oncogenic features of CCA.

This table lists genes that are specifically downregulated in cholangiocarcinoma (CCA) tumor cells following YAP/TAZ depletion but not affected in non-malignant biliary epithelial cells (BECs) or hepatocytes. These data correspond to the heatmap shown in Supplemental Figure 12A.

Supplemental Table 6. Bona fide YAP/TAZ target genes identified in CCA.

This table lists high-confidence YAP/TAZ target genes in cholangiocarcinoma (CCA), defined by the presence of a TEAD1 binding peak within a 10^7 bp window near the transcription start site (TSS) (CUT&RUN analysis) and significant downregulation upon YAP/TAZ depletion (log2 fold change < -1; adjusted p-value $< 1\times10^{-3}$, bulk-RNAseq).

Supplemental Table 7. List of Antibodies

Name	Source	Identifier	Application, concentration
Anti-mouse PD-1 (cloneRMP1-14)	Bio X Cell	BE0146	i.p. injection 10 mg/kg
Anti-mouse CD8 (clone YTS169)	Self produced		i.p. 500 µg per mouse
SOX9	Millipore	5535	IHC, 1:200
CK19	DSHB	TROMA-III	IHC, 1:15
CD3	Abcam	ab16669	IHC, 1:150
CD8	Cell Signaling	98941S	IHC, 1:200
anti-EpCAM-APC	eBioscience	17-5791-82	FACS, 1:100
anti-Mouse TER-119/Erythroid Cells-APC-Cy7	BD Bioscience	560509	FACS, 1:100
anti-CD11b-APC-Cy7	BD Bioscience	557657	FACS, 1:100
anti-Mouse CD45-APC-Cy7	BD Bioscience	557659	FACS, 1:100
H3K27Ac	Abcam	Ab4729	CUT&RUN, 1:100
H3K4me1	Cell Signaling	5326	CUT&RUN, 1:100
TEAD1	Cell Signaling	12292	CUT&RUN, 1:100
VINCULIN	Sigma-Aldrich	V9131	WB, 1:10 000
YAP (D8H1X)	Cell Signaling	14074	WB, 1:1000<br>IHC, 1:200<br>CUT&RUN, 1:100
Goat Anti-Rabbit Immunoglobulins/HRP	Agilent	P044801-2	WB, 1:5000<br>IHC, 1:1000
Goat Anti-Mouse Immunoglobulins/HRP	Agilent	P044701-2	WB, 1:5000<br>IHC, 1:1000
TotalSeq-B0301 (Hashtag 1)	Biolegend	155831	scRNAseq, 1µg
TotalSeq-B0302 (Hashtag 2)	Biolegend	155833	scRNAseq, 1µg
TotalSeq-B0303 (Hashtag 3)	Biolegend	155835	scRNAseq, 1µg
TotalSeq-B0304 (Hashtag 4)	Biolegend	155837	scRNAseq, 1µg

Supplemental Table 8. List of 70mer oligos for sgRNA cloning

This table contains the sequences of 70-nucleotide (70mer) single-stranded DNA oligonucleotides used for the cloning of single guide RNAs (sgRNAs) into the psBbi-Myc-NICD-sgRNA vector. Each oligo includes necessary flanking sequences for compatibility with Gibson Assembly via SapI cutting site.

Gene	Sequence 5'-3`	
	70-mer	sgRNA target sequence
sgCtrl	ATCTTGTGGAAAGGACGAAACACCGATGTTGCAGTTCGGCTCGATGTTTTAGAGCTAGAAATAGCAAGTT	ATGTTGCAGTTCGGCTCGAT
sgCD276#1	ATCTTGTGGAAAGGACGAAACACCGCGCGTCCGAGTAACCGACGAGTTTTAGAGCTAGAAATAGCAAGTT	CGCGTCCGAGTAACCGACGA
sgCD276#2	ATCTTGTGGAAAGGACGAAACACCGGCGCGTCCGAGTAACCGACGGTTTTAGAGCTAGAAATAGCAAGTT	GCGCGTCCGAGTAACCGACG
sgEmp2#1	ATCTTGTGGAAAGGACGAAACACCGGCCGGTCAGCTCATTGATCTGTTTTAGAGCTAGAAATAGCAAGTT	GCCGGTCAGCTCATTGATCT
sgEmp2#2	ATCTTGTGGAAAGGACGAAACACCGTTGGCGCCGGTCTGTATAGAGTTTTAGAGCTAGAAATAGCAAGTT	TTGGCGCCGGTCTGTATAGA
sgEnpp3#1	ATCTTGTGGAAAGGACGAAACACCGATATTACGAGTCCTGATTCGGTTTTAGAGCTAGAAATAGCAAGTT	ATATTACGAGTCCTGATTCG
sgEnpp3#2	ATCTTGTGGAAAGGACGAAACACCGACGTGCAATTTATTCCGTTGGTTTTAGAGCTAGAAATAGCAAGTT	ACGTGCAATTTATTCCGTTG
sgFgfr3#1	ATCTTGTGGAAAGGACGAAACACCGAGCCGGGCAATCCGGACAAGGTTTTAGAGCTAGAAATAGCAAGTT	AGCCGGGCAATCCGGACAAG
sgFgfr3#2	ATCTTGTGGAAAGGACGAAACACCGGGTATAGTTGCCACGATCGGGTTTTAGAGCTAGAAATAGCAAGTT	GGTATAGTTGCCACGATCGG
sgIl34#1	ATCTTGTGGAAAGGACGAAACACCGGACCTTACAGGCTACCTTCGGTTTTAGAGCTAGAAATAGCAAGTT	GACCTTACAGGCTACCTTCG
sgIl34#2	ATCTTGTGGAAAGGACGAAACACCGTCTTGGGATCCTACTTGACGGTTTTAGAGCTAGAAATAGCAAGTT	TCTTGGGATCCTACTTGACG
sgPtgds#1	ATCTTGTGGAAAGGACGAAACACCGATTGAGGCCGCCTTCTGTGGGTTTTAGAGCTAGAAATAGCAAGTT	ATTGAGGCCGCCTTCTGTGG
sgPtgds#2	ATCTTGTGGAAAGGACGAAACACCGCAGAGCGTACTCGTCATAGTGTTTTAGAGCTAGAAATAGCAAGTT	CAGAGCGTACTCGTCATAGT
sgAnxa5#1	ATCTTGTGGAAAGGACGAAACACCGCATTGCTTCGGGATGTCAACGTTTTAGAGCTAGAAATAGCAAGTT	CATTGCTTCGGGATGTCAAC
sgAnxa5#2	ATCTTGTGGAAAGGACGAAACACCGTAGGCATCGTAGAGTCGTGAGTTTTAGAGCTAGAAATAGCAAGTT	TAGGCATCGTAGAGTCGTGA

sgTgfb2#1	ATCTTGTGGAAAGGACGAAACACCGCGAGG AGTACTACGCCAAGGGTTTTAGAGCTAGAAA TAGCAAGTT	CGAGGAGTACTACGCCAAGG
sgTgfb2#2	ATCTTGTGGAAAGGACGAAACACCGTGGAT CAGTTTATGCGCAAGGTTTTAGAGCTAGAAA TAGCAAGTT	TGGATCAGTTTATGCGCAAG

Supplemental Table 9. Oligos used for ATACseq library amplification

Primer	Sequence 5'-3`	
	sequence	barcode
Ad1.1	AATGATACGGCGACCACCGAGATCTACACTAGATCGCTCGTCGGCA GCGTCAGATGTGTAT	TAGATCGC
Ad1.2	AATGATACGGCGACCACCGAGATCTACACCTCTCTATTCGTCGGCAG CGTCAGATGTGTAT	CTCTCTAT
Ad1.3	AATGATACGGCGACCACCGAGATCTACACTATCCTCTTCGTCGGCAG CGTCAGATGTGTAT	TATCCTCT
Ad1.4	AATGATACGGCGACCACCGAGATCTACACAGAGTAGATCGTCGGCA GCGTCAGATGTGTAT	AGAGTAGA
Ad1.5	AATGATACGGCGACCACCGAGATCTACACGTAAGGAGTCGTCGGCA GCGTCAGATGTGTAT	GTAAGGAG
Ad1.6	AATGATACGGCGACCACCGAGATCTACACACTGCATATCGTCGGCAG CGTCAGATGTGTAT	ACTGCATA
Ad2.1	CAAGCAGAAGACGGCATACGAGATTCGCCTTAGTCTCGTGGGCTCG GAGATGTG	TAAGGCGA
Ad2.2	CAAGCAGAAGACGGCATACGAGATCTAGTACGGTCTCGTGGGCTCG GAGATGTG	CGTACTAG

Supplemental Table 10. Oligos used for qRT-PCR

Primer	Sequence 5'-3`	
	Forward	Reverse
Yap1	TGAGATCCCTGATGATGTACCAC	TGTTGTTGTCTGATCGTTGTGAT
Wwtr1	GAAGGTGATGAATCAGCCTCTG	GTTCTGAGTCGGGTGGTTCTG
Ctgf	GGGCCTCTTCTGCGATTTC	ATCCAGGCAAGTGCATTGGTA
Cyr61	CTGCGCTAAACAACTCAACGA	GCAGATCCCTTTCAGAGCGG

Supplemental Table 11. Commercial assays

Name	Source	Identifier
innuMix qPCR DSGreen Standard	Analytik Jena	845-AS-1300200
NEBNext® Ultra II RNA Library Prep kit for Illumina	NEB	E7770
NEBNext® Poly(A) mRNA Magnetic Isolation Module	NEB	E7490
NEBNext Multiplex Oligos for Illumina	NEB	E6440
Chromium Next GEM Single Cell 3' Reagent Kits v3.1	10x Genomics	PN-1000268
GEXSCOPE Single Cell RNA Library Kit V2	Singleron	SD: 4180011/ 4180012
NEBNext Ultra II DNA Library Prep Kit for Illumina	NEB	E7645
Illumina Tagment DNA Enzyme and Buffer Small Kit	Illumina	20034197
DNA Clean & Concentrator-5 (Uncapped)	ZYMO Research	D4004

NEBNext High-Fidelity 2X PCR Master Mix	NEB	M0541
Click-iT™ EdU Cell Proliferation Kit	Thermo Fisher Scientific	C10337
Red Blood Cell Lysis Solution	Miltenyi	130-094-183
Debris Removal Solution	Miltenyi	130-109-398
Dead Cell Removal Kit	Miltenyi	130-090-101
RNeasy Mini Kit	Quiagen	74104
RNase-Free DNase Set	Quiagen	79254

Supplemental Table 12. Chemicals and enzymes

Name	Source	Identifier
0.25% Trypsin	Thermo Fisher Scientific	25200056
37% Formaldehyde	Carl Roth	4979.1
AdDMEM/F-12	Thermo Fisher Scientific	12634010
Antigen Retrieval Buffer 100x Citrate pH 6	abcam	Ab64236
Antigen Retrieval Buffer 100x Tris-EDTA, pH 9	abcam	Ab93684
BioMag Plus concanavalin A	Polysciences	86057-10
Bicine	Sigma-Aldrich	215589
Bis-Tris	Carl Roth	9140.3
Bovine Serum Albumin (BSA)	Carl Roth	8076.1
CaCl2	Carl Roth	CN92.1
Chlorobutanol	Sigma-Aldrich	112054
Clarity Max Western ECL Substrate	Bio Rad	1705062
Collagenase P	Sigma Aldrich	11213857001
Cultrex Reduced Growth Factor Basement Membrane Extract Type 2	R&D Systems	3533-005-02
Cultrex Organoid Harvesting Solution	R&D Systems	3700-100-01
Digitonin	Sigma-Aldrich	D141
Direct Red 80 (Sirius Red)	Sigma Aldrich	365548
DMEM (GlutaMAX™)	Thermo Fisher Scientific	61965059
doxycycline-containing chow (625 mg/kg doxycycline hyclate	Sniff	326652
DNaseI	Roche	05952077103
DpnII	NEB	R0543M
EDTA	Merck	1.08452.1000
EGTA	Carl Roth	3054.3
Eosin G-solution	Carl Roth	X883.2
EpCAM-APC antibody	eBioscience	17-5791-82
Fc receptor blocking reagent, mouse	Miltenyi	130-092-575
Fetal Bovine Serum	Gibco	F7524-500ML
Gentamicin	Thermo Fisher Scientific	15710064
Glycerol	Carl Roth	3783.2
Glycine	Carl Roth	3908.3
GlycoBlue™ Coprecipitant	Invitrogen	AM9515
Hematoxylin solution (Meyer)	Carl Roth	T865.1
Hematoxylin solution A (Weigert)	Carl Roth	X906.1
Hematoxylin solution B (Weigert)	Carl Roth	X907.1
HEPES	Sigma-Aldrich	H3375
Hoechst 33343	Sigma-Aldrich	B2261
Hydrogen peroxide solution (30%)	Honeywell	216763
Immobilon-P Transfer Membrane (0.45 µm)	Merck	IPVH00010
Isopropanol	Carl Roth	T902.1
KCl	Carl Roth	6781.2

M-MLV Reverse Transcriptase	Promega	M1705
MgCl2	Carl Roth	KK36.3
Methanol	Carl Roth	4627.2
MnCl2	Carl-Roth	0276.1
NP-40	Sigma Aldrich	74385
Passive lysis buffer	Promega	E194
PEI Max	Polysciences	24765-1
Penicillin/Streptomycin	Thermo Fisher Scientific	15140122
peqGOLD TriFast™	VWR	30-2010
Phusion Hot Start II DNA Polymerase	Thermo Fisher Scientific	F549L
Picric acid solution (1.3 % in H2O)	Sigma Aldrich	P6744
Potassium dihydrogen phosphate (K2HPO4)	Carl Roth	P018.1
Powdered Milk	Carl Roth	T145.2
Pronase	Sigma Aldrich	10165921001
Protease Inhibitor Cocktail (PIC)	Sigma-Aldrich	P8340
Proteinase K	Thermo Fisher Scientific	EO0491
Random primer p(dN)6	Sigma-Aldrich	11034731001
Ringer-Lactate solution	WDT	-
RNase A	Quiagen	1032722
ROTI®Phenol	Carl Roth	0038.2
SB203580	MedChemExpress	HY-10256
SDS Pellets	Carl Roth	CN30.2
Sodium bisulfite (NaHSO4)	Sigma-Aldrich	243973
Sodium chloride (NaCl)	Carl Roth	P029.3
Sodium-deoxycholate	Sigma Aldrich	D6750
Spermidine	Sigma-Aldrich	S0266
Tris	Carl Roth	A411.2
Tris-HCl	Sigma-Aldrich	T3253
TrypLE Express	Gibco	12604013
Tween-20	Carl Roth	9127.1
T4-Ligase	Thermo Fisher Scientific	EL0013
Vector® ImmPACT DAB Peroxidase (HRP)Sub.	Vector Laboratories	SK-4105
Y-27632 dihydrochloride (iROCK)	Torcis	1254

Supplemental Table 13. Plasmid constructs

**Name**	**Backbone**	**Insert**
**Retrovirus constructs**		
pRS-Puro sh p19 Arf	pRetro-Super-Puro	Sh p19Arf (mouse)
**HDTVI plasmids**		
pCMV(CAT)T7-SB100	pCMV	SB100
pSBbi-w/oPuro-Myr-AKT-HA	pSBbi-w/oPuro	Myr-AKT-HA
pSBbi-w/oPuro-Myr-AKT-HA_IRES_TGFB2	pSBbi-w/oPuro	Myr-AKT-HA; IRES; TGFB2
pSBbi-w/oPuro-Myc-NICD	pSBbi-w/oPuro	Myc-NICD
psBbi-Myc-NICD-sgRNA	pSBbi-w/oPuro	Myc-NICD; sgRNA
