# Supplementary material for: Extracellular matrix reprogramming by the YAP/TAZ/TGF-β2 axis drives immune exclusion in cholangiocarcinoma models
Source: J Clin Invest. 2026 Jul 2;136(17):e201579. doi: 10.1172/JCI201579 (PMC13528937; doi:10.1172/JCI201579)

Full unedited blot for Figure 1B lower panel - Vinculin

uncropped

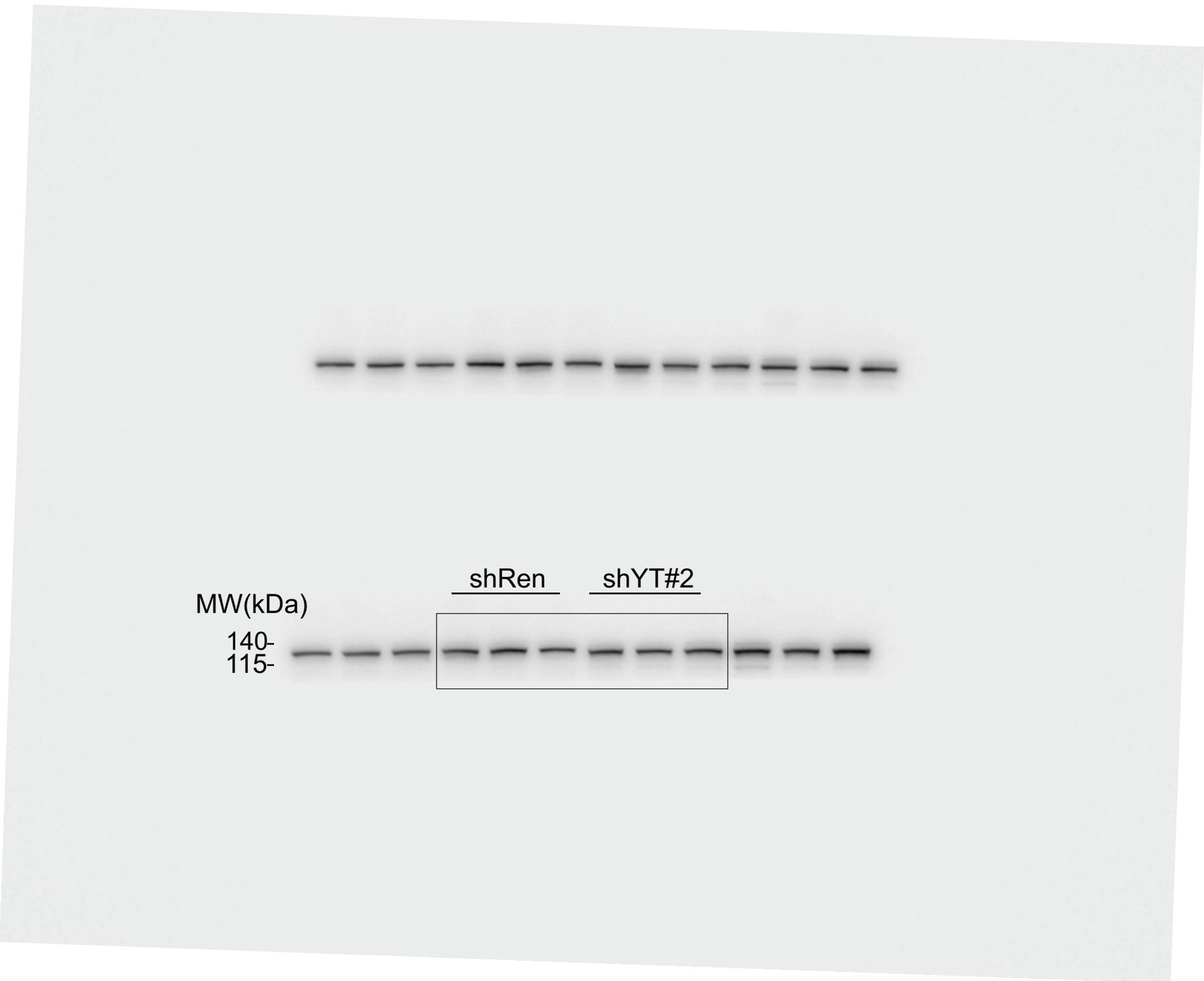

Merged

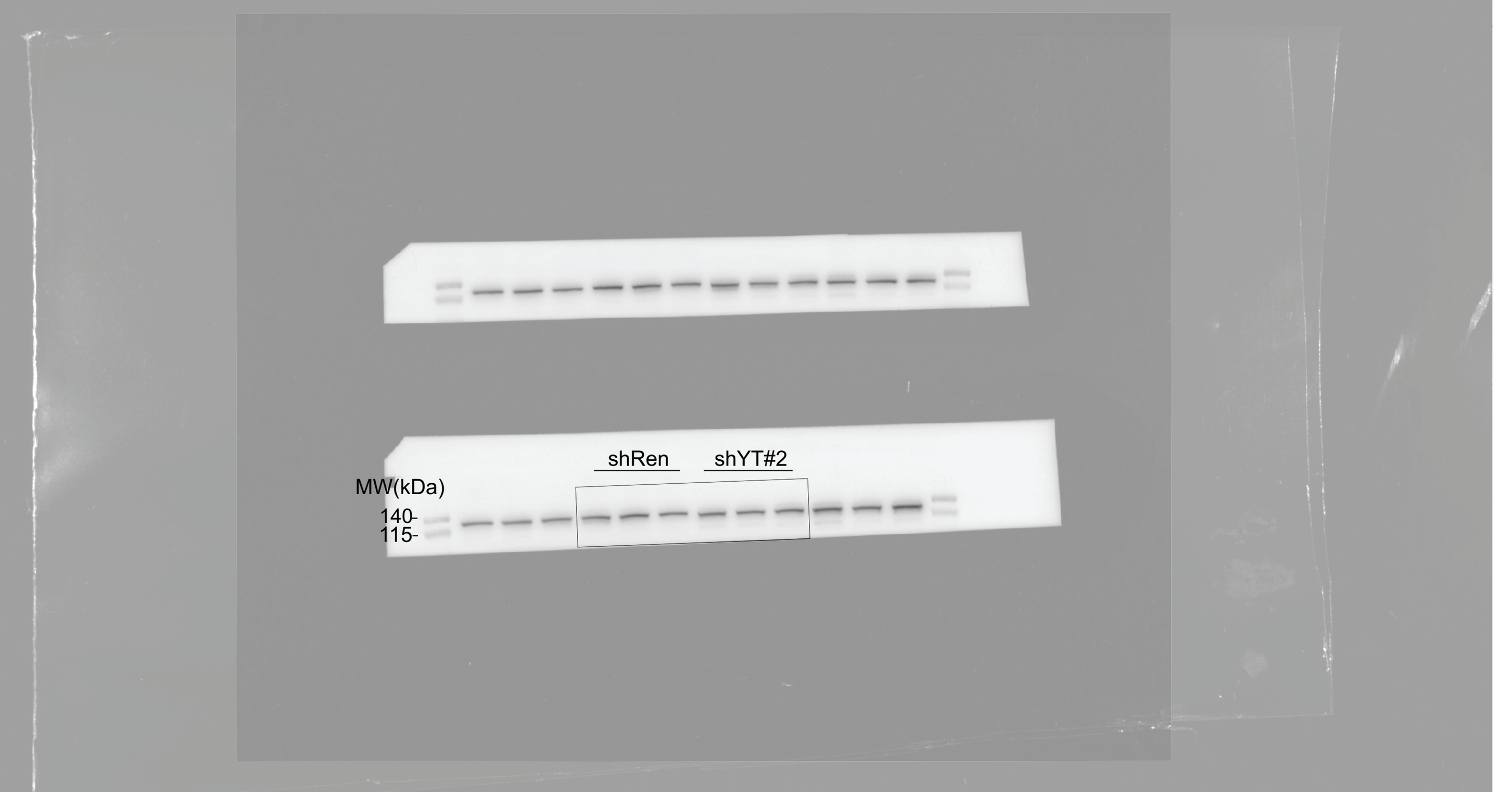

Full unedited blot for Figure 1B lower panel- TAZ

uncropped

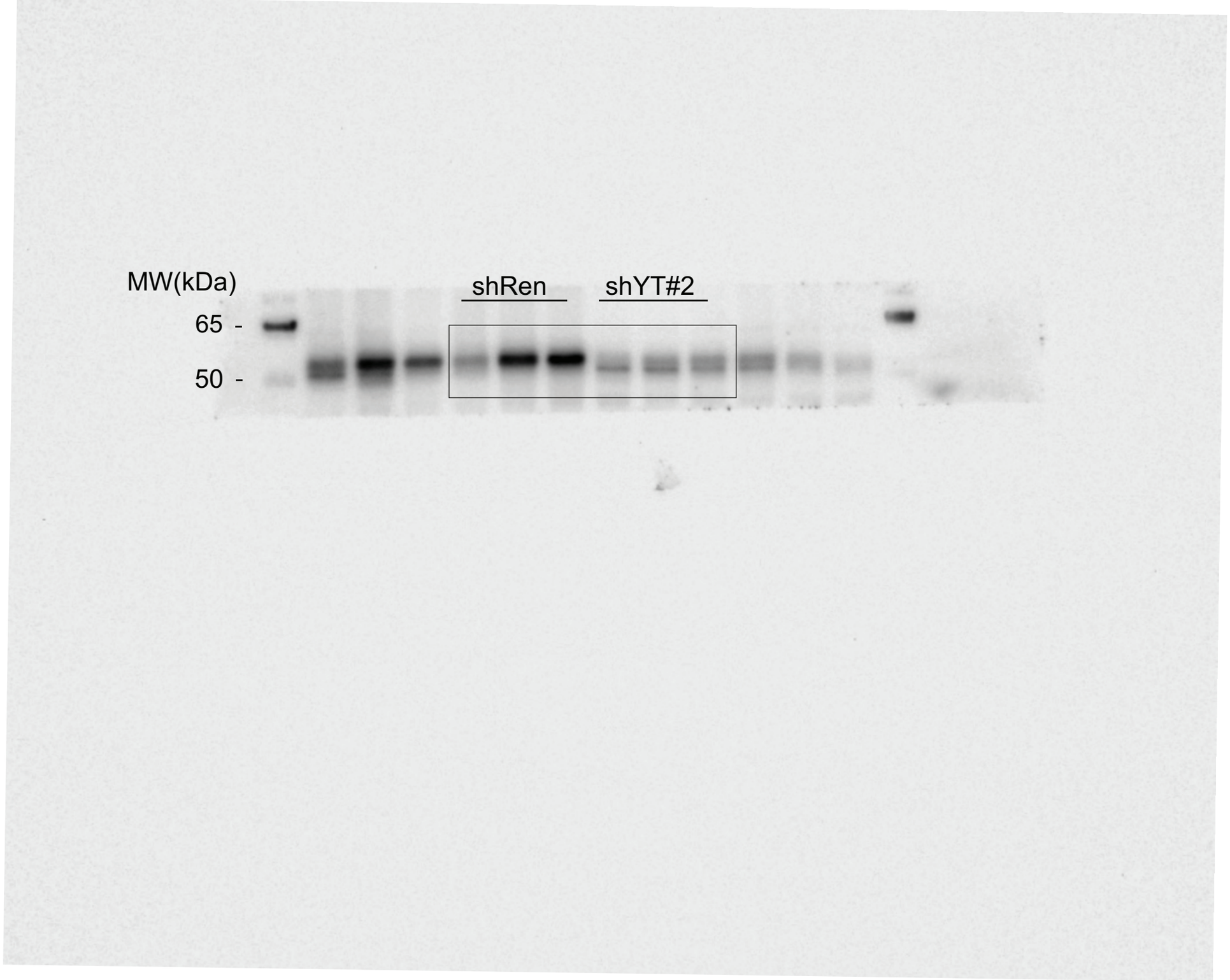

Merged

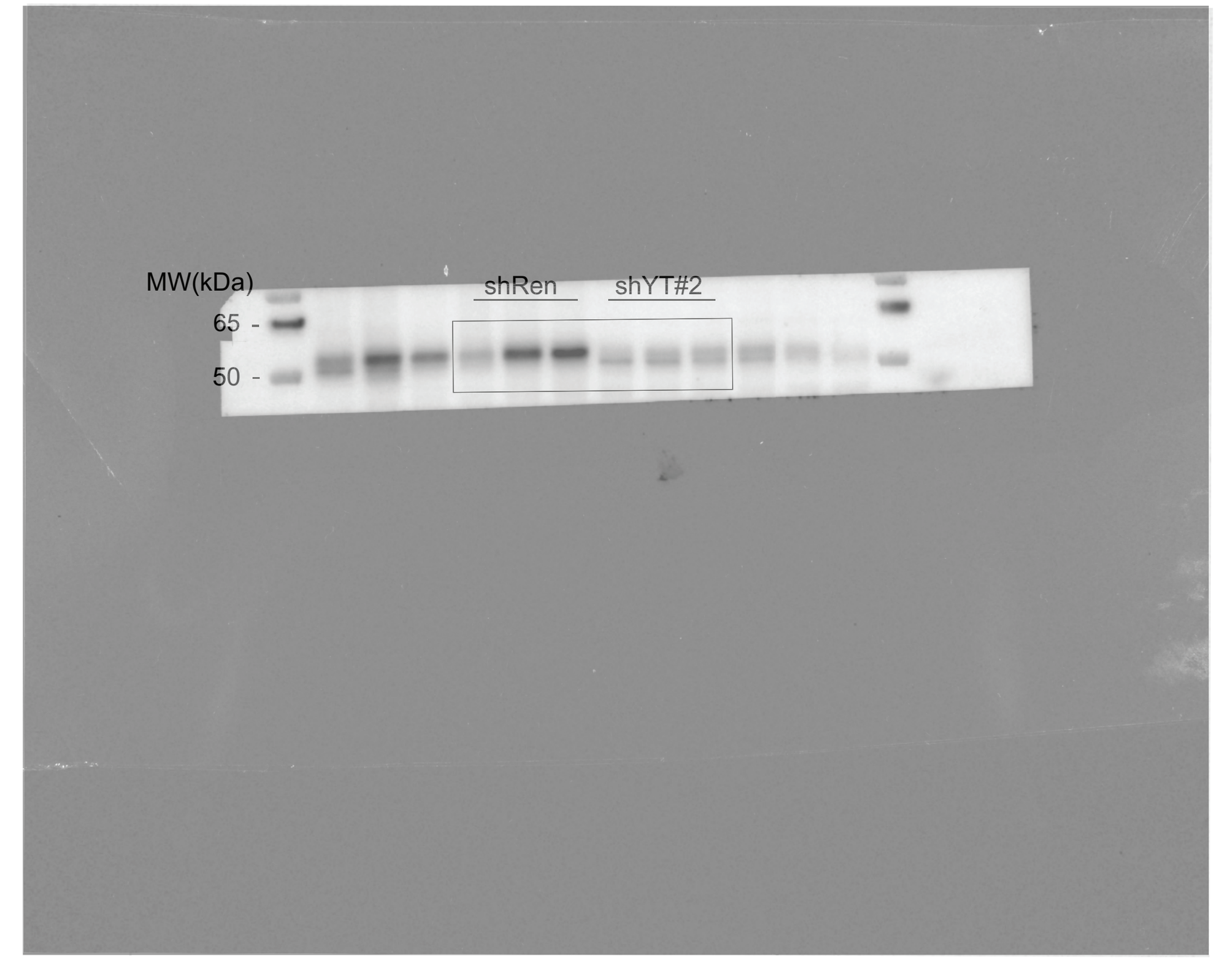

Full unedited blot for Figure 1B lower panel - YAP

uncropped

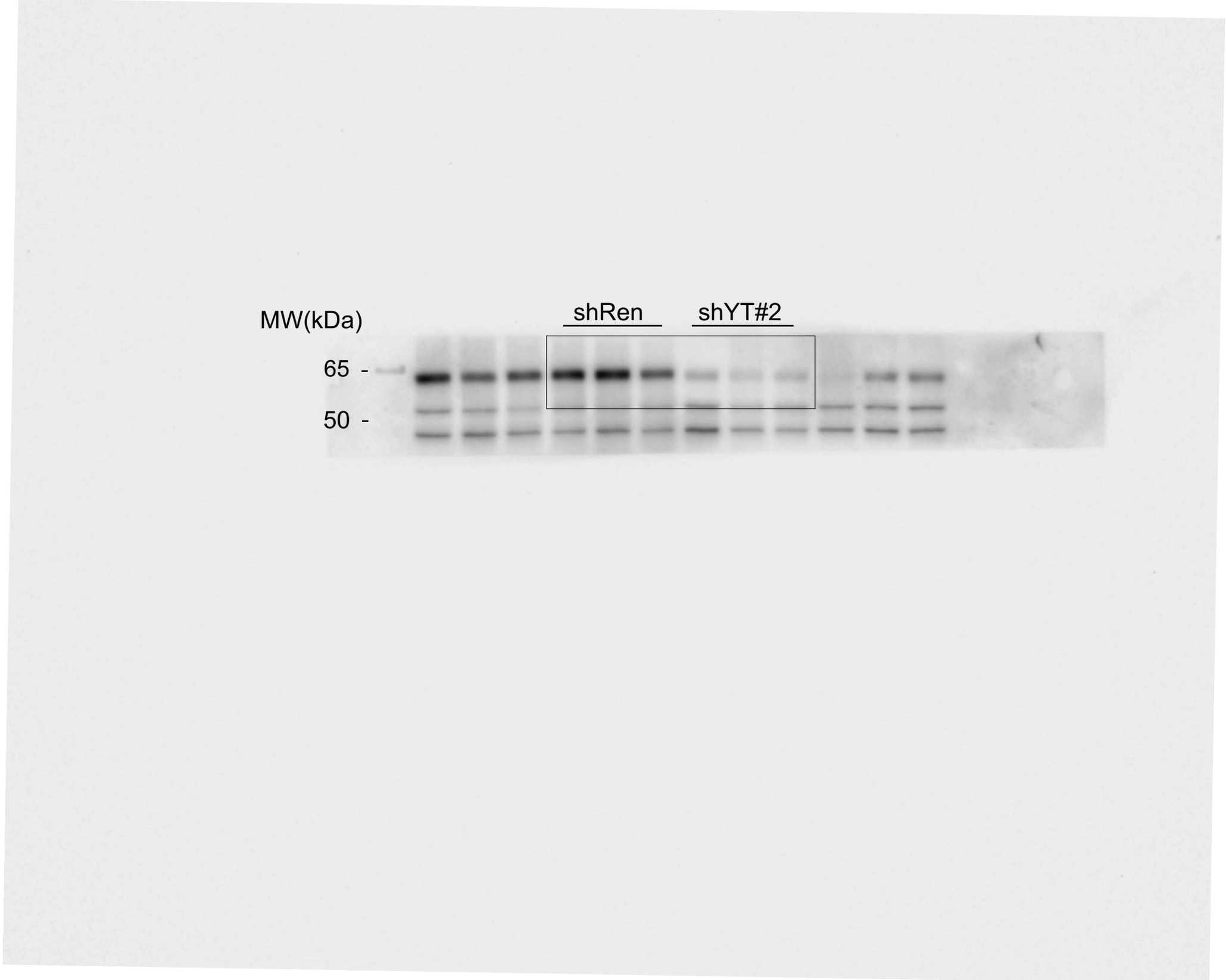

Merged

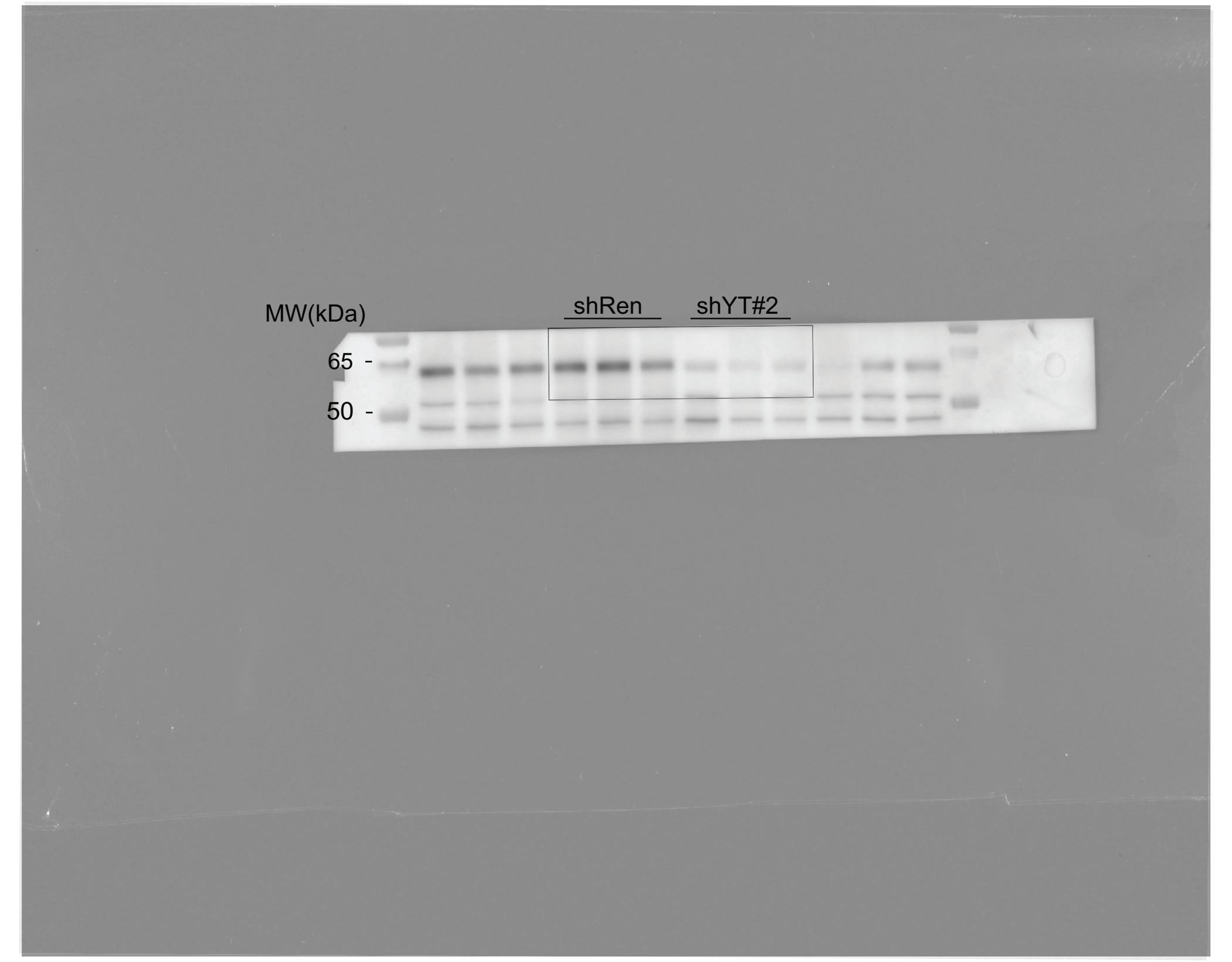

Full unedited blot for Figure 1B lower panel - Vinculin

uncropped

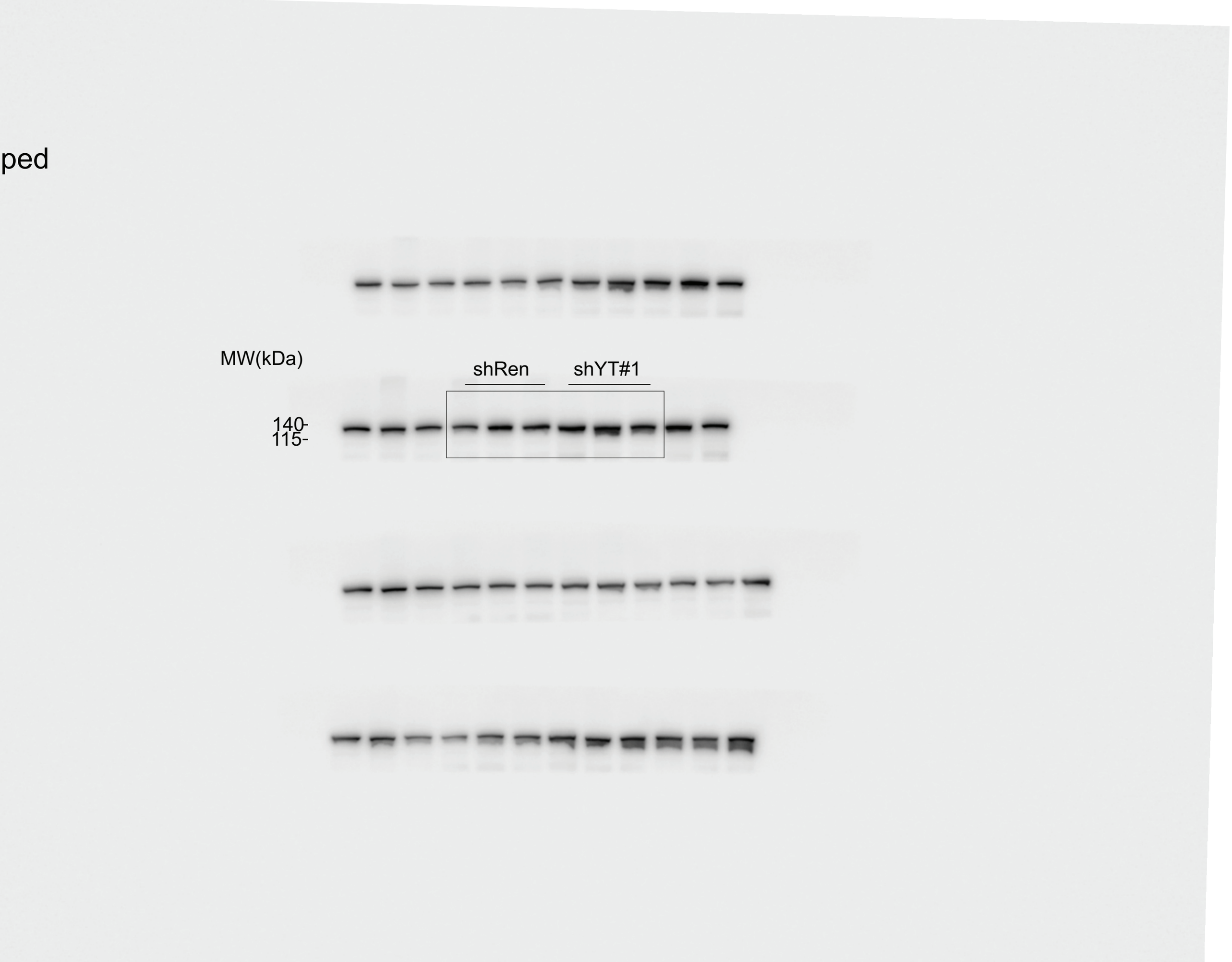

Merged

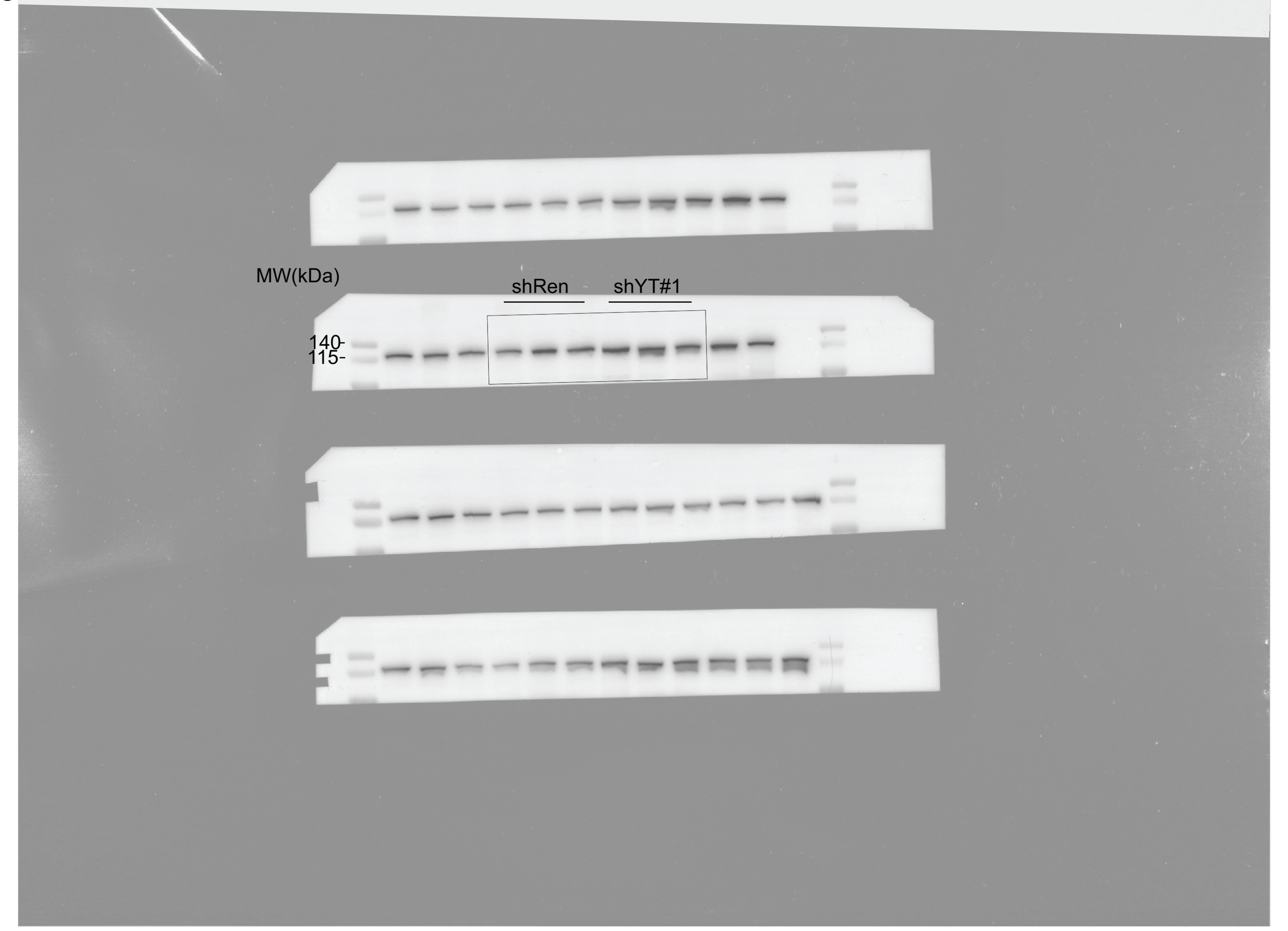

Full unedited blot for Figure 1B lower panel - TAZ

uncropped

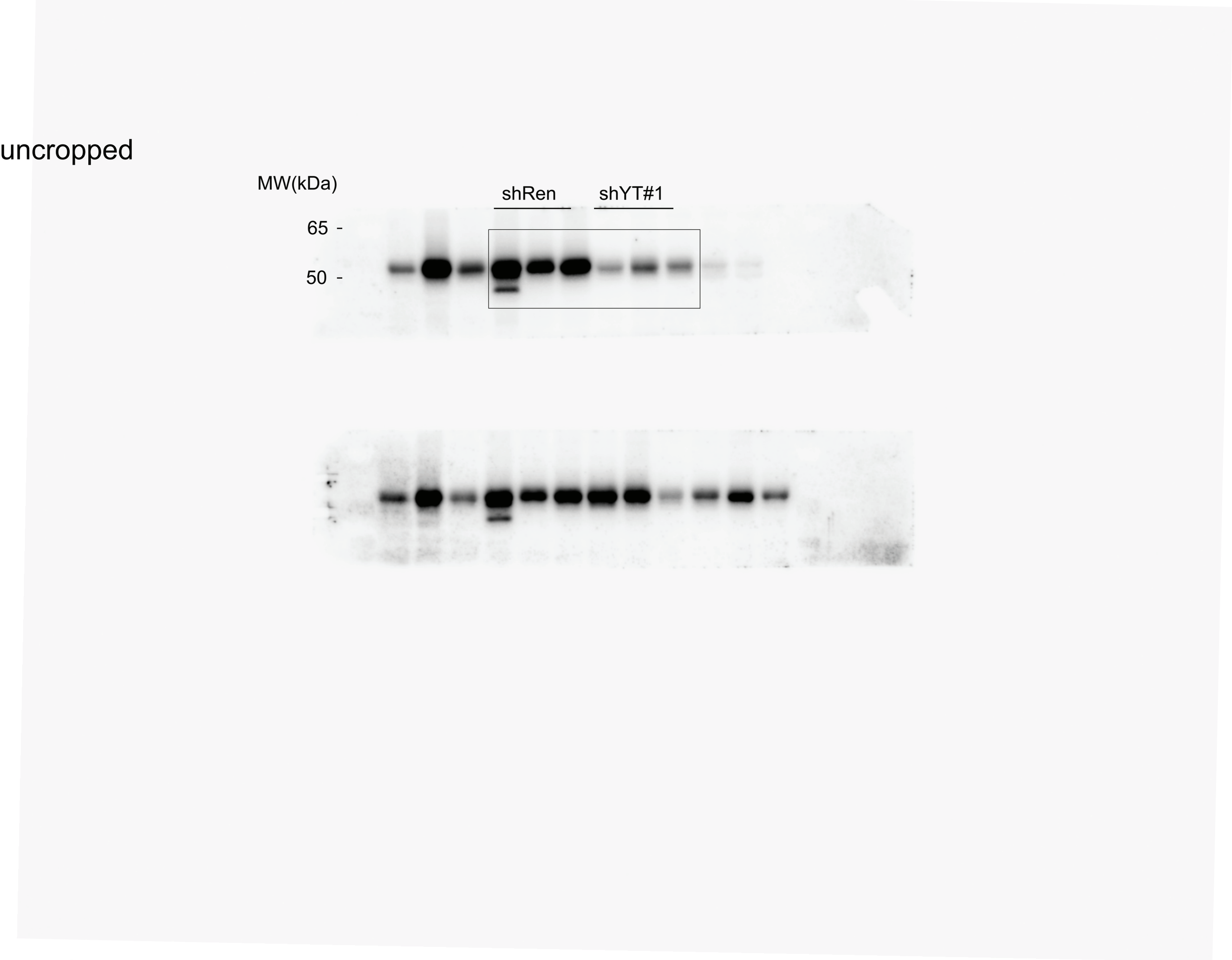

Merged

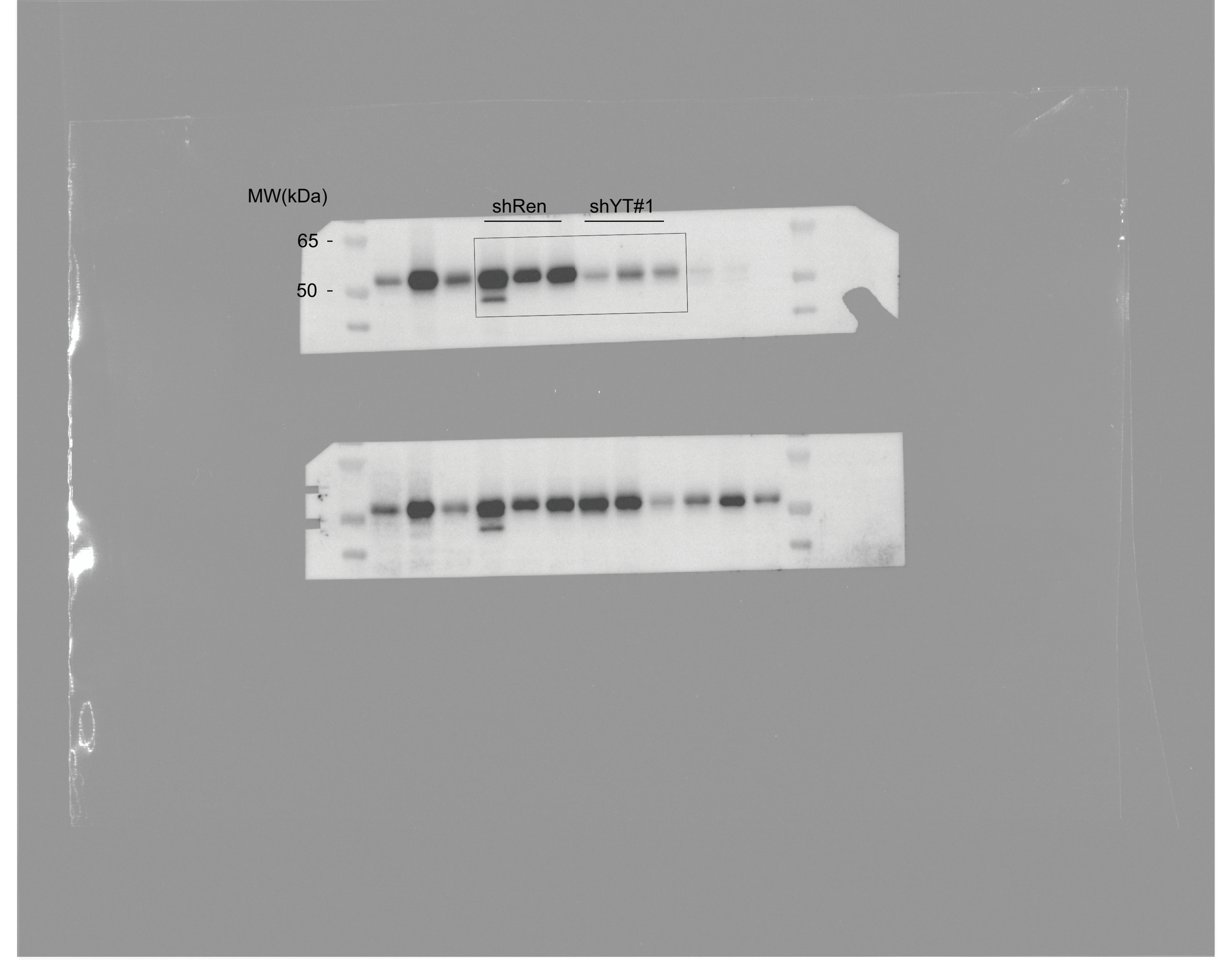

Full unedited blot for Figure 1B lower panel - YAP

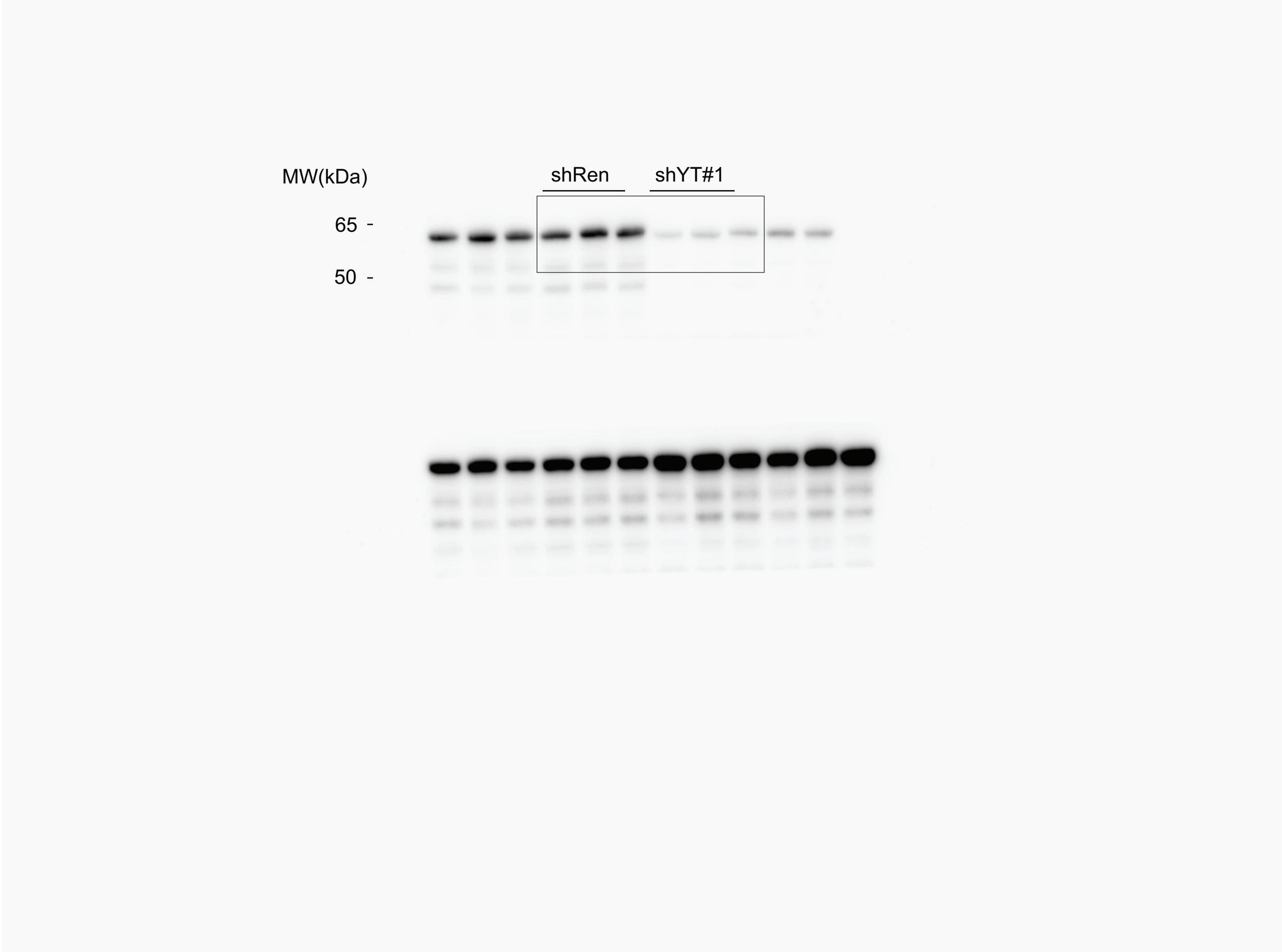

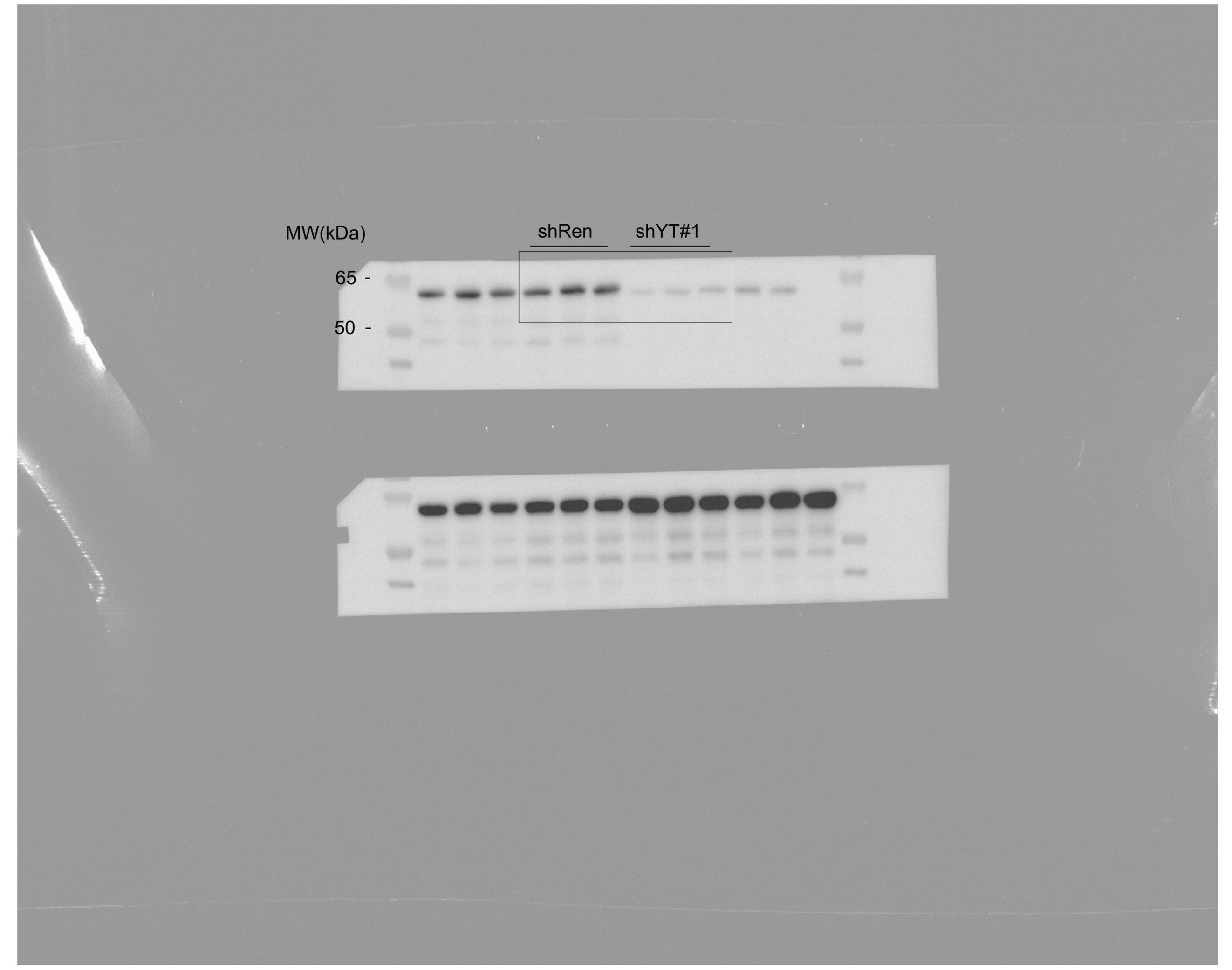

Full unedited blot for Figure 8F lower panel - left panel

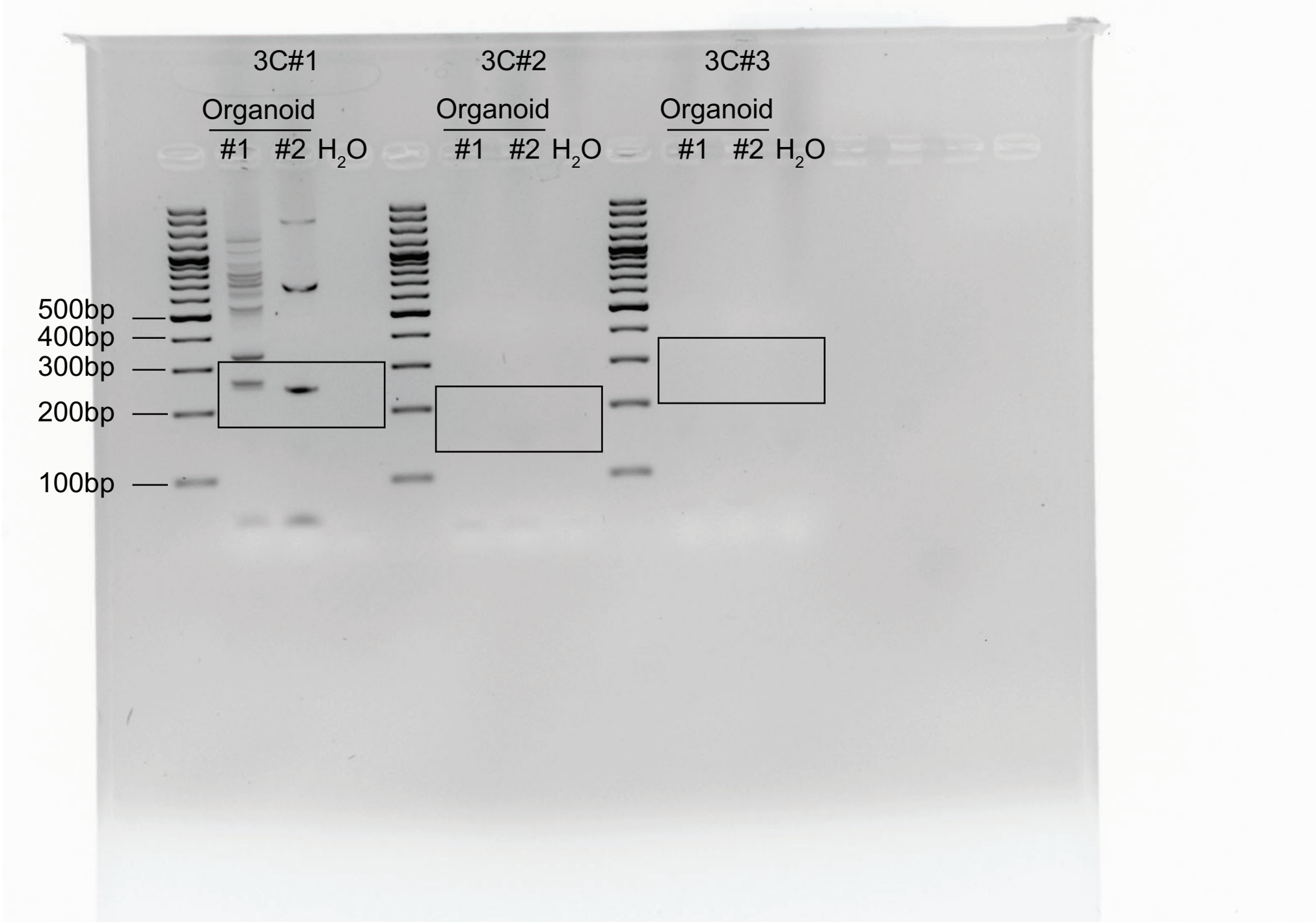

Full unedited blot for Figure 8F - right panel

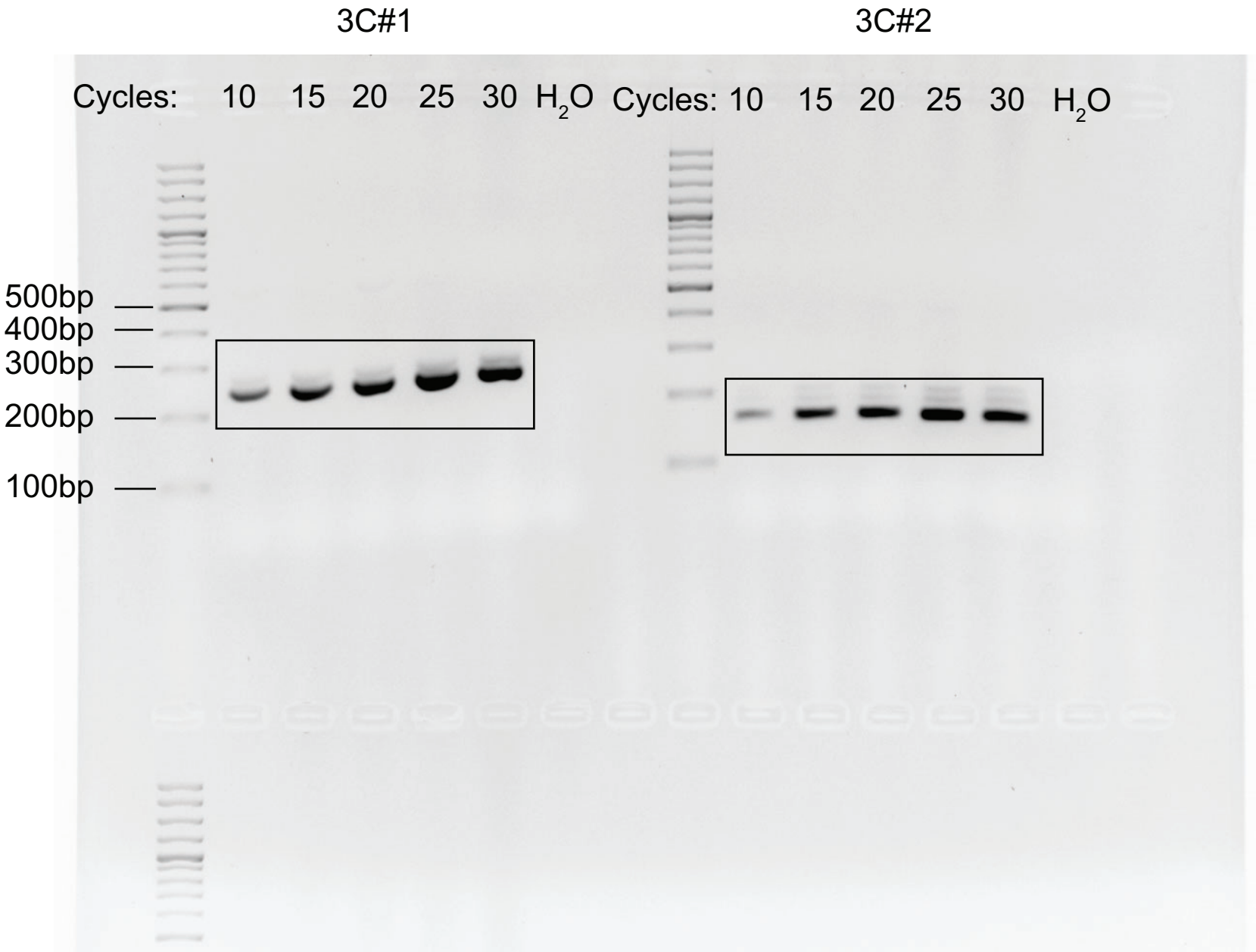

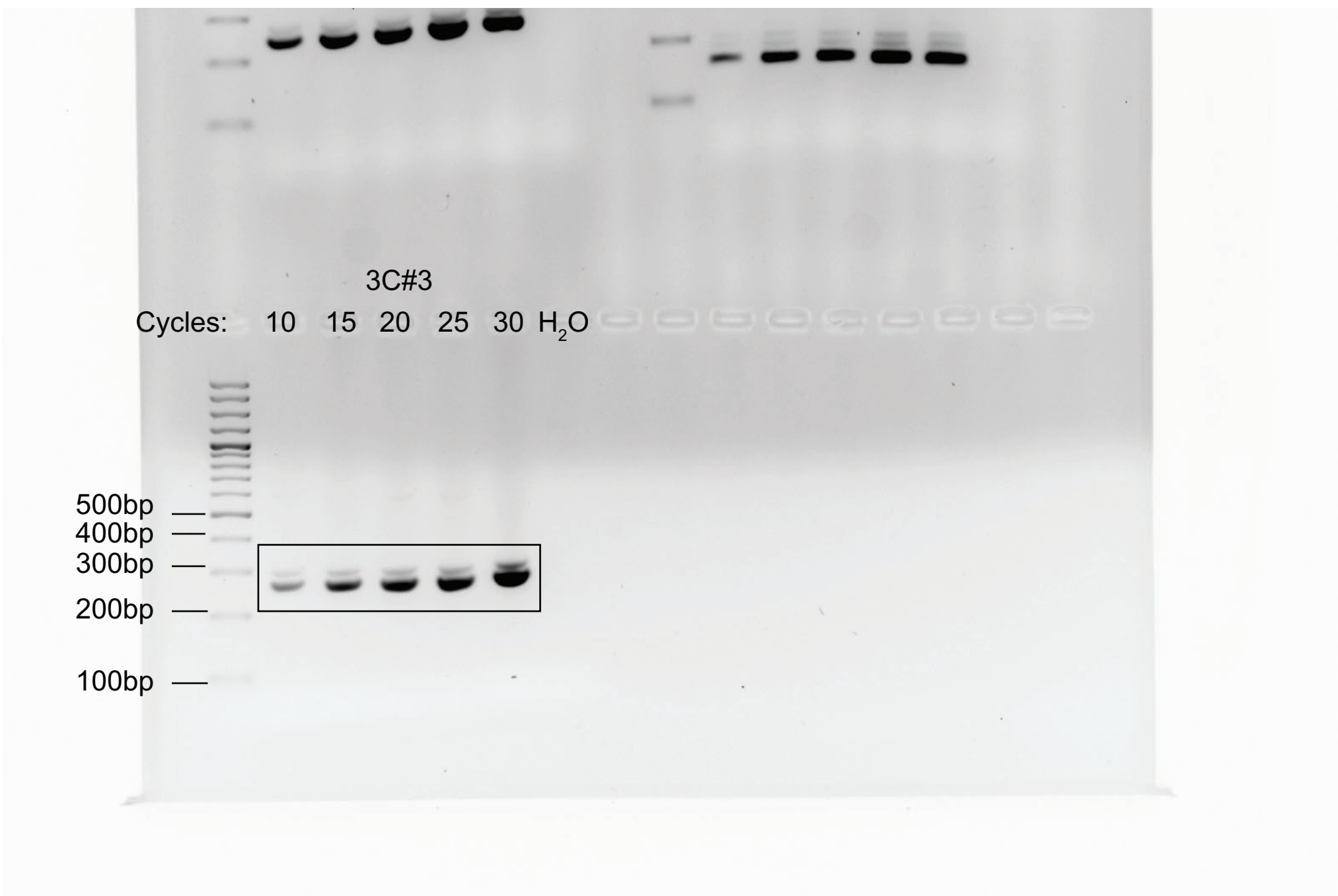

Full unedited blot for Figure S1B - Vinculin

Uncropped

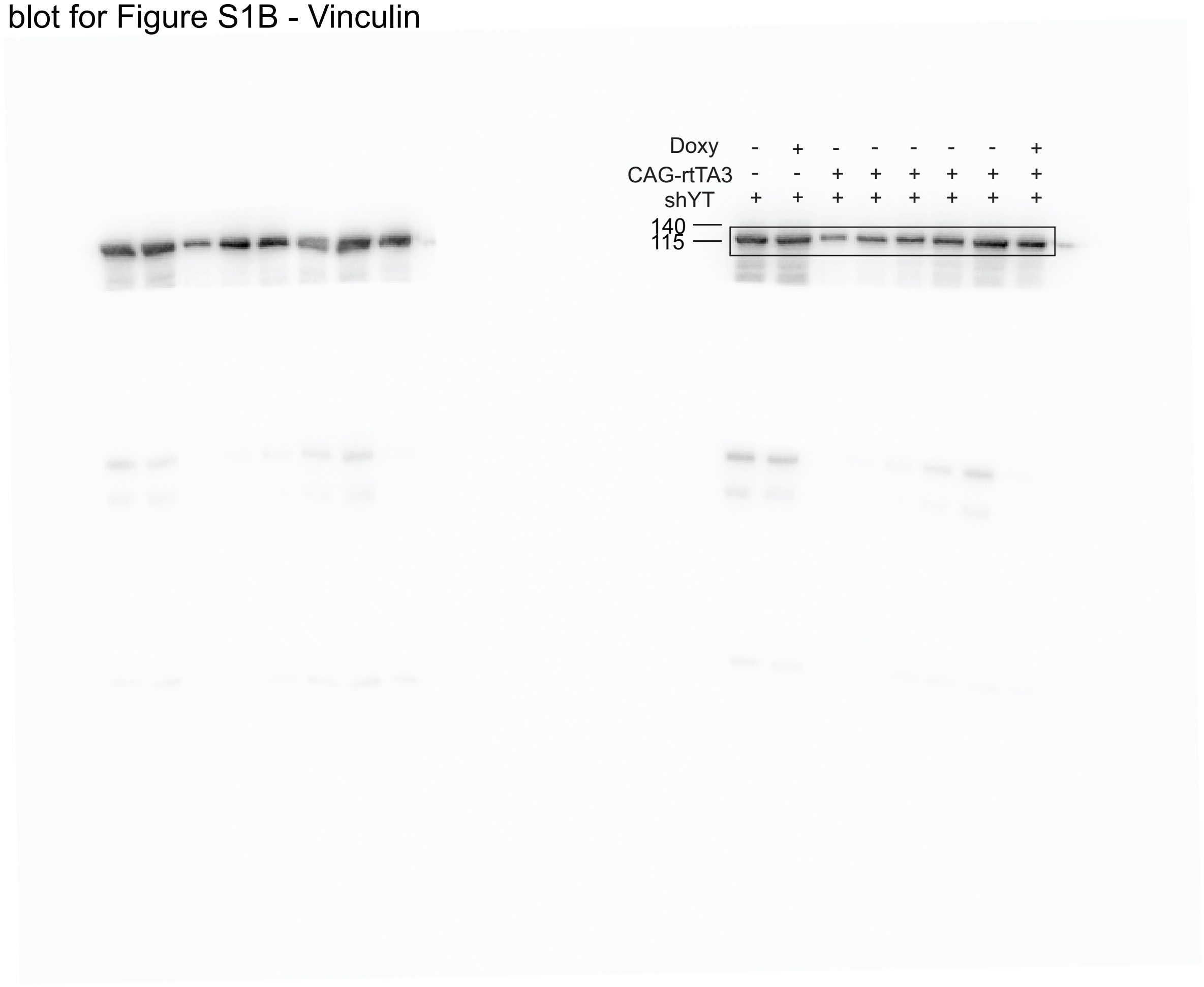

Merged

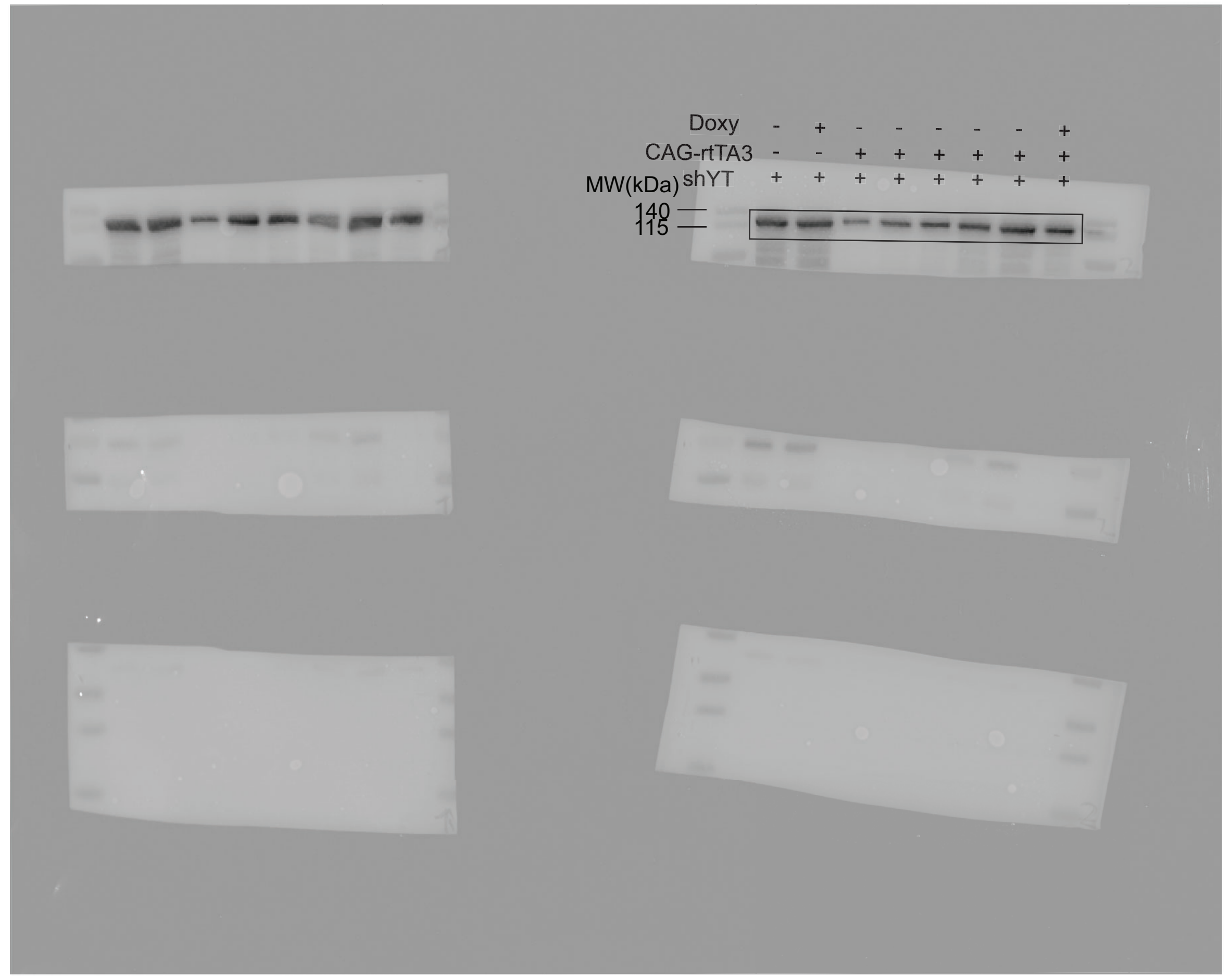

Full unedited blot for Figure S1B - YAP/TAZ

Uncropped

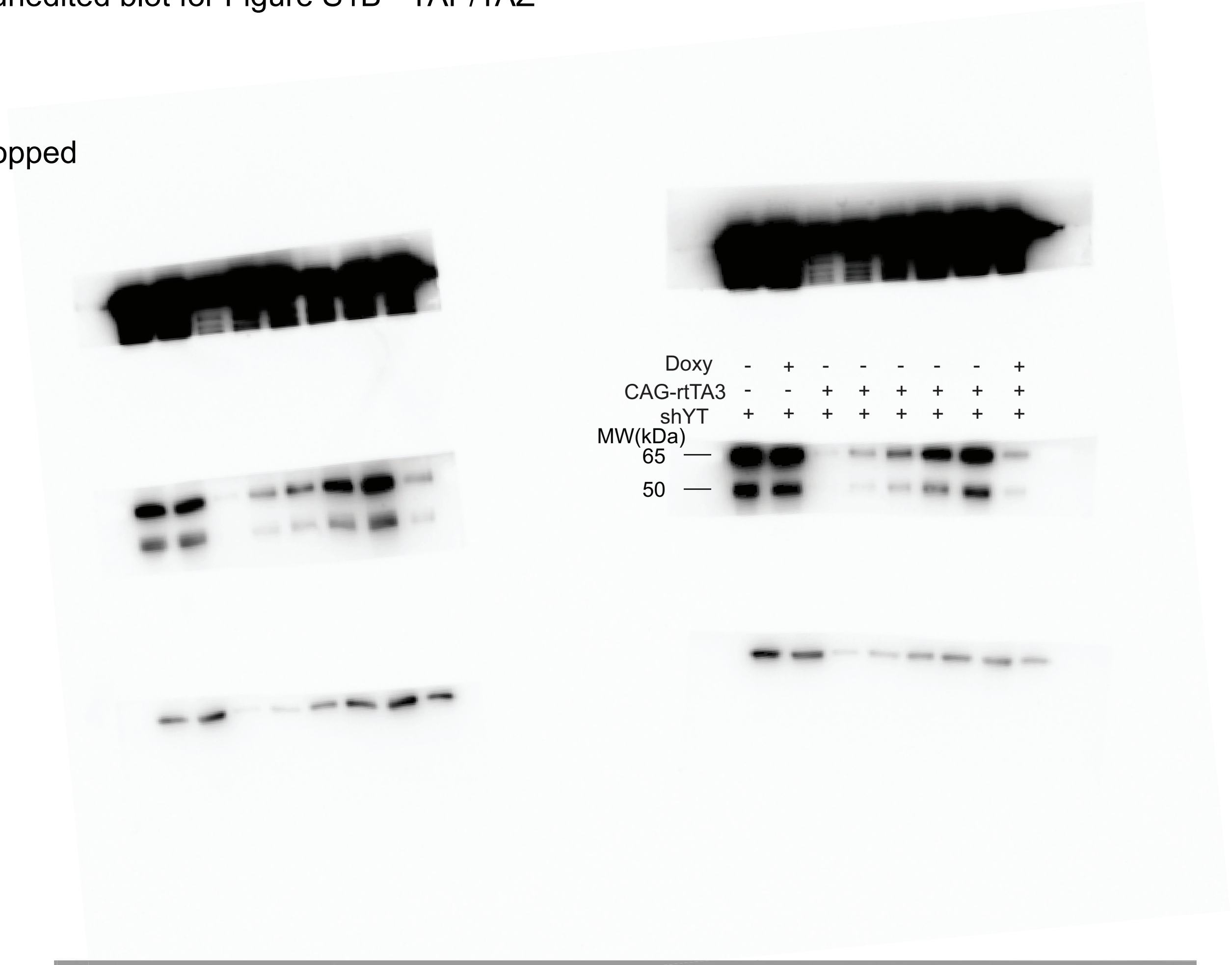

Merged

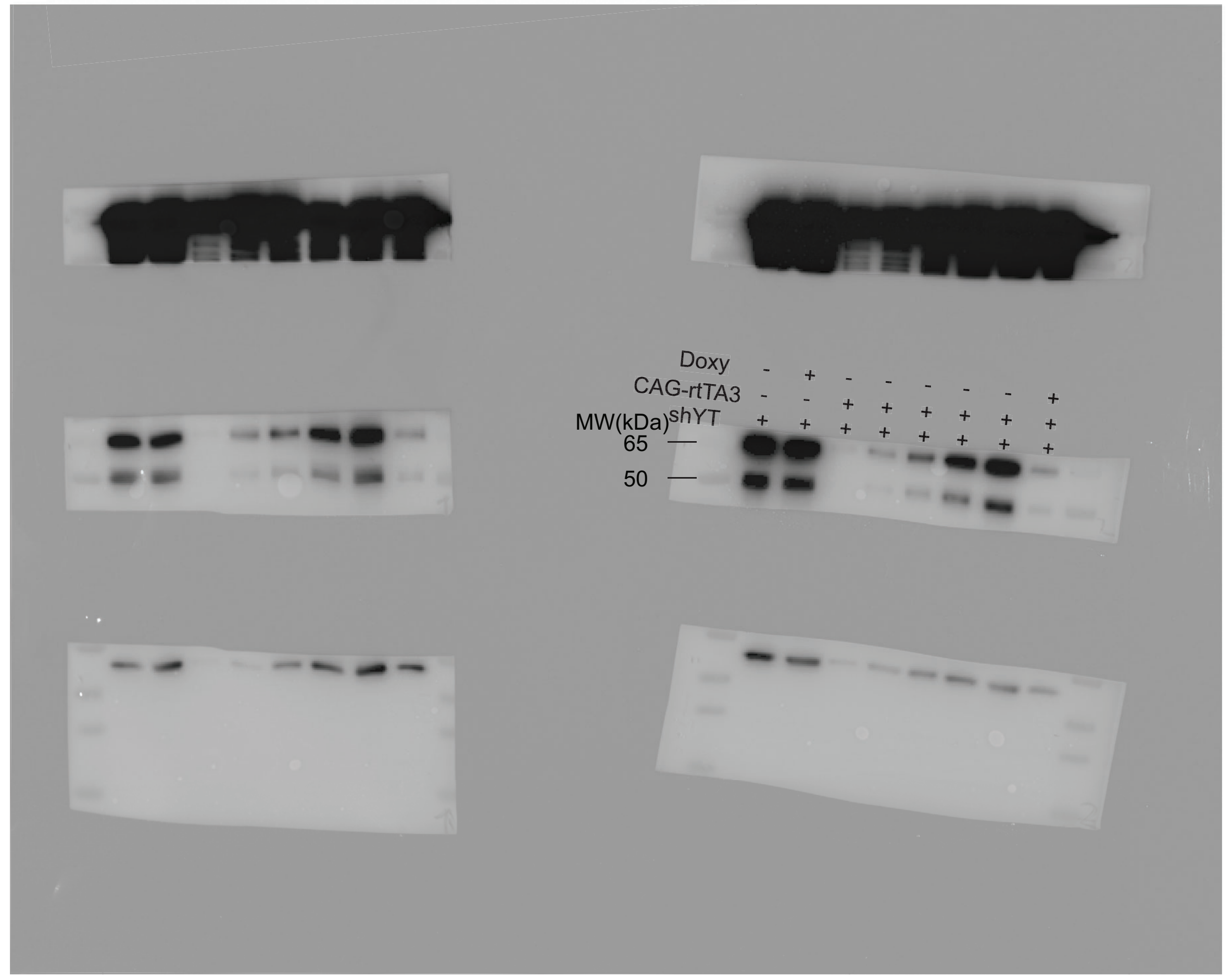

Full unedited blot for Figure S1B - CTGF

Uncropped

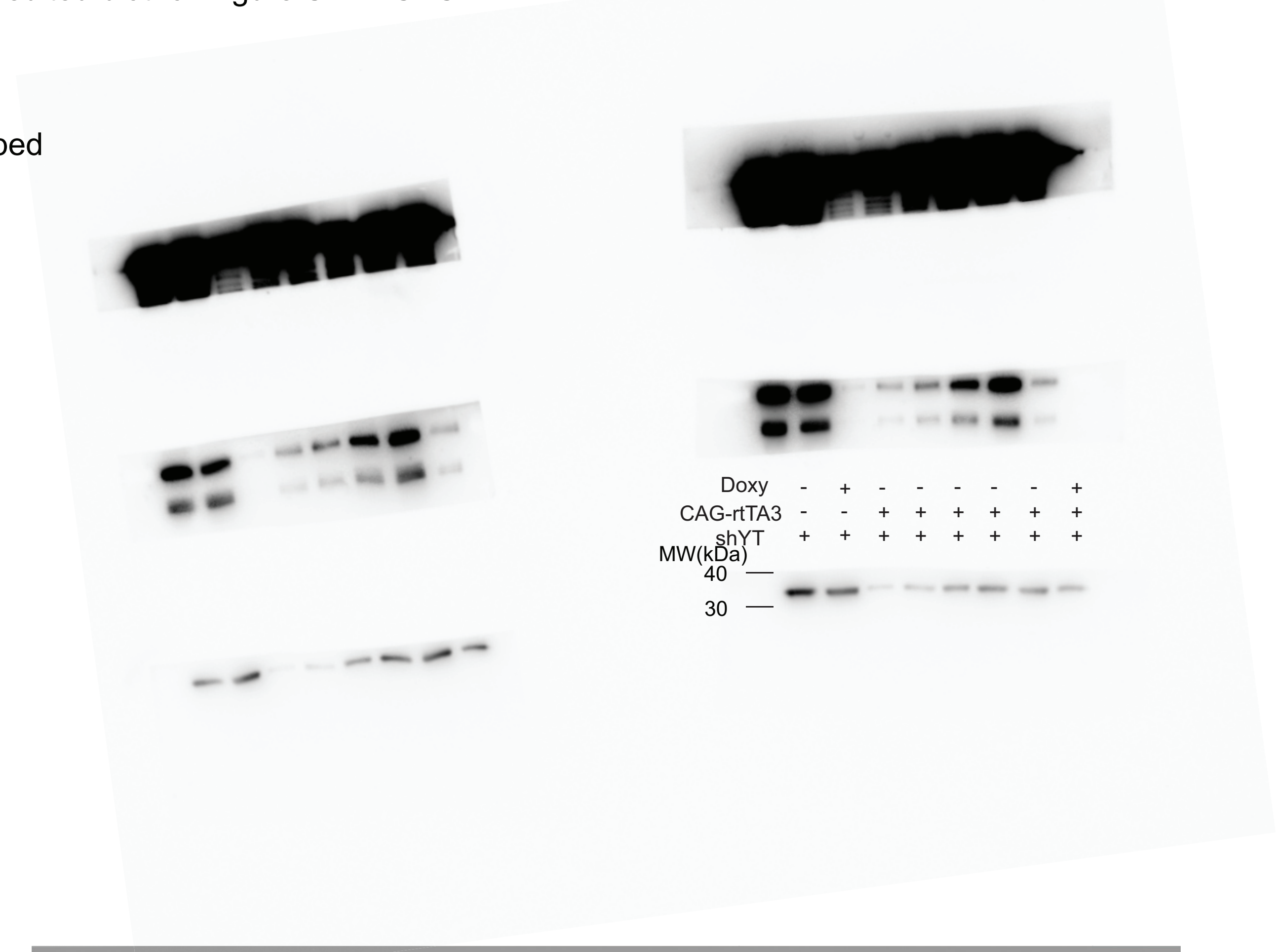

Merged

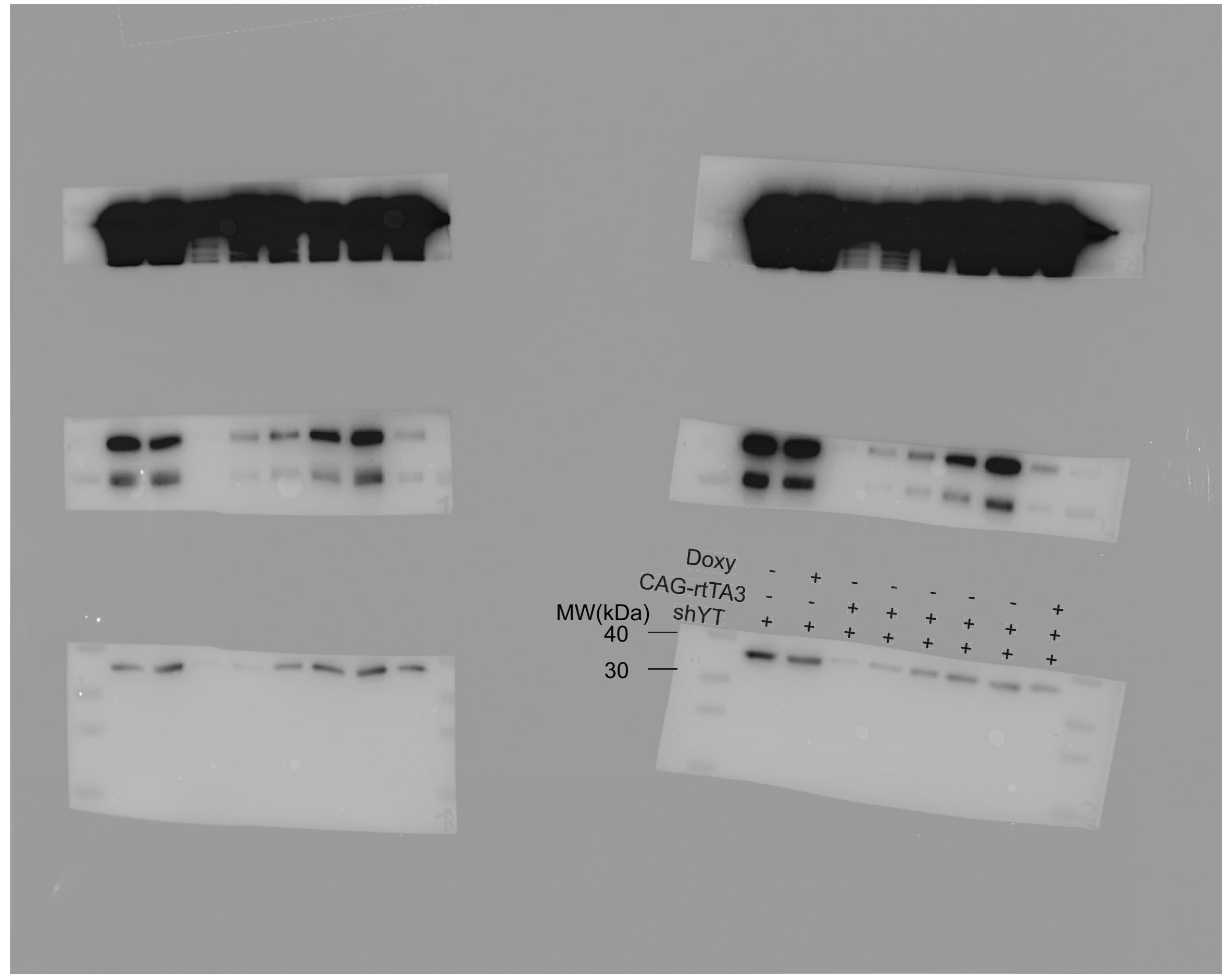

Supplement: Supplementary file 2 [file jci-136-201579-s273.pdf]
